# Unveiling photophysical mechanisms of NIR-II AIE luminogens for multimodal imaging-navigated synergistic therapies

**DOI:** 10.1093/nsr/nwaf254

**Published:** 2025-06-24

**Authors:** Jun Zhu, Yiqi Zhu, Yuxun Ding, Jianhong Huang, Jiangao Li, Jianquan Hou, Lei Wang, Ben Zhong Tang, Dong Wang

**Affiliations:** Center for AIE Research, Shenzhen Key Laboratory of Polymer Science and Technology, Guangdong Provincial Key Laboratory of New Energy Materials Service Safety, College of Material Science and Engineering, Shenzhen University, Shenzhen 518060, China; College of Physics and Optoelectronic Engineering, Shenzhen University, Shenzhen 518060, China; Department of Urology, The Fourth Affiliated Hospital of Soochow University, Medical Center of Soochow University, Suzhou Dushu Lake Hospital, Suzhou 215123, China; Center for AIE Research, Shenzhen Key Laboratory of Polymer Science and Technology, Guangdong Provincial Key Laboratory of New Energy Materials Service Safety, College of Material Science and Engineering, Shenzhen University, Shenzhen 518060, China; Center for AIE Research, Shenzhen Key Laboratory of Polymer Science and Technology, Guangdong Provincial Key Laboratory of New Energy Materials Service Safety, College of Material Science and Engineering, Shenzhen University, Shenzhen 518060, China; Center for AIE Research, Shenzhen Key Laboratory of Polymer Science and Technology, Guangdong Provincial Key Laboratory of New Energy Materials Service Safety, College of Material Science and Engineering, Shenzhen University, Shenzhen 518060, China; Department of Urology, The Fourth Affiliated Hospital of Soochow University, Medical Center of Soochow University, Suzhou Dushu Lake Hospital, Suzhou 215123, China; Center for AIE Research, Shenzhen Key Laboratory of Polymer Science and Technology, Guangdong Provincial Key Laboratory of New Energy Materials Service Safety, College of Material Science and Engineering, Shenzhen University, Shenzhen 518060, China; School of Science and Engineering, Guangdong Basic Research Center of Excellence for Aggregate Science, Shenzhen Institute of Aggregate Science and Technology, The Chinese University of Hong Kong, Shenzhen (CUHK-Shenzhen), Shenzhen 518172, China; Center for AIE Research, Shenzhen Key Laboratory of Polymer Science and Technology, Guangdong Provincial Key Laboratory of New Energy Materials Service Safety, College of Material Science and Engineering, Shenzhen University, Shenzhen 518060, China

**Keywords:** aggregation-induced emission, NIR-II emission, photophysical mechanisms, structure–activity relationships, multimodal phototheranostics

## Abstract

Multimodal phototheranostics has been recognized as one of the most momentous advances in cancer treatment. Of particular interest is a single molecular species simultaneously featuring in multiple imaging and synergistic phototherapies; the development of such a molecular species is nevertheless a formidably challenging task. Herein, we innovatively designed and synthesized three aggregation-induced emission (AIE)-active molecules with emission in the second near-infrared (NIR-II) window, by employing 10*H*-indeno[1,2-*b*][1,2,5]thiadiazolo[3,4-*g*]quinoxalin-10-one as the electron acceptor, 4-(*tert*-butyl)-*N*-(4-(*tert*-butyl)phenyl)-*N*-phenylaniline as the electron donor, and different π-bridge moieties. One of those molecules, namely 4,12-bis(7-(4-(bis(4-(tert-butyl)phenyl)amino)phenyl)-2,3-dihydrothieno[3,4-b][1,4]dioxin-5-yl)-10H-indeno[1,2-b][1,2,5]thiadiazolo[3,4-g]quinoxalin-10-one (OTTITQ), is capable of affording long absorption and emission wavelengths, efficient type I reactive oxygen species generation, and high photothermal conversion efficiency. Quantum chemical calculations and molecular dynamics simulations substantiated the structure–activity relationship of the molecules, the excited-state energy dissipation pathways and the impact of intramolecular motions on photophysical properties, while elucidating the mechanism of the AIE phenomenon. Moreover, OTTITQ nanoparticles offer unprecedented performance on fluorescence–photoacoustic–photothermal trimodal imaging-navigated photodynamic–photothermal synergistic therapies for bladder cancer.

## Teaser

Guided by theoretical studies, a multifunctional molecule with emission in the second near-infrared window is designed and synthesized for multimodal imaging-navigated synergistic therapies towards bladder cancer.

## INTRODUCTION

Cancer has long been considered one of the greatest threats to human life globally due to difficulty in its early diagnosis, rapid malignant progression and swift metastasis at later stages [1]. Although traditional therapeutic approaches such as surgery, chemotherapy and radiotherapy play a pivotal role in cancer treatment, they face various challenges such as high recurrence rates, severe side effects and invasive procedures [2]. Phototherapy, a treatment modality that employs phototheranostic materials to address various superficial and localized tumors by using excited-state energy upon exposure to light, has attracted considerable attention by virtue of its minimally invasive characteristics, precise spatial and temporal control, remarkable efficiency and minimal toxicity [3,4]. In particular, as one of the significant approaches on the basis of phototherapy, multimodal phototheranostics that integrates fluorescence imaging (FLI), photoacoustic imaging (PAI), photothermal imaging (PTI), photodynamic therapy (PDT) and photothermal therapy (PTT) has shown inexhaustible and vigorous vitality in disease treatment as it can overcome the limitations of single diagnostic and therapeutic modality, to remarkably enhance treatment efficacy [5–7]. Activated by a single light source, multimodal phototheranostics enables precise diagnostic imaging coupled with potent therapeutic interventions. Its rapid advancement is attributed to its superior spatial and temporal selectivity, remarkable therapeutic efficacy and minimal therapeutic resistance [8,9]. For constructing multimodal phototheranostic systems, two primary strategies are employed. One is the traditional method of combining multiple single-function components, which is limited in its clinical application potential due to lengthy synthetic routes, complex preparation processes and poor reproducibility [10,11]. The other is the development of individual molecules with multiple functions, which is gradually becoming mainstream due to its clear structure, simple preparation and high reproducibility [12–15]. Additionally, in comparison with the visible spectrum and the initial near-infrared window (NIR-I, 700–900 nm), the second near-infrared window (NIR-II, 1000–1700 nm) offers fluorescence with reduced light scattering, diminished autofluorescence from tissues and enhanced penetration depth, all of which contribute to its superior performance in *in vivo* bioimaging [16–20]. Moreover, organic NIR-II molecules for phototheranostics have a broader clinical application prospect compared with inorganic NIR-II materials, due to their ease of chemical modification, well-defined structure and outstanding biocompatibility [21–27].

In this related context, aggregation-induced emission (AIE) luminogens (AIEgens) with twisted and propeller-like structural architecture represent an ideal template to balance several dissipation channels of excited energy, and finally afford versatile phototheranostic properties [28,29]. The extended intermolecular distance of AIEgens resulting from their unique conformation can evidently impair the π–π stacking, thus boosting the radiative decay-involved fluorescence emission and the intersystem crossing (ISC) pathway-involved reactive oxygen species (ROS) generation [30,31]. Meanwhile, relatively loose molecular packing of AIEgens can be achieved in aggregate state, which offers a flexible environment for the intramolecular motions and consequently preserves the photothermal property of AIEgens desirably [32]. Evidently, it is highly desirable to deeply understand the structure–activity relationship and photophysical mechanisms of AIEgens, and consequently acquire the molecular design philosophy for rationally regulating energy decay pathways and tactfully tailoring versatile phototheranostic agents, which yet remains significantly challenging.

**Scheme 1.**
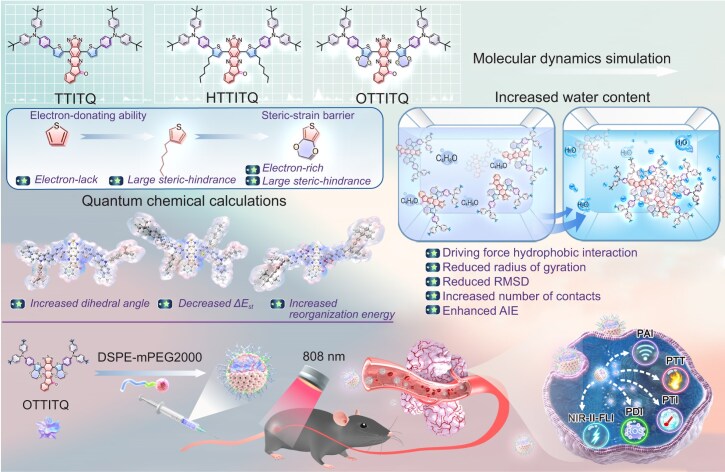
Schematic illustration of the design principle, theoretical calculation and multimodal phototheranostic applications.

To address this challenge, we developed three innovative AIEgens embodying donor–π–acceptor–π–donor (D–π–A–π–D) molecular configuration in this study (Scheme 1). These AIEgens are designed with 4-(*tert*-butyl)-*N*-(4-(*tert*-butyl)phenyl)-*N*-phenylaniline serving as the electron-rich donor moiety, 10*H*-indeno[1,2-*b*][1,2,5]thiadiazolo[3,4-*g*]quinoxalin-10-one (ITQ) as the electron-accepting skeleton and different π-bridge units. To gain profound insights into the structure–activity relationship of the molecules, quantum chemical calculations were conducted to evaluate the dihedral angles and the orbital energy level differences. The impact of intramolecular motion on non-radiative decay was elucidated by calculating the molecular reorganization energy, and predictions regarding the photothermal performance were made. Ultimately, molecular dynamics simulations were employed to deeply analyze the aggregation behavior of AIEgens in various solvent environments, shedding light on their AIE mechanism. Guided by theoretical calculations, the corresponding AIEgens were successfully designed and synthesized. Experimental validation confirmed that the optimal molecule 4,12-bis(7-(4-(bis(4-(tert-butyl)phenyl)amino)phenyl)-2,3-dihydrothieno[3,4-b][1,4]dioxin-5-yl)-10H-indeno[1,2-b][1,2,5]thiadiazolo[3,4-g]quinoxalin-10-one (OTTITQ) displayed typical AIE characteristics, bright NIR-II fluorescence emission, superior photothermal conversion efficiency and good ROS generation capability, which endowed OTTITQ nanoparticles (NPs) with unprecedented performance on NIR-II FLI/PAI/PTI trimodal-imaging-guided PDT/PTT synergistic therapy for bladder cancer.

## RESULTS AND DISCUSSION

### Quantum chemistry calculations

The key to achieving high-performance NIR-II chromophores is the design of chromophore structures with strong electron donor–acceptor (D–A) interaction, which facilitates intramolecular charge transfer, thereby reducing the bandgap and extending the absorption/emission wavelengths [33]. However, in recent years, the application of potent electron-deficient acceptors, such as benzobisthiadiazole (BBTD) and 6,7-diphenyl-[1,2,5]thiadiazolo[3,4-*g*]quinoxaline (DPTQ), has been relatively restricted and undiversified, which has hindered both structural diversity and further enhancement function of NIR-II chromophores [34]. To overcome this limitation, we adopted a novel protocol that employs the ITQ unit, containing the thiadiazolo[3,4-*g*]quinoxaline (TQ) moiety as an acceptor with high electron affinity [35,36]. In addition, we have incorporated an indanone moiety that contains a carbonyl group to augment the π-conjugation within the acceptor framework, which enhances the intramolecular D–A interaction [37,38]. With 4-(tert-butyl)-*N*-(4-(tert-butyl)phenyl)-*N*-phenylaniline as the donor, as well as thiophene, 3-hexylthiophene, and 2,3-dihydrothieno[3,4-*b*][1,4]dioxine groups as the π-bridges, TTITQ, HTTITQ and OTTITQ with D–A–D structural features were designed (Scheme 1).

**Figure 1.**
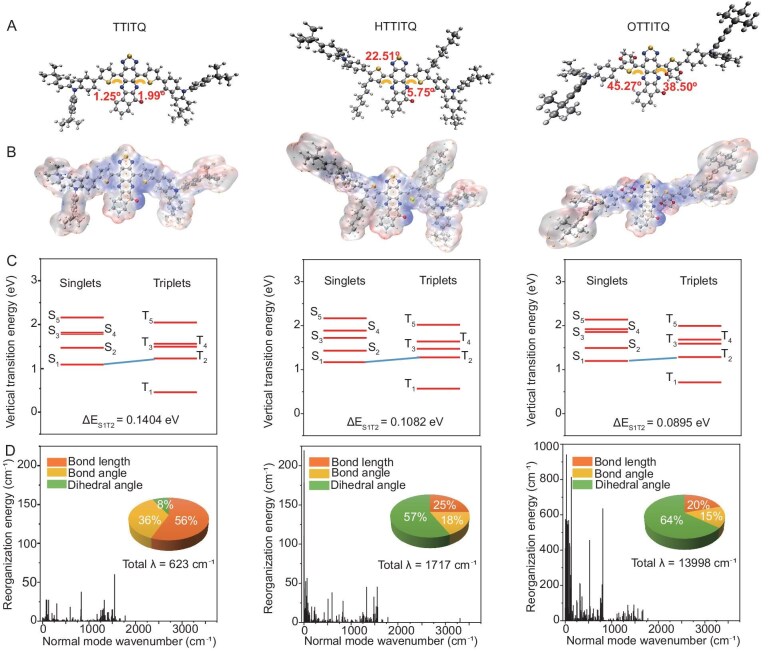
Quantum chemistry calculations. (A) Optimized structures. (B) Surface electrostatic potential. (C) Singlet and triplet energy levels and Δ*E*_ST_ values. (D) Calculated reorganization energy.

To forecast the photophysical properties of chromophores theoretically, we conducted quantum chemical calculations and dynamic simulations, which were essential for understanding the mechanisms of excited-state energy dissipation, and the influence of intramolecular motion on the photophysical properties of chromophores. Employing density functional theory (DFT) calculations at the B3LYP/6-31G(d) level, the geometric optimizations on TTITQ, HTTITQ and OTTITQ molecules were performed. The optimization results revealed that the dihedral angles between the ITQ core and the attached π-bridge (thiophene, 3-hexylthiophene and 2,3-dihydrothieno[3,4-*b*][1,4]dioxine moieties) in these molecules are 1.25°/1.99°, 5.75°/22.51° and 38.50°/45.27°, respectively (Fig. 1A). This trend of increasing angles is directly linked to the introduction of the π-bridge groups, which enhance the torsion angles between the donor/π-bridge and the acceptor core. Further analysis of the molecular orbitals unveiled that the electron density of the highest occupied molecular orbitals (HOMOs) is delocalized across the entire molecular framework. In contrast, the electron density of the lowest unoccupied molecular orbitals (LUMOs) is predominantly confined within the ITQ core (Fig. S1). In addition, the surface electrostatic potential energy for the three molecules was calculated, indicating that the surface electrostatic potential near the core is more positive, which suggests a stronger electron-deficient nature. Conversely, the surface electrostatic potential near the donor unit is more negative, revealing a robust electron-donating capability. These observations further substantiated the presence of distinct D–π–A–π–D interactions and intramolecular charge transfer phenomena (Fig. 1B). Subsequently, time-dependent DFT (TD-DFT) was employed to calculate the Δ*E*_ST_ values of the three molecules, aiming to elucidate their potential for ROS generation. The outcomes showed that OTTITQ has the lowest Δ*E*_ST_ value (0.0895 eV), which is lower than those of TTITQ (0.1404 eV) and HTTITQ (0.1082 eV) (Fig. 1C). A lower Δ*E*_ST_ value could be conducive to a higher ISC efficiency between molecules [39]. Moreover, the impact of intramolecular motion on non-radiative decay was investigated to reveal the photothermic properties of chromophores. Through the computation of the reorganization energies of three molecules, the contribution of each intramolecular motion mode was comprehensively analyzed. It was found that the OTTITQ molecule (13 998 cm^−1^) exhibits a significantly higher total reorganization energy, surpassing that of TTITQ molecule (623 cm^−1^) and HTTITQ molecule (1717 cm^−1^), indicating more vigorous intramolecular motions within the OTTITQ molecule. Furthermore, the contribution of the dihedral torsion mode to the total reorganization energy is markedly different among the three molecules. The OTTITQ molecule showed a higher proportion at 64%, in contrast to the TTITQ molecule at 8% and the HTTITQ molecule at 57%. The results suggest that the OTTITQ molecule is more twisted and more prone to rotation, which is critical for its non-radiative decay pathways. Remarkably, the reorganization energy of the OTTITQ molecule is predominantly distributed in the low-frequency region. This characteristic implies that the OTTITQ molecule could afford the highest photothermal conversion efficiency under photoexcitation conditions (Fig. 1D).

### Molecular dynamics simulations of chromophores

**Figure 2.**
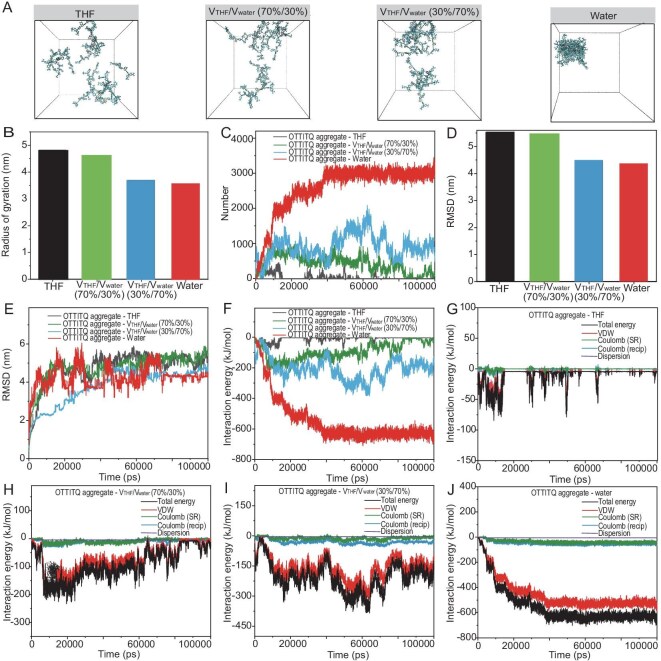
Molecular dynamics simulation. (A) Snapshots of amorphous aggregates obtained by molecular dynamics simulations of OTTITQ aggregate in different proportions of solvent. (B) Bar graph of radius of gyration. (C) Change in the number of atomic contacts between the innermost of OTTITQ aggregate and the outer of OTTITQ aggregate with time. (D) Bar graph of RMSD. (E) Change in RMSD with time. The interaction energy change with time of OTTITQ aggregate (F) in different proportions of solvent: (G) THF, (H) (V_THF_/V_water_) = 70%/30%, (I) (V_THF_/V_water_) = 30%/70%, (J) water. V: volume; VDW: van der Waals force.

The AIE curve reflects the changes in fluorescence intensity of chromophores varying with the composition of solvent mixtures, which are composed of a good solvent and a poor solvent in different proportions. To delve deeper into the luminescence mechanism of AIE, the AIE curve and the aggregation characteristics of chromophores, we conducted molecular dynamics simulations using different ratios of water and tetrahydrofuran (THF) as the solvent environment. Employing the GROMACS software, we established four distinct solvent environments to simulate these conditions: pure THF; 70%/30% mixture of THF/water; 30%/70% mixture of THF/water; and pure water. Within each environment, 20 OTTITQ molecules were introduced and we carried out molecular dynamic simulations using the standard amber force field [40]. Throughout the simulation process, snapshots of the optimized aggregate structures were selected to visualize the spatial arrangement of the OTTITQ molecules in their aggregated state. The results indicated that as the proportion of water increased, the degree of OTTITQ molecule aggregation also increased (Fig. 2A, Figs S2–S5). Furthermore, the potential energy changes during the aggregation process of OTTITQ molecules in solvents with different ratios of water to THF were analyzed. It was found that in pure THF and a 70%/30% mixture of THF/water solvent environment, the potential energy of the system remained relatively constant, suggesting that the molecules were evenly dispersed without significant aggregation. In contrast, in pure water and a 30%/70% mixture of THF/water environment, the potential energy of the OTTITQ system significantly decreased and approached equilibrium, indicating aggregation behavior (Fig. S6). To delve deeper into the molecular stacking patterns, the gyration radius (Rg) of OTTITQ aggregates in solvents with different ratios of water to THF were calculated to evaluate the compactness of the aggregates. The results showed that the value of Rg for OTTITQ aggregates in environments of pure THF, 70%/30% mixture of THF/water, 30%/70% mixture of THF/water and pure water were 4.83, 4.62, 3.70 and 3.56 nm, respectively. The progressive reduction in the value of Rg with increasing water content suggested a corresponding increase in the compactness of the molecular aggregates (Fig. 2B, Fig. S7). Additionally, as the water content increased, the number of atomic contacts between the innermost and outermost molecules of the aggregates also increased, further confirming that the increase in the water ratio promotes the aggregation of OTTITQ molecules (Fig. 2C, Fig. S8).

Substantially, root-mean-square deviation (RMSD) analysis for the conformational deviations in translation and rotation was conducted; the analysis revealed that in a pure THF environment, the RMSD values were the highest, suggesting greater molecular freedom. Conversely, as the proportion of water in the solvent mixture increased, the RMSD values decreased, indicating an increase in the degree of molecular aggregation, which restricts the rotation and translation of the molecules (Fig. 2D and E). In addition, the intermolecular interaction energies of OTTITQ aggregates in solvents with different ratios of water to THF were analyzed. The results showed that the intermolecular interaction energies for OTTITQ aggregates in environments of pure THF, 70%/30% mixture of THF/water, 30%/70% mixture of THF/water and pure water were −10, −94, −209 and −548 kJ/mol, respectively. This trend indicates that as the water content increased, the intermolecular interaction energy was also raised, with hydrophobic interactions contributing most significantly to the total energy, indicating that hydrophobic interactions are the main driving force for molecular aggregation (Fig. 2F–J, Table S1). In summary, the molecular dynamics simulations demonstrated that the increase in the water ratio in the solvent environment enhances intermolecular interactions, particularly hydrophobic interactions, which facilitates the aggregation of OTTITQ molecules. This aggregation significantly restricts the intramolecular rotation and translation, thereby promoting the radiative decay of excitions and ultimately inducing a strong fluorescence phenomenon.

### Synthesis and characterization of chromophores

**Figure 3.**
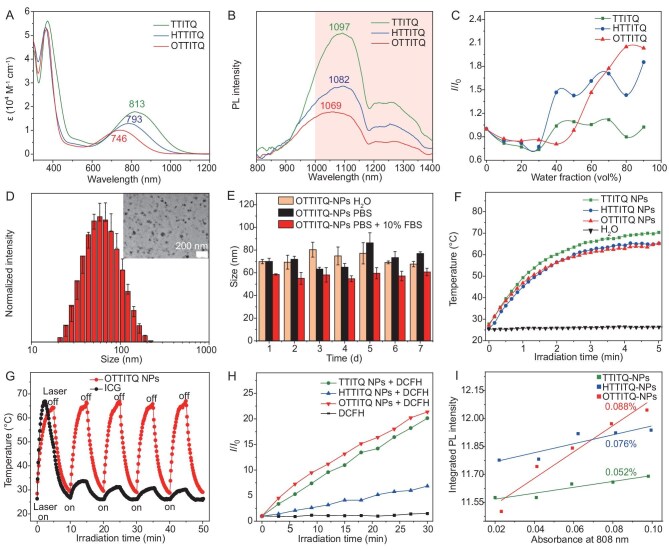
Characterization and properties of the obtained compounds. (A) Absorption spectra and (B) normalized PL spectra of the compounds in THF solution (5 μM). (C) Plots of relative PL intensity (*I*/*I*_0_) of TTITQ at 1097 nm, HTTITQ at 1082 nm and OTTITQ at 1069 nm versus water fraction. (D) DLS analysis of OTTITQ NPs. Inset: TEM image of OTTITQ NPs (mean ± SD, *n* = 3). (E) Stability analysis for size variation changes of OTTITQ NPs at room temperature in H_2_O, PBS or PBS + 10% FBS measured by DLS (mean ± SD, *n* = 3). (F) Photothermal conversion behaviors of AIEgen NPs (100 μM) in aqueous solution under 808 nm laser irradiation. (G) Photothermal stability of OTTITQ NPs upon 808 nm laser irradiation. (H) ROS generation of AIEgen NPs (2 μM) upon laser (808 nm, 0.8 W cm^−2^) irradiation for different times by using DCFH. (I) The integrated PL intensity (850–1500 nm) of different states of the AIEgen NPs versus different absorbance at 808 nm (IR-26, QY = 0.5% in dichloroethane).

Utilizing Suzuki coupling, Stille coupling and acid-catalyzed condensation reactions, these three molecules were obtained by connection of thiophene, 3-hexylthiophene and 2,3-dihydrothieno[3,4-*b*][1,4]dioxine fragments to the ITQ core, respectively, with the yields ranging from 57% to 99%. In particular, the unit of 4-(*tert*-butyl)-*N*-(4-(*tert*-butyl)phenyl)-*N*-phenylaniline not only acts as an electron donor to extend the absorption and emission wavelengths, but also functions effectively as a rotor due to its highly twisted conformation with multiple sigma bonds. These features confer upon the molecule the characteristic of a low electronic bandgap and the potential to act as a rotary motor for the conversion of thermal energy. All intermediates and final products have been analyzed and confirmed using nuclear magnetic resonance (NMR) and high-resolution mass spectrometry (HRMS) techniques (Figs S9–S29). Initially, we investigated the fundamental photophysical properties of these three molecules using ultraviolet-visible-near-infrared (UV-Vis-NIR) spectroscopy and photoluminescence (PL) spectroscopy. The results indicated that these molecules exhibited maximum absorbance at 813, 793 and 746 nm, with corresponding molar absorption coefficients (ε) of 1.786 × 10^4^, 1.290 × 10^4^ and 1.008 × 10^4^ M^−1^ cm^−1^, respectively (Fig. 3A), while the absorption and emission spectra of OTTITQ were notably blue-shifted in comparison with those of TTITQ and HTTITQ, likely attributed to the disruption of the planar molecular geometry by the 2,3-dihydrothieno[3,4-*b*][1,4]dioxine unit. Despite this, the maximum emission peak of OTTITQ at 1069 nm presents promising applications in the realm of NIR-II laser phototherapeutics (Fig. 3B). Further experimentation in various solvents demonstrated that obvious decrease in PL emission intensity and red-shifted maximum wavelengths were determined with the increment of polarity originating from the twisted intramolecular charge transfer (TICT) effect (Fig. S30). Employing a mixed solvent system of THF and water, the water fraction (*f*_w_) was systematically varied to explore the AIE characteristics of the synthesized molecules. The results indicated that in pure THF, the fluorescence of the three molecules was weak due to active intramolecular motion. As the water fraction in the solvent mixture increased, there was a progressive enhancement in the fluorescence intensity. This increase suggests a restriction in intramolecular motion upon aggregation, which in turn suppresses the TICT effect prevalent in water, demonstrating typical AIE characteristics (Fig. 3C, Fig. S31). Furthermore, molecular NPs were prepared using a nanoprecipitation method, employing the amphiphilic polymer DSPE-mPEG_2000_ as a matrix to encapsulate the molecules, thereby enhancing their water solubility. In water, the UV/Vis-NIR absorption spectrum and fluorescence emission spectrum of OTTITQ NPs exhibited a significant redshift compared with those in THF solution, with maxima at 781 and 1084 nm, respectively (Fig. S32). Dynamic light scattering (DLS) measurements revealed that OTTITQ NPs possessed excellent monodispersity in water, with a hydrodynamic diameter of approximately 56.06 nm. This size is favorable for accumulation at tumor sites through the enhanced permeability and retention (EPR) effect. Transmission electron microscopy (TEM) confirmed the uniform spherical shape of the NPs, noting a slight size reduction compared with DLS measurements. This variance is likely due to the loss of the hydration layer during the drying process for TEM sample preparation (Fig. 3D). The zeta potential of the NPs was determined to be −33.3 mV, indicating a significantly negative surface charge, which is crucial for enhancing colloidal stability and prolonging circulation times *in vivo* (Fig. S33) [41–43]. The polydispersity index (PDI) value of OTTITQ NPs is 0.243, which indicates that the OTTITQ NPs have good dispersity and uniformity. Notably, OTTITQ NPs maintained good stability even after being stored in water, phosphate buffered saline (PBS) or 90% PBS + 10% fetal bovine serum (FBS) solution for 7 days (Fig. 3E, Fig. S34).

Subsequently, we delved into the quantum yield (QY), photothermal behavior and ROS generation capacity of the NPs. Utilizing IR-26 (QY = 0.5% in dichloroethane) as a reference standard, the QYs of TTITQ NPs, HTTITQ NPs and OTTITQ NPs in aqueous solutions were determined to be 0.052%, 0.072% and 0.088%, respectively. OTTITQ NPs demonstrated the highest QY, a pivotal factor for achieving a high signal-to-noise ratio in fluorescence imaging (Fig. 3I, Figs S35 and S36). Next, the photothermal performance of NPs in aqueous solutions was evaluated. After irradiation with a laser (808 nm, 0.8 W cm^−2^, 5 min), the surface temperature of solutions containing 100 μM TTITQ NPs, HTTITQ NPs and OTTITQ NPs rapidly raised, attaining plateau temperatures of 70.4°C, 64.7°C and 65.2°C, respectively (Fig. 3F). This pronounced temperature elevation is associated with their twisted molecular configurations and rotor structures, which enable robust intramolecular motion. Notably, OTTITQ NPs demonstrated the superior photothermal conversion efficiency of 35.63%, significantly higher than the 25.91% of TTITQ NPs and 24.97% of HTTITQ NPs. Furthermore, we observed a direct correlation between the temperature elevation of the NPs solutions and the material concentration, as well as the laser's power density, indicating that the heating effect can be precisely modulated by adjusting these parameters. Throughout five cycles of heating and natural cooling, aqueous solution of OTTITQ NPs maintained impressive stability, with the maximum temperature increase being minimal and virtually insignificant. In contrast, indocyanine green (ICG) exhibited a noticeable decrease in photothermal conversion efficiency on the heating–cooling curve (Fig. 3G, Figs S37–S42). Finally, the ROS generation capability of these NPs was examined. To evaluate their ROS generation efficiency in solution, dichlorofluorescein (DCFH) was used as indicator. The experimental findings revealed that after irradiation with a laser (808 nm, 0.8 W cm^−2^), DCFH alone did not produce fluorescence. However, in the presence of the NPs, the fluorescence intensity of DCFH at 525 nm increased with irradiation time. In particular, the ROS generation efficiency of OTTITQ NPs was higher than that of TTITQ NPs and HTTITQ NPs, exhibiting a nearly 21-fold enhancement in DCFH fluorescence intensity after 30 min of laser irradiation (Fig. 3H). Additionally, specific indicators were used to ascertain the types of ROS generated by the NPs, including 9,10-anthracenediyl-bis(methylene)dimalonic acid (ABDA) for singlet oxygen (^1^O_2_), dihydrorhodamine (DHR123) for superoxide (•O_2_^−^) and hydroxyphenyl fluorescein (HPF) for hydroxyl radical (•OH). The experimental data indicated that under irradiation with a laser (808 nm, 0.8 W cm^−2^), the absorption of ABDA by the NPs remained virtually unchanged over time, while the fluorescence intensity of HPF and DHR123 demonstrated an increase (Figs S43–S47). This suggests that the NPs are capable of efficiently generating •OH and •O_2_^−^ through type I pathways, which is crucial for PDT. The results demonstrate that the ISC process effectively occurs within the NPs, particularly highlighting the robust absorption capability of OTTITQ NPs within the NIR window. This feature enables their photoactive behavior to be activated by NIR light, facilitating deep tissue penetration while avoiding light damage to biological systems, which showcases significant potential for *in vivo* applications. In summary, the OTTITQ NPs, owing to their extended absorption and emission wavelengths, intense NIR-II fluorescence, exceptional photothermal performance and commendable ROS generation capability, are projected to emerge as NIR laser-activatable multimodal phototheranostics agents for subsequent cancer diagnosis and treatment research.

### *In vitro* phototherapy properties of OTTITQ NPs

**Figure 4.**
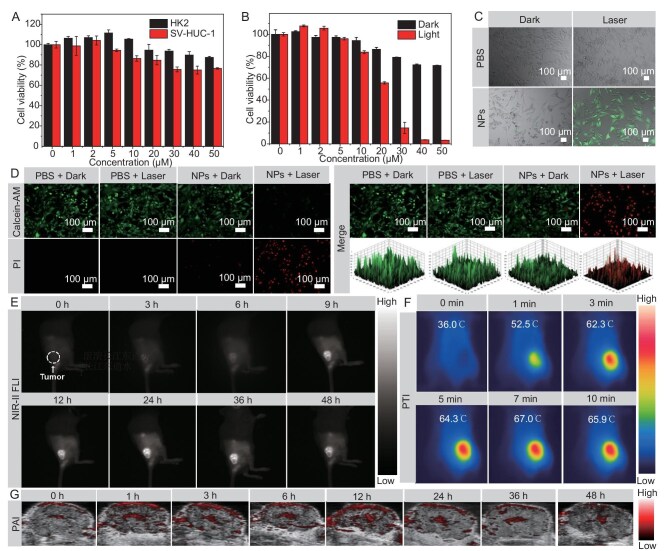
(A) Cell viabilities of HK2 and SV-HUC-1 cells after being treated with different concentrations of OTTITQ NPs under dark condition (mean ± SD, *n* = 3). (B) Cell viability of MB49 cells after being treated with different concentrations of OTTITQ NPs under dark or 808 nm laser irradiation (mean ± SD, *n* = 3). (C) Intracellular ROS generation of MB49 cells treated with OTTITQ NPs under 808 nm laser irradiation. (D) Live and dead cell staining of MB49 cells after various treatments. (E) NIR-II FLI of MB49 bladder cancer mice at different times after intravenous injection of OTTITQ NPs. (F) PTI of MB49 bladder cancer mice at different times after intravenous injection of OTTITQ NPs, followed by (24 h later) 808 nm laser irradiation for 0, 1, 3, 5, 7 and 10 min. (G) PAI of MB49 bladder cancer mice at different times after intravenous injection of OTTITQ NPs.

Motivated by the remarkable photophysical characteristics of OTTITQ NPs, we initially assessed their biocompatibility and phototherapy effects at the cellular level. Employing CCK-8 assays, for HK2 and SV-HUC-1 cells, it was found that OTTITQ NPs had low cytotoxicity under dark over the experimental concentration range 0–50 μM with cell viability rates remaining at greater than 75% (Fig. 4A). For MB49 cells, OTTITQ NPs demonstrated low cytotoxicity under non-irradiated conditions; cell viability rates remained at greater than 70% at 50 μM. Under laser irradiation (808 nm, 0.8 W cm^−2^, 5 min), the cell viability of MB49 cells was markedly reduced with increasing NPs concentration. When the concentration of OTTITQ NPs reached 40 μM, over 95% of the cells were effectively killed, highlighting the remarkable killing capacity of OTTITQ NPs against cancer cells upon photoactivation (Fig. 4B). For comparative analysis, cells were divided into four groups: control group, PBS; PBS + laser (L);OTTITQ NPs; and the experimental group OTTITQ NPs + L (laser: 808 nm, 0.8 W cm^−2^). To evaluate the phototherapy effects of OTTITQ NPs, we performed live/dead cell staining experiments on MB49 cells using Calcein-AM (for green fluorescence staining of live cells) and propidium iodide (PI, for red fluorescence staining of dead cells). Green fluorescence from Calcein-AM was observed in all three control groups, indicating cell survival. In contrast, the experimental group OTTITQ NPs + L exhibited noticeable red fluorescence from PI, indicating cell death (Fig. 4D). Additionally, the generation of intracellular ROS was monitored using a DCFH diacetate (DCFH-DA) fluorescence probe. The experimental group OTTITQ NPs + L exclusively exhibited bright green fluorescence, signifying the production of ROS, a phenomenon not observed in the other control groups. The results indicate that under photoactivated conditions, the NPs facilitate the generation of substantial ROS within MB49 cells (Fig. 4C). These findings confirm that OTTITQ NPs exhibit superior synergistic effects in PDT and PTT under 808 nm laser irradiation, while also validating the safety of the applied laser power and the controllability of the light-activated therapeutic strategy.

### *In vivo* imaging, antitumor effect and biosafety of OTTITQ NPs

To evaluate the *in vivo* application of OTTITQ NPs, we performed NIR-II FLI, PAI and PTI to guide synergistic PDT and PTT with MB49 bladder tumor-bearing mice by tail intravenous administration under 808 nm laser excitation with the long pass (LP) filter of 1000 nm. At 3 h post-injection, OTTITQ NPs passively accumulated at the tumor site, rendering the tumor tissue visibly discernible. At 24 h post-injection, the NIR-II fluorescence signal reached its peak, indicating that OTTITQ NPs peaked at the tumor site, then gradually diminished due to metabolic clearance. However, a distinct fluorescence signal persisted even after 36 h, indicating the prolonged retention of OTTITQ NPs (Fig. 4E, Fig. S48). *Ex vivo* NIR-II FLI further confirmed substantial fluorescence signals in both the tumor tissue and major metabolic organs, such as the liver and spleen (Fig. S49). Inspired by the remarkable photothermal conversion efficiency of OTTITQ NPs, the photoacoustic (PA) signals of the mice were recorded, which clearly delineated the surface and internal structure of the tumor. Consistent with the NIR-II FLI results, the PA signals at the tumor site also intensified over time, peaking at 12 h post-injection, after which they declined due to metabolic effects (Fig. 4G, Fig. S50). Considering the highest tumor enrichment, 24 h post-injection was chosen as the optimal timepoint for phototherapy. The PTI images, captured by an infrared thermal imager, demonstrated that at 24 h post-injection, under laser irradiation (808 nm, 0.8 W cm^−2^), for the OTTITQ NPs experiment group, the tumor temperature rapidly increased from 36.0°C to 52.5°C within 1 min and plateaued at 65.9°C within 10 min (Fig. 4F). In contrast, for the PBS + L group, the temperature of tumor sites only rose slowly from 36.5°C to 47.5°C under continuous laser exposure, which indicated that the heating effects of the 808 nm laser were almost negligible (Figs S51 and S52). Notably, during irradiation, the tumor site temperature was significantly higher than that of the surrounding normal tissue, indicating the tumor-selective delivery and site-specific photothermal effects of OTTITQ NPs, thus minimizing thermal damage to adjacent healthy tissues. These experiments affirm the superior NIR-II FLI, PAI and PTI synergistic imaging-guided capabilities of OTTITQ NPs.

**Figure 5.**
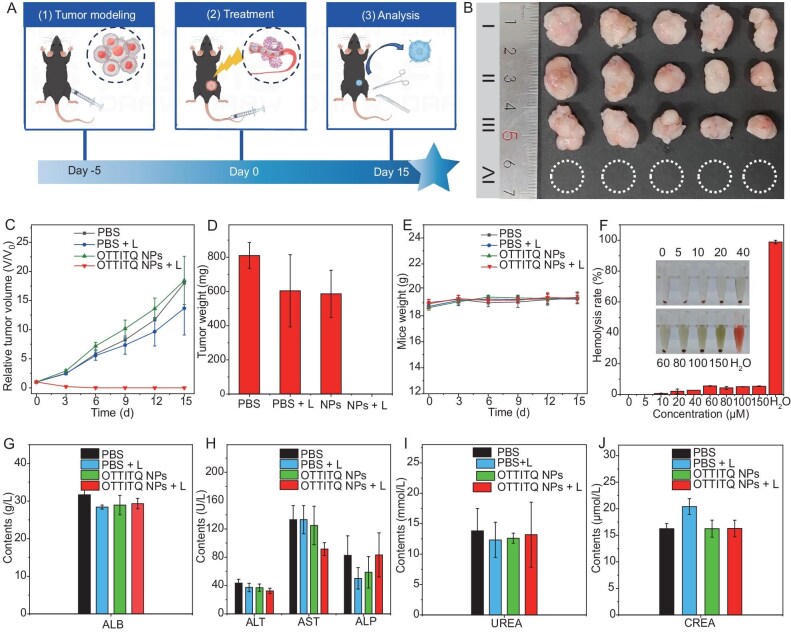
(A) The schematic diagram of treatment of MB49 bladder cancer mice. (B) Photograph of the tumors for different treatments at Day 15. (I) PBS; (II) PBS + L; (III) OTTITQ NPs; (IV) OTTITQ NPs + L (mean ± SD, *n* = 5). (C) Tumor growth curves after various treatments for 15 days (mean ± SD, *n* = 5). (D) Average tumor weight of tumor tissue after 15 days of treatments (mean ± SD, *n* = 5). (E) Weight growth curves after various treatments for 15 days (mean ± SD, *n* = 5). (F) Hemolysis rate of red blood cells treated with different concentrations of OTTITQ NPs by using water as a positive control and PBS as a negative control. (G and H) Hepatic function markers. (I and J) Renal function markers.

Ultimately, we evaluated the therapeutic efficacy of NIR laser-activated OTTITQ NPs on MB49 bladder cancer. Model mice were randomly divided into four groups: PBS, PBS + L and OTTITQ NPs as control groups, and OTTITQ NPs + L as the experimental group. The treatment period was 15 days, and the mice weight and tumor size were measured every 3 days during the treatment period. After intravenous injection of PBS or OTTITQ NPs, laser irradiation (808 nm, 0.8 W cm^−2^, 10 min) treatment was performed at 24 h (Fig. 5A). The tumor growth curves revealed that the tumors in the experimental group had completely regressed by the third day, with no signs of recurrence observed after 15 days of treatment, attributed to the precise accumulation of OTTITQ NPs in the tumor and their superior PDT and PTT effects. In contrast, the tumors in the control group progressed, with the tumor volume increasing approximately 18-fold compared with the initial value after 15 days (Fig. 5B–D). To further elucidate the *in vivo* tumor treatment mechanism of OTTITQ NPs, histological and immunohistochemical analyses were conducted on tumor sections. Hematoxylin and eosin (H&E) staining revealed significant cytoplasmic vacuolation and nuclear condensation, indicative of substantial tissue destruction induced by the NPs under laser irradiation. In contrast, tumor cells in the three control groups appeared in a vibrant and orderly arrangement. Immunofluorescence staining, including Tunel, CD31 and Ki67 assays, further demonstrated that the laser irradiation of OTTITQ NPs triggered substantial cell death, inhibited angiogenesis and mitigated tumor cell proliferation (Fig. S53). Additionally, the *in vivo* biocompatibility of OTTITQ NPs was systematically evaluated, with no hemolysis observed, even at concentrations as high as 150 μM OTTITQ NPs (Fig. 5F). H&E staining of major organs (heart, liver, spleen, lung, kidney) showed no signs of tissue damage or inflammation (Fig. S54). The detailed biochemical indicators of the experimental group, including hepatic and renal function parameters, showed no significant differences compared with the other three control groups (Fig. 5G–J). Moreover, the body weight changes of mice in all four groups were within the normal range (Fig. 5E). These results collectively demonstrate that the combination of OTTITQ NPs and NIR laser irradiation has good biosafety and low systemic side effects in the treatment of MB49 bladder cancer.

## CONCLUSION

In summary, we successfully developed a multifunctional phototherapy platform based on a single NIR-II AIEgen OTTITQ, integrating a suite of diagnostic and therapeutic capabilities, including NIR-II FLI, PAI, PTI, type I PDT and PTT. OTTITQ featuring a twisted D–π–A–π–D structure that was integrated with molecular rotors, strong electron acceptors and π-bridges simultaneously affords strong intramolecular charge transfer, extended absorption/emission wavelengths and reduced Δ*E*_ST_, thereby promoting efficient ISC processes and enhancing the generation efficiency of type I ROS, as well as high photothermal conversion efficiency. Particularly, quantum chemical calculations systematically elucidated the structure–activity relationship of the molecule, the energy dissipation pathways of its excited states and the influence of intramolecular motion on its photophysical properties. Moreover, molecular dynamics simulations revealed the AIE mechanism. The molecular design in this study represents a philosophy of multi-dimensional modulation of excited-state energy, which refers to the improvement of phototheranostic outputs from intramolecular dimension (giving high D–A interaction strength to ensure long absorption/emission wavelengths), intermolecular dimension (offering twisted conformation to diminish/avoid intermolecular π–π stacking and enable violent molecular rotations) and aggregate dimension (providing multiple hydrophobic skeletons to diminish the negative influence of water on emission). Furthermore, the excellent biocompatibility and photophysical properties endowed OTTITQ NPs with effective internalization into MB49 bladder cancer cells and exceptional cell-killing ability under 808 nm laser irradiation. *In vivo* experimental results indicated that OTTITQ NPs can efficiently accumulate at the tumor site, achieving clear imaging through NIR-II FLI/PAI/PTI. Moreover, complete eradication of tumors was observed by means of a single intravenous injection of OTTITQ NPs followed by a single laser irradiation, which highlighted the synergistic therapeutic effect of type I PDT and PTT. This research not only provides the necessary theoretical foundation and mechanistic explanation for the design of multifunctional NIR-II phototheranostics, but also offers innovative guidance for the design of one-size-fits-all phototherapy agents, providing valuable reference for the practical application of cancer treatment.

## Ethics statement

All the animal experimental procedures in this work were processed according to the regulations of the Animal Ethical and Welfare Committee of Shenzhen University [SYXK(YUE)2022-0302].

## Supplementary Material

nwaf254_Supplemental_Files
