# Supplementary material for: Unveiling photophysical mechanisms of NIR-II AIE luminogens for multimodal imaging-navigated synergistic therapies
Source: Natl Sci Rev. 2025 Jun 24;12(8):nwaf254. doi: 10.1093/nsr/nwaf254 (PMC12409622; doi:10.1093/nsr/nwaf254)
Supplement: nwaf254_Supplemental_Files [file nwaf254_supplemental_files.zip › Supplementary data.pdf]

Supplementary Information

Unveiling Photophysical Mechanisms of NIR-II AIE Luminogens for Multimodal Imaging-Navigated Synergistic Therapies

Jun Zhu‡, Yiqi Zhu‡, Yuxun Ding‡, Jianhong Huang, Jiangao Li, Jianquan Hou, Lei Wang, Ben Zhong Tang and Dong Wang*

Dr. J. Zhu, Dr. Y. Ding, J. Huang, Dr. J. Li, Prof. L. Wang, Prof. D. Wang

Center for AIE Research, Shenzhen Key Laboratory of Polymer Science and Technology, Guangdong Provincial Key Laboratory of New Energy Materials Service Safety, College of Materials Science and Engineering, Shenzhen University, Shenzhen 518060, China

Dr. J. Zhu

College of Physics and Optoelectronic Engineering, Shenzhen University, Shenzhen 518060, China

Dr. Y. Zhu, Prof. J. Hou

Department of Urology, The Fourth Affiliated Hospital of Soochow University, Medical Center of Soochow University, Suzhou Dushu Lake Hospital, Suzhou 215123, China

Prof. B. Z. Tang

School of Science and Engineering, Guangdong Basic Research Center of Excellence for Aggregate Science, Shenzhen Institute of Aggregate Science and Technology, The Chinese University of Hong Kong, Shenzhen (CUHK-Shenzhen), Guangdong 518172, China.

‡These authors contributed equally to this work.

Contents

Experimental Procedures ······ S6
Calculation, Synthesis and Characterization ······ S17

Figure S1. DFT of the frontier molecular orbitals for TTITQ, HTTITQ and OTTITQ ······ S17

Figure S2. In molecular dynamics simulations, the graphical representation illustrates the progression of OTTITQ in THF ······ S18

Figure S3. In molecular dynamics simulations, the graphical representation illustrates the progression of OTTITQ (V_{THF}/V_{water}) = 70%/30% ······ S19

Figure S4. In molecular dynamics simulations, the graphical representation illustrates the progression of of OTTITQ (V_{THF}/V_{water}) = 30%/70% ······ S20

Figure S5. In molecular dynamics simulations, the graphical representation illustrates the progression of OTTITQ in water ······ S21

Figure S6. In the dynamics simulation, the variation of potential energy change with time for OTTITQ aggregate in THF, (V_{THF}/V_{water}) = 70%/30%, in (V_{THF}/V_{water}) = 30%/70% and in water ······ S22

Figure S7. In the dynamics simulation, the radius of gyration of OTTITQ aggregate in THF, (V_{THF}/V_{water}) = 70%/30%, (V_{THF}/V_{water}) = 30%/70% and water ······ S23

Figure S8. In the dynamics simulation, the number of atomic contacts between the innermost of OTTITQ aggregate and the outer of OTTITQ aggregate in THF, (V_{THF}/V_{water}) = 70%/30%, (V_{THF}/V_{water}) = 30%/70% and water ······ S24

Table S1. In the dynamics simulation, energy term of the average interaction energy under 100 ns for OTTITQ aggregate in THF, (V_{THF}/V_{water}) = 70%/30%, (V_{THF}/V_{water}) = 30%/70% and water ······ S25

General procedure for the synthesis of TTITQ, HTTITQ and OTTITQ · S26

Scheme S1. The structures and synthetic routes of TTITQ, HTTITQ and OTTITQ ······ S26

Figure S9. ^{1}H NMR spectrum of 2 ······ S32

Figure S10. ^{1}H NMR spectrum of 3 ······ S33

Figure S11. HRMS spectrum of 3 ······ S33

Figure S12. ^{1}H NMR spectrum of TTITQ ······ S34

Figure S13. ^{13}C NMR spectrum of TTITQ ······ S34

Figure S14. HRMS spectrum of TTITQ ······ S35

Figure S15. ^{1}H NMR spectrum of 6 ······ S35

Figure S16. ^{1}H NMR spectrum of 8 ······ S36

Figure S17. ^{13}C NMR spectrum of 8 ······ S36

Figure S18. HRMS spectrum of 8 ······ S37

Figure S19. ^{1}H NMR spectrum of HTTITQ ······ S37

Figure S20. ^{13}C NMR spectrum of HTTITQ ······ S38

Figure S21. HRMS spectrum of HTTITQ ··········S38
Figure S22. ^{1}H NMR spectrum of 10 ··········S39
Figure S23. ^{1}H NMR spectrum of 11 ··········S39
Figure S24. ^{1}H NMR spectrum of 12 ··········S40
Figure S25. ^{13}C NMR spectrum of 12 ··········S40
Figure S26. HRMS spectrum of 12 ··········S41
Figure S27. ^{1}H NMR spectrum of OTTITQ ··········S41
Figure S28. ^{13}C NMR spectrum of OTTITQ ··········S42
Figure S29. HRMS spectrum of OTTITQ ··········S42
Figure S30. Absorption and emission spectra of TTITQ, HTTITQ and OTTITQ in different solvents ··········S43
Figure S31. PL spectra of TTITQ, HTTITQ and OTTITQ in THF/H_2O with different H_2O fractions ··········S43
Figure S32. The absorption and emission spectra of OTTITQ NPs in aqueous solution ·········· ········S4 4
Figure S33. The zeta potential of freshly papered OTTITQ NPs ··········S44
Figure S34. Stability analysis for OTTITQ NPs ··········S45

Figure S35. UV-Vis-NIR absorption spectra of TTITQ NPs, HTTITQ NPs, OTTITQ NPs and IR-26 ···S46

Figure S36. The ultraviolet absorbance ranges from 0.2 to 1.0, pertaining to the fluorescence spectrum of TTITQ NPs, HTTITQ NPs, OTTITQ NPs and IR-26 ·· S47

Figure S37. Photothermal heating behaviors of TTITQ NPs ···························S48

Figure S38. Photothermal heating behaviors of HTTITQ NPs ·························S49

Figure S39. Photothermal heating behaviors of OTTITQ NPs ·························S49

Figure S40. Thermal images of TTITQ NPs ···S50

Figure S41. Thermal images of HTTITQ NPs ··S51

Figure S42. Thermal images of OTTITQ NPs ··S52

Figure S43. ROS generation of AIEgens NPs by using HPF, DHR123 and ABDA as an indicator for overall ROS detection ···S52

Figure S44. ROS generation of AIEgens NPs by using DCFH as an indicator for overall ROS detection ····· ··S53

Figure S45. ROS generation of AIEgens NPs by using HPF as an indicator for overall ROS detection ················· ···S54

Figure S46. ROS generation of AIEgens NPs by using DHR123 as an indicator for overall ROS detection ································ ·····································S55

Figure S47. ROS generation of AIEgens NPs by using ABDA as an indicator for overall ROS detection ·· ·····························S56

Figure S48. NIR-II fluorescence intensity of tumor as a function of time before (0 h, pre-injection) and after intravenous injection of OTTITQ NPs ························S57

Figure S49. Ex vivo NIR-II fluorescence images and intensity of major organs and tumor after intravenous injection with OTTITQ NPs for 60 h ·························S57

Figure S50. PA intensity of tumor as a function of time before (0 h, pre-injection) and after intravenous injection of OTTITQ NPs ···S58

Figure S51. PTI of MB49 tumor-bearing mice treated with PBS through intravenous injection ·· ·················S58

Figure S52. The corresponding statistic temperature elevation of the irradiated area (tumor sites)-time curves ································ ································S59

Figure S53. TUNEL, Ki67, CD31 and H&E, staining analyses of tumor tissues under various treatments ··························· ···S60

Figure S54. H&E staining analysis of major organs of MB49 bladder cancer mice after various treatment for 15 days ··········· ···S61

Figure S55. The NIR-II fluorescence intensity of OTTITQ NPs (1 mM) with different thicknesses of chicken tissues on top of the samples ······· ····························S62

Figure S56. Inflammation-related biomarkers: TNF-α and IL-6 ························S62

Table S2. Cartesian coordinates for DFT optimized structure of TTITQ ·············S63

Table S3. Cartesian coordinates for DFT optimized structure of HTTITQ ···········S66

Table S4. Cartesian coordinates for DFT optimized structure of OTTITQ ···········S71

Table S5. The values of orbital states S_1-S_5 and T_1-T_5 of TTITQ, HTTITQ and OTTITQ ···S75

Table S6. The values the spin-orbit coupling (SOC) constant of TTITQ, HTTITQ and OTTITQ ···S76

Supplementary References ···S77

1. Experimental Procedures

Main Materials

The initial reagents consisted of 4,7-dibromo-5,6-dinitrobenzo[c][1,2,5]thiadiazole, 2-bromothiophene, 4-(tert-butyl)-*N*-(4-(tert-butyl)phenyl)-*N*-(4-(4,4,5,5-tetramethyl-1,3,2-dioxaborolan-2-yl)phenyl)aniline, 1*H*-indene-1,2,3-trione, 2-bromo-3-hexylthiophene, 2,3-dihydrothieno[3,4-b][1,4]dioxine, NBS, n-BuLi (2.5M in THF), Bu_3SnCl, $Pd(PPh_3)_4$, iron powder, zinc powder, K_2CO_3, KF, Na_2SO_4, acetic acid, along with various solvents, which were sourced from J&K Chemicals, Macklin Chemicals, or Aladdin Industrial Corporation. These materials were used directly without any further purification. All anhydrous solvents were prepared by standard drying methods before application. Amphiphilic polymers, specifically 2-Distearoyl-sn-glycero-3-phosphoethanolamine-*N*-[methoxy (polyethylene glycol)-2000 ($DSPE\text{-}mPEG_{2000}$), were custom-synthesized by Xi'an Ruixi Biological Technology Co., Ltd. Fluorescent probes such as fluorescein diacetate (FDA), propidium iodide (PI), 2',7'-dichlorodihydrofluorescein diacetate (DCFH-DA), hydroxyphenyl fluorescein (HPF), and 9,10-anthracenediyl-bis(methylene)dimalonic acid (ABDA) were obtained from Sigma-Aldrich. Dihydrorhodamine 123 (DHR123) and LysoTracker Green were sourced from Thermo Fisher Scientific. The Cell Counting Kit-8 (CCK-8) and Hoechst 33342 dye were acquired from Dojindo Laboratories. Fetal bovine serum (FBS) and penicillin-streptomycin solution were procured from Gibco. A reactive oxygen species (ROS) assay kit was purchased from Beyotime Biotechnology. All the aforementioned chemicals were employed in their received form from their respective suppliers, without undergoing any additional purification processes.

Instruments

^1H and ^{13}C nuclear magnetic resonance (NMR) spectra were obtained using 400, 500, and 600 MHz NMR spectrometers. Chemical shifts are expressed in parts per

million (ppm), referenced to either tetramethylsilane or the residual solvent peak as an internal standard. High-resolution mass spectrometry (HRMS) analyses were performed on a Finnigan MAT TSQ 7000 mass spectrometer utilizing a matrix-assisted laser desorption/ionization time-of-flight (MALDI-TOF) technique. Optical absorption measurements were conducted with a PerkinElmer Lambda 950 spectrophotometer. Photoluminescence (PL) spectra were acquired using an Edinburgh FS5 and an FLS1000 fluorescence spectrophotometer to evaluate emission properties. Particle size distribution was determined by dynamic light scattering (DLS) with a Malvern Zetasizer Nano ZSP, offering insights into the colloidal stability and dispersity of the samples. Morphological analysis and nanoparticle (NP) size determination were carried out using transmission electron microscopy (TEM) on a HITACHI HT7700 instrument. Photodynamic and photothermal experiments were conducted under irradiation from infrared semiconductor lasers emitting at 808 nm, provided by Changchun Radium Photoelectric Technology. Thermal changes during these experiments were closely monitored with an E6 infrared camera from FLIR Systems. Cell viability was assessed using the Cell Counting Kit-8 (CCK-8) kit, and absorbance was measured at 450 nm using a BioTek microplate reader. Confocal laser scanning microscopy (CLSM) images were obtained by a ZEISS LSM900 system.

Density Functional Theory Calculations

The molecular configurations of TTITQ, HTTITQ, and OTTITQ were refined through the optimization of their ground-state geometries, employing density functional theory (DFT) with the B3LYP functional and 6-31g (d) basis set. To ascertain that the optimized structures correspond to energy minima, analytical frequency calculations were conducted at the same theoretical level. Additionally, time-dependent density functional theory (TD-DFT) calculations were performed using the B3LYP functional in conjunction with the 6-31g (d) basis set to determine the optimized excited-state geometries. These quantum chemical computations were executed with the Gaussian 16 software suite.[1] Furthermore, the reorganization

energy analysis for the molecules in their isolated phase was conducted with the Molecular Materials Property Prediction Package (MOMAP). [2-4]

Molecular Dynamics Simulations

The molecular structure of OTTITQ was optimized after optimization through DFT. Systems were prepared by placing either 20 molecules within a cubic box measuring 10 nm on a side in THF, (V_{THF}/V_{water}) = 70%/30%, (V_{THF}/V_{water}) = 30%/70% and water, respectively. Energy minimization of the systems was subsequently accomplished using the steepest descent algorithm. A 10 ns NVT ensemble simulation under vacuum conditions (T = 300 K) was then conducted to attain the amorphous conformations of the aggregates. The resulting conformations were solvated in a water box and further equilibrated via a steepest descent minimization step followed by a 10 ns NPT ensemble simulation (P = 1 atm, T = 300 K). Thereafter, the equilibrated systems underwent a 100 ns NPT ensemble simulation (P = 1 atm, T = 300 K) to study the behavior of OTTITQ aggregates in different solvent environment.

Pressure was regulated by a Parrinello-Rahman barostat, while temperature was maintained with a Velocity-rescale thermostat. Periodic boundary conditions were applied in all three dimensions of the simulation box. Parameters for the AIEgens were taken from the General Amber Force Field (GAFF), and water molecules were modeled using the TIP3P potential. Short-range electrostatic and van der Waals interactions were treated with a standard 10 Å cutoff distance. Long-range electrostatic interactions were handled by the Particle Mesh Ewald (PME) method. All bonds involving hydrogen atoms were constrained using the LINCS algorithm, and the time step was set at 1 fs for all simulations. All molecular dynamics simulations were carried out using the GROMACS 2021.5 software package. [5]

Preparation of AIEgens NPs

A mixture of THF containing AIEgens at a concentration of 1 mg in 1 mL was directly combined with ultrapure deionized water that had been premixed with DSPE-mPEG$_{2000}$ at a concentration of 10 mg in 9 mL. This solution was then subjected to vigorous sonication using a microtip probe sonicator (model XL2000,

Misonix Incorporated, New York) operated at 45% power for a duration of 2 minutes. The sonication was performed in a sample bottle immersed in a cold-water bath to maintain a low-temperature environment. After sonication, the mixture was transferred into dialysis tubing with a molecular weight cut-off (MWCO) of 3500 Daltons and dialyzed against ultrapure water for 24 h, with the water being changed every 2 h initially, and then every 4 h to ensure thorough removal of residual THF. Following dialysis, the AIEgens NPs suspensions were concentrated by ultrafiltration, and their concentrations were determined using standard absorption spectroscopy curves. The AIEgens NPs solutions were stored at 4 ℃ in a light-protected environment until further use.

Size and Morphology

The dimensions of the AIEgens NPs dispersed in an aqueous solution were characterized using DLS analysis, which provided insights into their size distribution. Additionally, TEM was used to visualize the morpholoy of OTTITQ NPs with a concentration of 100 μM.

Photothermal Performance Measurement of AIEgens NPs

AIEgens NPs with a concentration of 100 μM were subjected to persistent irradiation with a laser (808 nm, 0.8 W cm^{-2}). Temperature measurements were recorded at 10-second intervals using an infrared thermography camera until the temperature readings stabilized. Water irradiated by an 808 nm laser was set as control. Moreover, the photothermal conversion efficiency of AIEgens NPs in aqueous suspension was ascertained in accordance with established methodologies.[6-8] The photothermal conversion efficiency (η) was calculated using Equation (1):

$$\eta = \frac{hS(T_{max}-T_{surr})-Q_{dis}}{I(1-10^{-A}\lambda)} \qquad (1)$$

In this equation, S represents the surface area of the container, h is the heat transfer coefficient, T_{max} denotes the maximum steady-state temperature reached during experimentation, and T_{surr} signifies the ambient temperature. Q_{dis} accounts for the heat dissipated due to the laser's effect on both the solvent and the container. The

laser power I is set at 0.8 W cm^{-2} for an 808 nm wavelength, and A_λ refers to the absorbance of the AIEgens NPs aqueous suspension at the same wavelength. The value of hS can be determined by Equations (2-4):

$$hS = \frac{\sum_i m_i C_{p,i}}{\tau_s} \qquad (2)$$

$$t = -\tau_s \ln(\theta) \qquad (3)$$

$$\theta = \frac{T - T_{surr}}{T_{max} - T_{surr}} \qquad (4)$$

Here, τ_s denotes the time constant of the sample system, and m represents the mass of the solution, which includes both the solvent and the NPs. Given the negligible mass of NPs relative to water, the effective mass considered in the thermal capacity calculations is essentially that of water alone, recognizing water's significantly higher specific heat capacity compared to most materials. Consequently, the contributions of mass mi and specific heat $C_{p,i}$ from the NPs are disregarded, adopting values of m_{H2O} and $C_{p,H2O}$ as 0.2 g and 4.2 J/g for water, respectively. θ is a dimensionless term, representing the temperature driving force. Employing Equations (1-4) as a framework, the photothermal conversion efficiency (η) of TTITQ NPs, HTTITQ NPs and OTTITQ NPs in aqueous solution were calculated.

Photothermal Stability

The photothermal stability of the aqueous solutions containing AIEgens NPs and indocyanine green (ICG) was assessed under 808 nm laser irradiation. The samples were subjected to a series of irradiation cycles, with the laser being turned on and off to achieve five distinct heating and cooling phases. Throughout this process, the temperature fluctuations of both the AIEgens NPs and ICG were meticulously monitored to evaluate their thermal response and stability.

Relative Fluorescence QY Measurement

The determination of the quantum yield (QY) for the dyes was performed by referencing IR-26, a standard with a known QY of 0.5% [9]. A dilution series of IR-26 in 1,2-dichloroethane was prepared to achieve specific absorption intensities at 808 nm, spanning approximately from 0.02 to 0.1. The emission spectra were then

integrated over the 850-1500 nm range. This procedure was repeated for samples in water. The integrated emission data was plotted against absorption intensity to obtain a linear regression. The QY calculation was based on the equation:

$$QY_{sample(wavelength)} = QY_{ref} \cdot \frac{S_{sample\ (wavelength)}}{S_{ref}} \cdot (\frac{n_{sample(wavelength)}}{n_{ref}})^2$$

In this formula, $QY_{sample\ (wavelength)}$ indicates the quantum yield of the NPs within the 850-1500 nm wavelength range, QY_{ref} is the quantum yield of IR-26 in dichloroethane (0.5%), $S_{(sample)}$ and $S_{(ref)}$ are the slopes from the linear regression of the integrated emission spectra for the sample and IR-26, respectively, over the same wavelength interval. Additionally, $n_{(sample)}$ and $n_{(ref)}$ are the refractive indices of the solvents used water and 1,2-dichloroethane, respectively, which account for their impact on the optical properties of the materials being studied.

In Vitro Detection of Overall ROS Generation in Aqueous Solution

To assess the capacity of AIEgens NPs to produce ROS in solution, we utilized the well-established probe dichlorofluorescein (DCFH) as an indicator. The protocol for probe activation involved the combination of 2 mL of a 0.01 M NaOH aqueous solution with 0.5 mL of 0.001 M DCFH in ethanol solution, followed by gentle mixing in the absence of light at room temperature for approximately 30 min. The activated probe solution was then incorporated into 10 mL of phosphate-buffered saline (PBS) and stored at -20 ℃ in the dark until further use. For the ROS detection assays, the ROS-sensitive solution (4×10^{-5} M) was diluted to a final concentration of 5×10^{-6} M in separate aliquots containing AIEgens NPs at a concentration of 1×10^{-6} M. These mixtures were examined in a dark chamber using a PL spectrometer. The fluorescence of DCFH, induced by ROS upon (808 nm, 0.8 W cm^{-2}) laser irradiation, was monitored at various time points. PL spectra were collected with 488 nm excitation, and the emission spectra from 500 to 620 nm were carefully recorded. The fluorescence intensity peak at 525 nm was employed as a quantitative measure for evaluating the total ROS generation elicited by AIEgens NPs under the specified laser stimulation.

Detection of Type II $^1O_2$ in Aqueous Solution

To assess the production of singlet oxygen ($^1O_2$), we employed ABDA as a chemical probe. Initially, a concentrated ABDA solution with a molarity of 20 $\times 10^{-3}$ M was diluted to a working concentration of 20 $\times 10^{-6}$ M within a sample matrix that contained AIEgens NPs at a molarity of 1 $\times 10^{-6}$ M, with both components being suspended in an aqueous medium. Then the aqueous mixture of ABDA and AIEgens NPs was subjected to irradiation with a laser (808 nm, 0.8 W cm^{-2}). During the irradiation, the absorption spectrum of ABDA from 330 nm to 450 nm was meticulously monitored. Notably, the decrease in absorbance at 400 nm was meticulously recorded, which can quantify the decomposition rate of ABDA and serve as an accurate indicator of the rate of $^1O_2$ generation.

Detection of Type I •OH in Aqueous Solution

To evaluate the potential for hydroxyl radical (•OH) production, HPF was employed as a quantitative indicator. A concentrated solution of HPF, prepared at an initial molarity of 5 $\times 10^{-3}$ M in *N,N*-dimethylformamide (DMF), was diluted to a final concentration of 5 $\times 10^{-6}$ M prior to its incorporation into the experimental solution. This solution contained AIEgens NPs at a concentration of 1 $\times 10^{-6}$ M, both dispersed in PBS matrix. After irradiation with a laser (808 nm, 0.8 W cm^{-2}), the fluorescence emission spectrum of HPF from 500 nm to 620 nm was examined with excitation at 490 nm. The fluorescence intensity at 515 nm was specifically measured, serving as a proxy for the rate of •OH generation.

Detection of Type I $\bullet O_2^-$ in Aqueous Solution

To determine the generation of superoxide radicals ($\bullet O_2^-$), the compound DHR123 was utilized as a fluorescent probe. A primary stock solution of DHR123 was prepared at a concentration of 5 $\times 10^{-3}$ M, which was subsequently diluted to a final concentration of 5 $\times 10^{-6}$ M upon addition to the experimental mixture containing AIEgens NPs at a concentration of 1 $\times 10^{-3}$ M, both suspended in PBS buffer. The fluorescence emission of DHR123 from 500 nm to 620 nm was measured

with excitation at 495 nm after the irradiation with a laser (808 nm, 0.8 W cm^{-2}). The fluorescence intensity at 526 nm was documented as an indicator of the superoxide radical production rate.

Biocompatibility evaluation

HK2 and SV-HUC-1 cells were initially seeded in 96-well microplates at a concentration of 5×10^3 cells per well and grew for 24 h. Subsequently, the cells were exposed to various concentrations of OTTITQ NPs (0 1 2 5 10 20 30 40 50 μM) and co-incubated for another 24 h. Then CCK-8 assay was conducted to assess the cell viability. The absorbance was measured using a BioTek microplate reader at a wavelength of 450 nm. The data obtained were presented as the percentage of viable cells relative to the control group. The relative cell viability was calculated using the following formula: Cell viability (%) = $(OD_{sample}-OD_{background})/(OD_{control}-OD_{background}) \times 100\%$.

Dark toxicity and phototoxicity assay

MB49 were initially plated in 96-well microplates at a seeding density of 5×10^3 cells per well and grew for 24 h. Subsequently, the cells were subjected to various concentrations of OTTITQ NPs (0 1 2 5 10 20 30 40 50 μM). After incubation for 12 h, the cells were exposed to irradiation with a laser (808nm, 0.8 W cm^{-2}, 5 min). By contrast, the control group was incubated without laser irradiation to assess the dark toxicity. Afterwards, the cells were cultured for an additional 12 h and then the medium was replaced with CCK8 for 1 h. The cell viability was quantified using a microplate reader at an absorbance wavelength of 450 nm. The data were presented as the percentage of viable cells relative to the control group. The relative cell viability was calculated using the formula: Cell viability (%) = $(OD_{sample}-OD_{background})/(OD_{control}-OD_{background}) \times 100\%$.

Live-dead cell staining

MB49 cells were plated in a 96-well plate at a density of 5×10^3 cells per well and incubated for 24 h. Subsequently, OTTITQ NPs with the concentration of 50 μM

were added to the cultrure dished and co-incubated for 12 h. The cells were then rinsed with PBS, followed by exposure to irradiation with a laser (808nm, 0.8 W cm^{-2}, 5 min). Cells kept in the dark served as the control group. After incubation for 8 h, the cells were gently washed and stained with Calcein-AM/PI for 30 min. Afterwards, the cells were washed with PBS and then visualized using CLSM.

Intracellular ROS generation

MB49 cells were initially plated in a 96-well plate at a density of 5×10^3 cells per well and incubated for 24 h. After that, OTTITQ NPs with the concentration of 50 μM were introduced and co-incubated with MB49 cells for 12 h. Then the cells were exposed to irradiation with a laser (808nm, 0.8 W cm^{-2}, 5 min), and cell incubated in the dark was set as control. Next, the cells were treated with fresh, serum-free media containing 10 μM DCFH-DA at 37 ℃ and incubated for 20 min. Finally, the cells were rinsed with PBS for three times and visualized using CLSM.

Animals and tumor models

The animal protocol used was in accordance with the guidelines of the Institutional Animal Care and Use Committee (IACUC) of China and received approval from the Animal Ethical and Welfare Committee of Shenzhen University (SYXK(YUE)2022-0302). Female C57BL/6J mice (6 weeks) were purchased from Guangdong Medical Laboratory Animal Center (Guangdong, China). All the mice were housed in specific pathogen-free environment with sufficient water and food. Before the experiments, all the mice had one-week adaptation period. To establish the subcutaneous bladder cancer models, 1×10^6 MB49 cells were inoculated subcutaneously into the right lower back of C57BL/6J mice. 7−10 days after inoculation, when the tumor volumes reached approximately 100 mm^3, the mice were assigned randomly for the subsequent experimental procedures.

In vivo multi-modal imaging

For NIR-II fluorescence imaging, MB49 tumor-bearing mice were injected with OTTITQ NPs (200 μL, 1 mM) intravenously. After administration, the mice were

anesthetized with 2% isoflurane in oxygen. Subsequently, the mice were then imaged at 0, 3, 6, 9, 12, 24, 36, and 48 h post injection to track the in vivo distribution and metabolism of the OTTITQ NPs using the NIR-II in vivo imaging system with the long pass (LP) filter of 1000 nm and 808 nm laser. At the culmination of the 60-hour post-injection period, the mice were euthanized, and major organs (heart, liver, spleen, lungs, and kidneys) along with the tumor were excised for further NIR-II fluorescence imaging. In parallel, photoacoustic imaging was executed at the same time points (0, 1, 3, 6, 12, 24, 36, and 48 h post-injection) using the Vevo LAZR photoacoustic imaging system. Additionally, At 24 h post-injection, photothermal imaging were conducted on the mice with continuous laser irradiation (808 nm, 0.8 W cm^{-2}, 10 minutes). The infrared thermal images were monitored by the E6 IR camera.

In vivo phototherapeutic study

The MB49 tumor- bearing mice were randomly allocated into the following four groups (n = 5 per group) named "PBS," "PBS + L," "OTTITQ NPs," and "OTTITQ NPs + L." On day 0, all mice were injected intravenously via the tail vein with either PBS or OTTITQ NPs (10 mg/kg). The "PBS" and "OTTITQ NPs" groups without further laser irradiation served as control groups. In contrast, 24 h after the injection, the "PBS + L" and "OTTITQ NPs + L" groups were subjected to laser irradiation (808 nm, 0.8 W cm^{-2}, 10 minutes). Throughout the 15-day experimental period, the body weights of the mice were monitored every three days, and tumor size were assessed using a vernier caliper. Tumor volume was calculated using the formula $V = a \times b^2/2$, (a: tumor length; b: tumor width). The relative tumor volume (RTV) was determined by the formula $RTV = V/V_0$, with V_0 being the initial tumor volume.

Hemolysis assay

Whole blood (0.5 mL) was extracted from C57BL/6J mice by enucleation of the eyes. Following a centrifugation step at 3000 rpm for 10 minutes at 4 ℃, the red blood cells (RBCs) were harvested. The RBCs were washed three times using PBS and diluted to 10 mL. Then 0.2 mL of this RBC suspension were mixed with 0.8 mL of OTTITQ NPs to achieve final concentrations of 0, 5, 10, 20, 40, 60, 80, 100, and

150 μM in PBS (pH 7.4) for triplicate. The mixtures were then incubated with gentle shake at 50 rpm for 2 h at room temperature. Next, the samples were centrifuged at 3000 rpm for 5 minutes at 4 ℃, and supernatant absorbance was measured at 541 nm to determine hemoglobin content. The mixture of 0.2 mL RBC suspension and 0.8 mL PBS was recommended as a negative control (0% hemolysis), and 0.2 mL RBC suspension mixed with 0.8 mL ddH_2O was used as a positive control (100% hemolysis). The percentage of hemolysis for each sample was calculated using the formula: Hemolysis (%) = $(A_{sample}-A_{0\%})/(A_{100\%}-A_{0\%}) \times 100\%$.

Histological and Hematological Analyses

After termination of treatment on day 15, blood samples of all the mice were harvested for subsequent serum biochemistry and hematological assessments. After that, tumors and major organs (heart, liver, spleen, lungs, and kidneys) were excised from all the experimental groups. These tissues were then immersed in a 4% formaldehyde solution for fixation overnight, subsequently embedded in paraffin, and sectioned at a thickness of 5 mm. The tumor sections from the four groups underwent hematoxylin and eosin (H&E), CD31, Ki67, and TUNEL staining to assess the antitumor efficiency. Meanwhile, major organs sections were examined by H&E staining to evaluate the biosafety analysis.

2. Calculation, Synthesis and Characterization

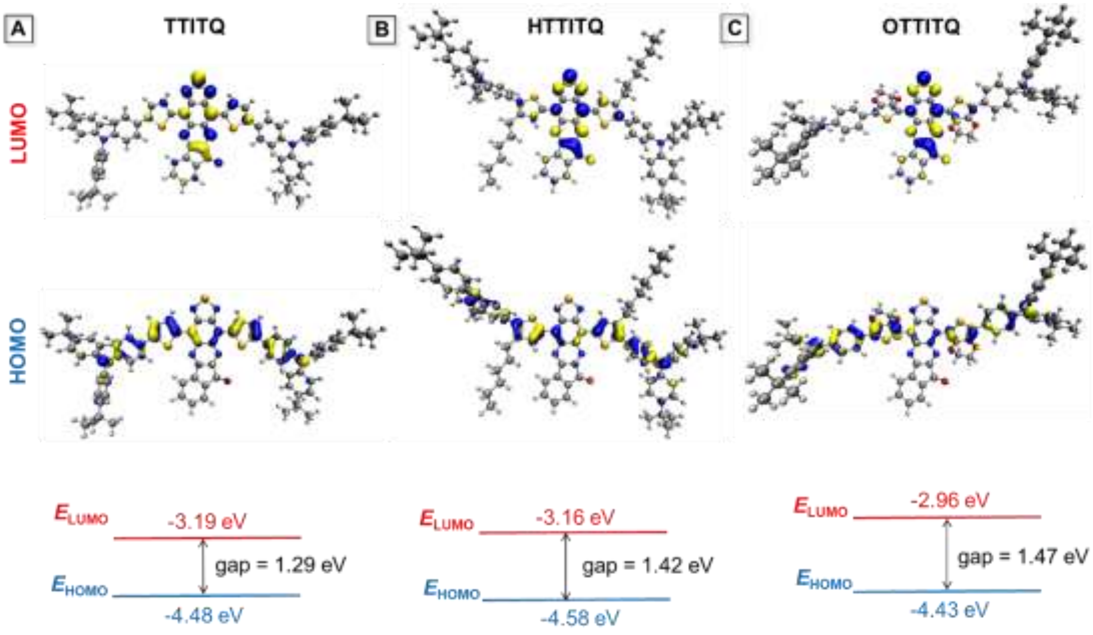

Figure S1. Illustration of the frontier molecular orbitals (LUMOs and HOMOs) of (A) TTITQ, (B) HTTITQ, (C) OTTITQ determined at the B3LYP/6-31 g (d) level of theory.

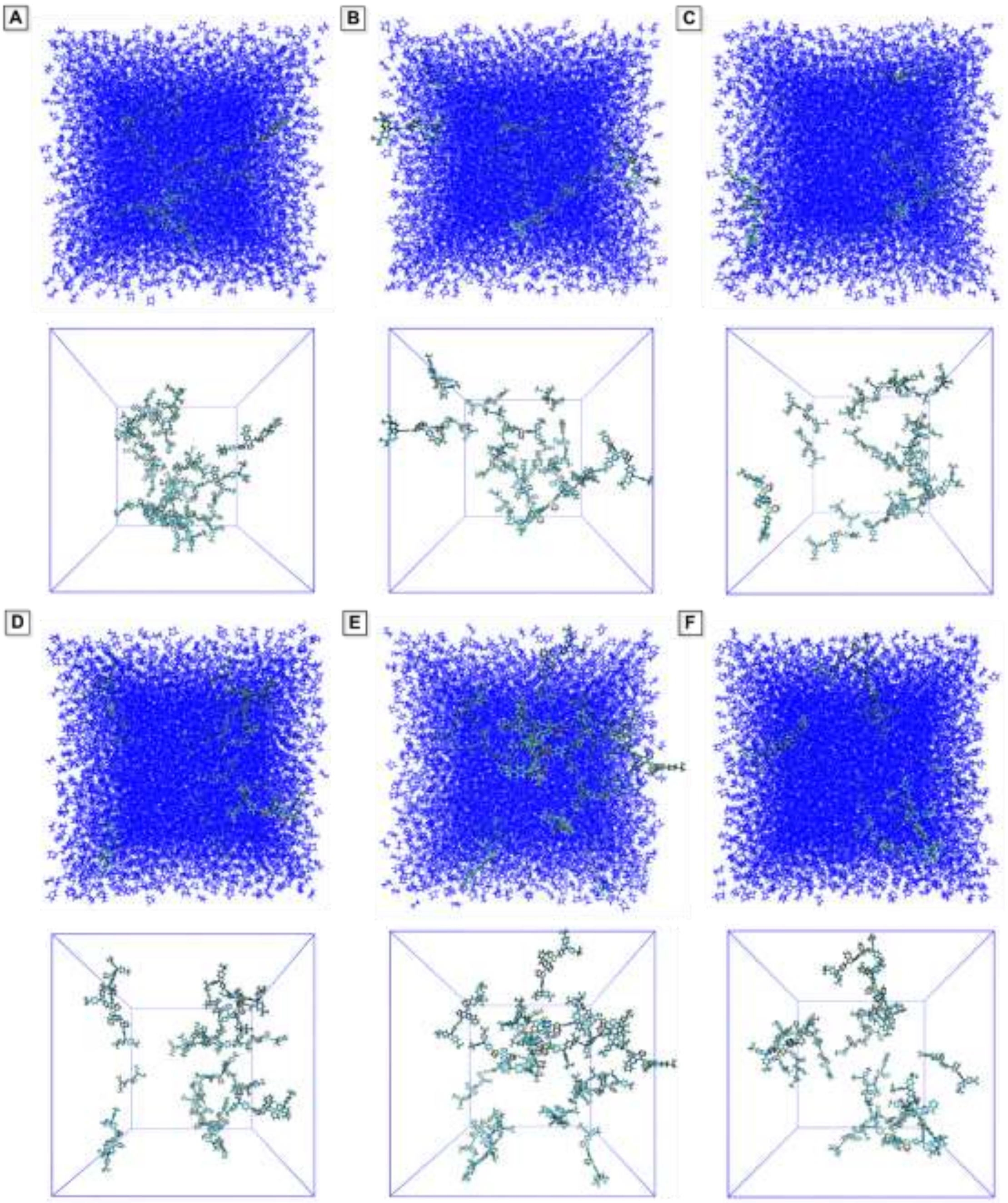

Figure S2. Throughout the course of the molecular dynamics simulations, the graphical representation illustrates the progression of OTTITQ at (A) 0 ns, (B) 20 ns, (C) 40 ns, (D) 60 ns, (E) 80 ns, (F) 100 ns in THF.

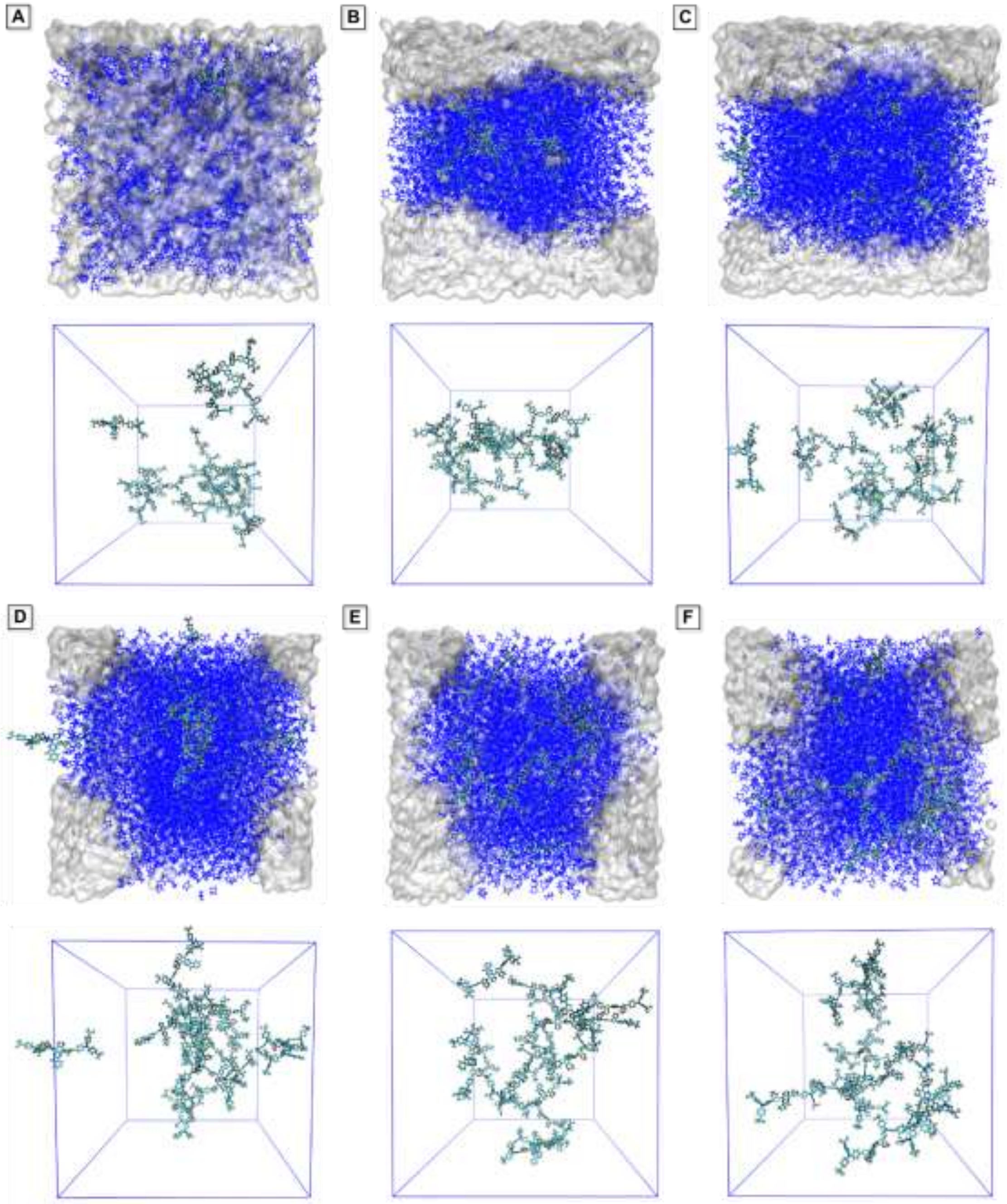

Figure S3. Throughout the course of the molecular dynamics simulations, the graphical representation illustrates the progression of OTTITQ at (A) 0 ns, (B) 20 ns, (C) 40 ns, (D) 60 ns, (E) 80 ns, (F) 100 ns in (V_{THF}/V_{water}) = 70%/30% .

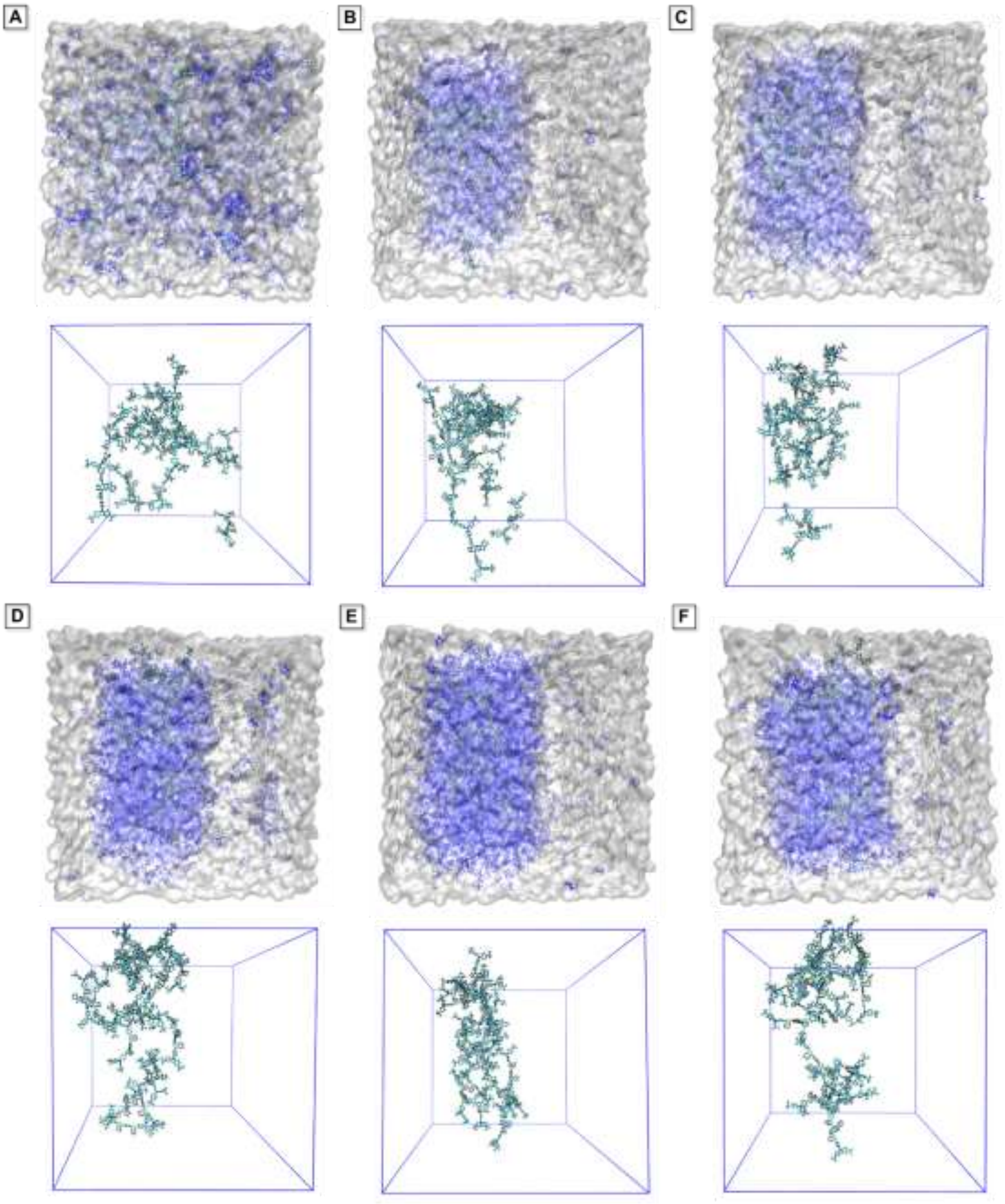

Figure S4. Throughout the course of the molecular dynamics simulations, the graphical representation illustrates the progression of OTTITQ at (A) 0 ns, (B) 20 ns, (C) 40 ns, (D) 60 ns, (E) 80 ns, (F) 100 ns in (V_{THF}/V_{water}) = 30%/70%.

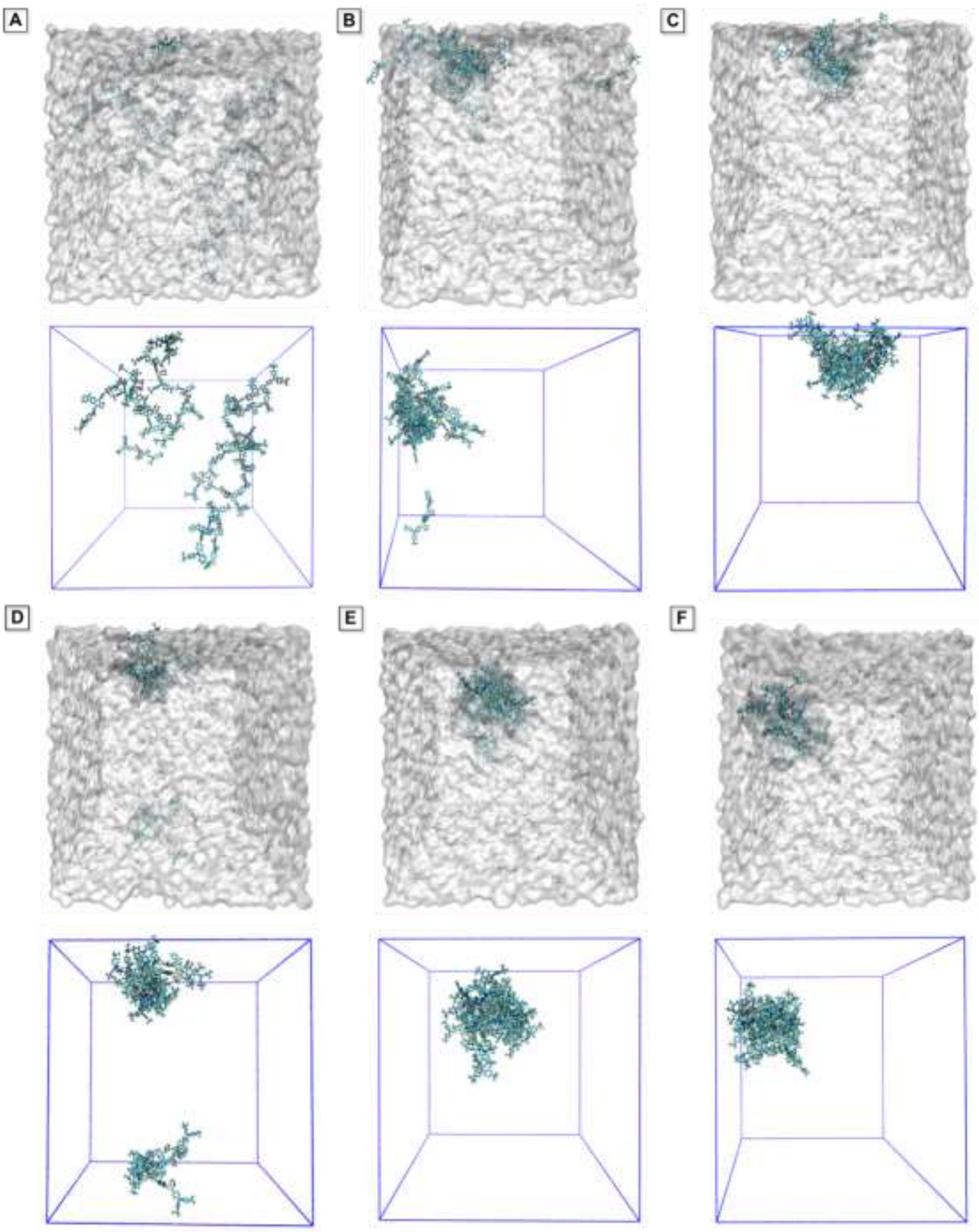

Figure S5. Throughout the course of the molecular dynamics simulations, the graphical representation illustrates the progression of OTTITQ at (A) 0 ns, (B) 20 ns, (C) 40 ns, (D) 60 ns, (E) 80 ns, (F) 100 ns in water.

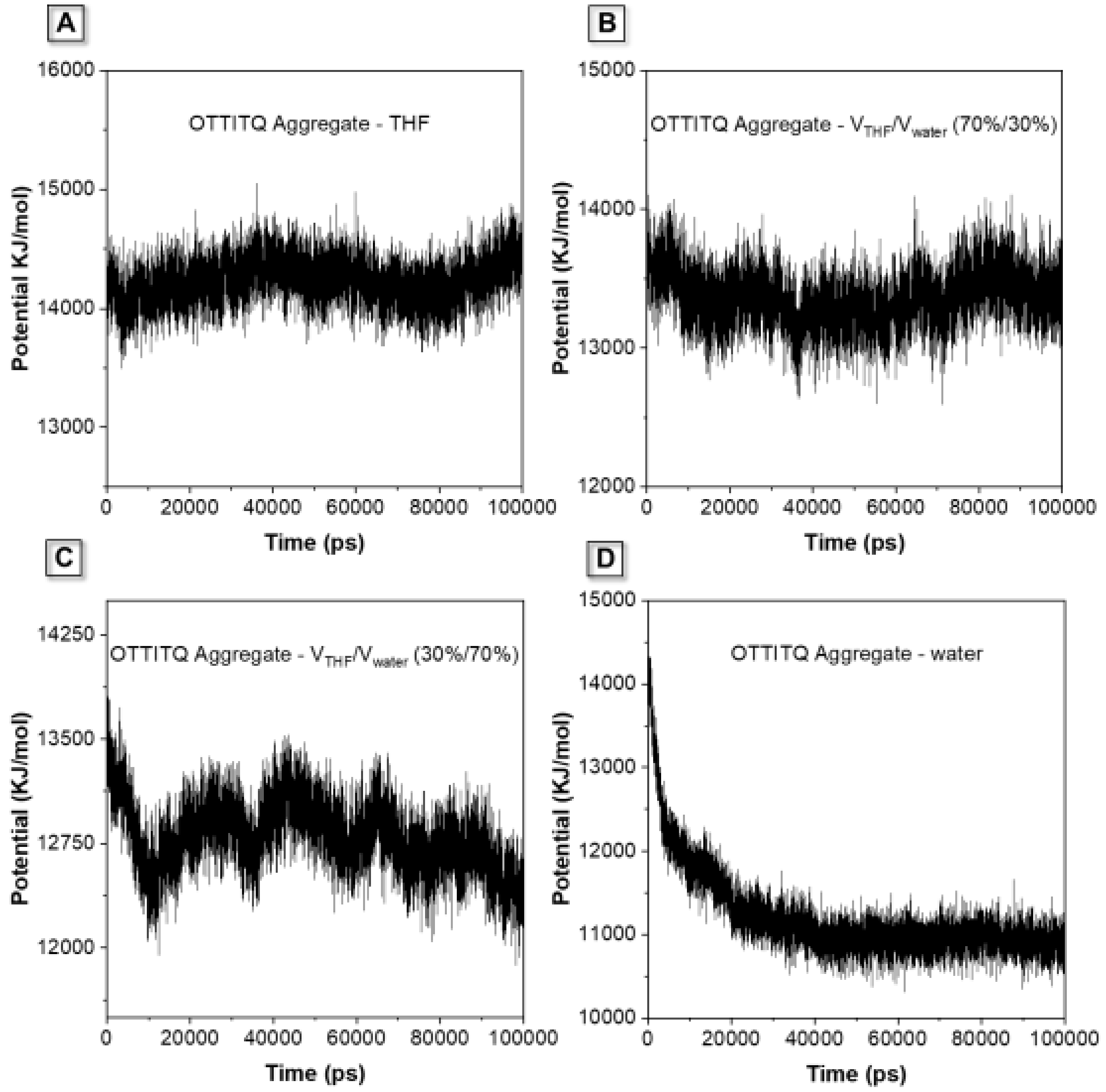

Figure S6. In the dynamics simulation, the variation of potential energy change with time for (A) OTTITQ aggregate in THF, (B) OTTITQ aggregate in (V_{THF}/V_{water}) = 70%/30%, (C) OTTITQ aggregate in (V_{THF}/V_{water}) = 30%/70%, (D) OTTITQ aggregate in water. Unit: KJ/mol.

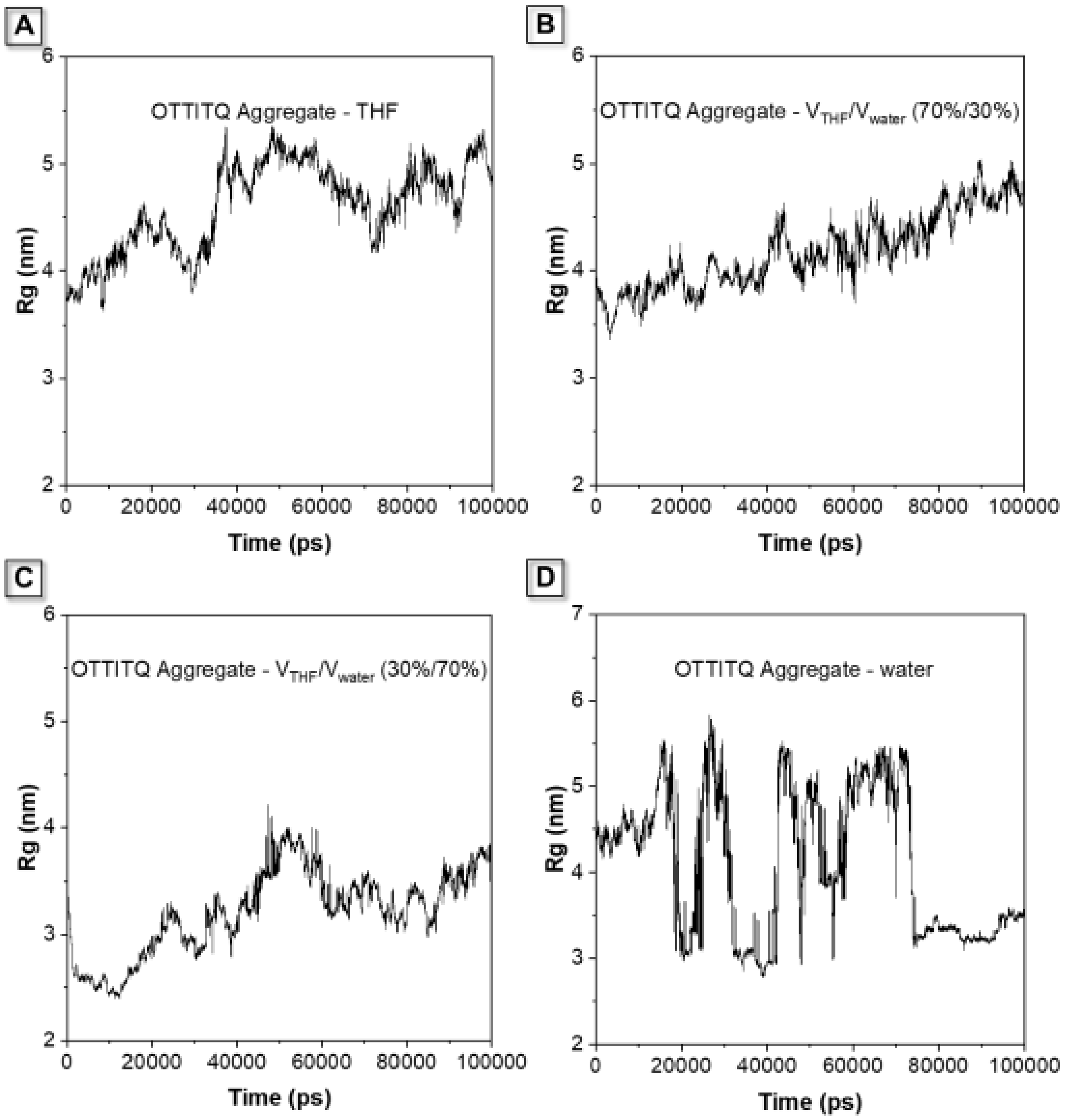

Figure S7. In the dynamics simulation, the radius of gyration of OTTITQ aggregate in (A) THF, (B) (V_{THF}/V_{water}) = 70%/30%, (C) (V_{THF}/V_{water}) = 30%/70%, (D) water.

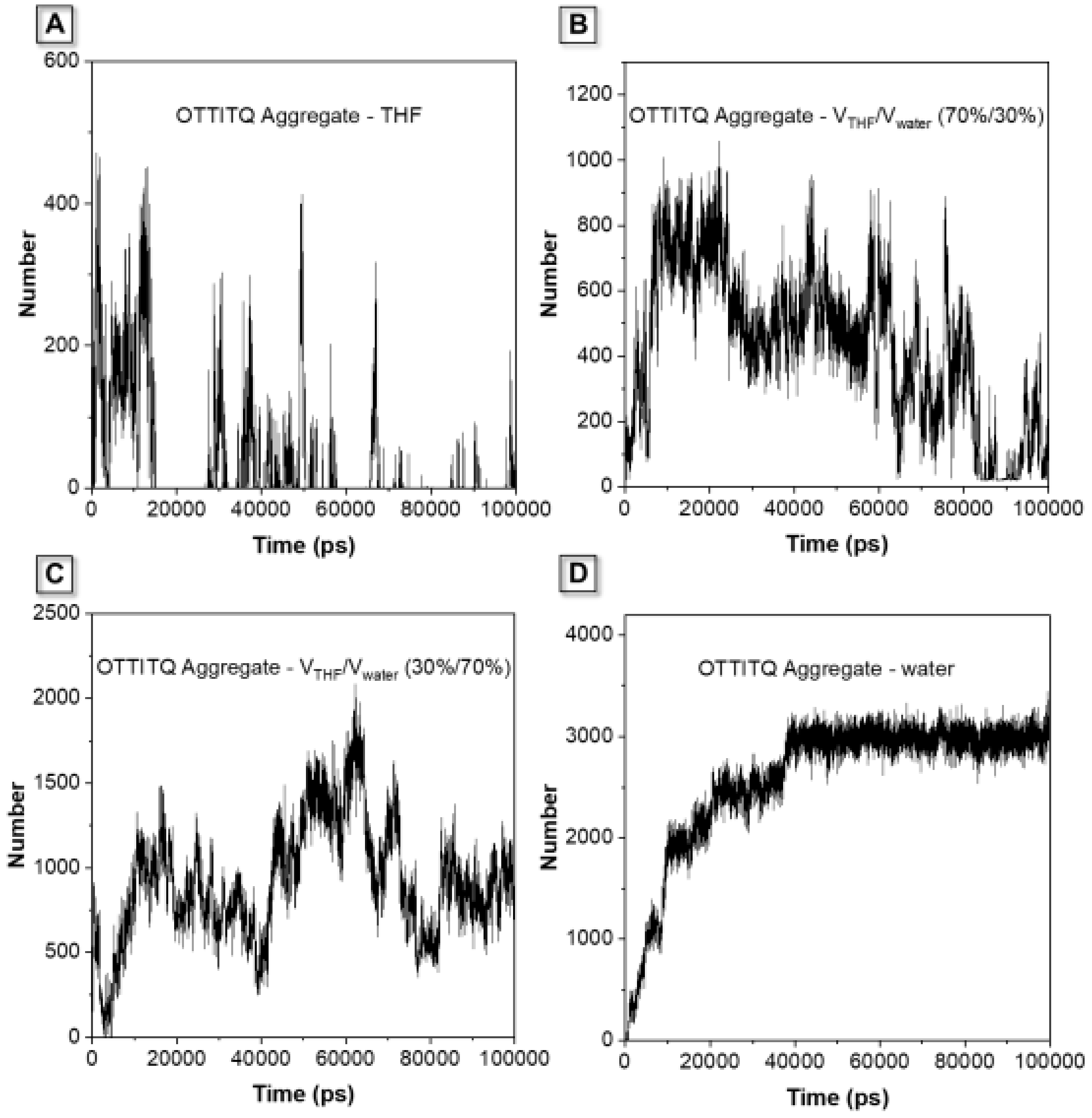

Figure S8. In the dynamics simulation, the number of atomic contacts between the innermost of OTTITQ aggregate and the outer of OTTITQ aggregate in (A) THF, (B) (V_{THF}/V_{water}) = 70%/30%, (C) (V_{THF}/V_{water}) = 30%/70%, (D) water.

Table S1. In the dynamics simulation, energy term of the average interaction energy under 100 ns for OTTITQ aggregate in THF, (V_{THF}/V_{water}) = 70%/30%, (V_{THF}/V_{water}) = 30%/70% and water. Unit: KJ/mol.

Energy term	in THF Interaction energy	in THF The proportion of interaction energy	in (V_{THF}/V_{water}) = 70%/30% Interaction energy	in (V_{THF}/V_{water}) = 70%/30% The proportion of interaction energy	in (V_{THF}/V_{water}) = 30%/70% Interaction energy	in (V_{THF}/V_{water}) = 30%/70% The proportion of interaction energy	in water Interaction energy	in water The proportion of interaction energy
$E_{vdw\text{-}SR}$	-4.3575	44.88%	-71.4545	76.25%	-160.7486	77.08%	-454.8041	82.92%
E_{disper}	-4.4622823	45.96%	-4.2502384	4.54%	-4.5383411	2.18%	-4.849116	0.88%
$E_{coulomb\text{-}SR}$	-0.1752	1.80%	-7.4598	7.96%	-12.2977	5.90%	-37.1097	6.77%
$E_{coul\text{-}recip}$	-0.7133	7.35%	-10.5478	11.26%	-30.9588	14.85%	-51.6989	9.43%
E_{total}	-9.7082823		-93.7123384		-208.5434411		-548.461816	

General procedure for the synthesis of TTITQ, HTTITQ and OTTITQ

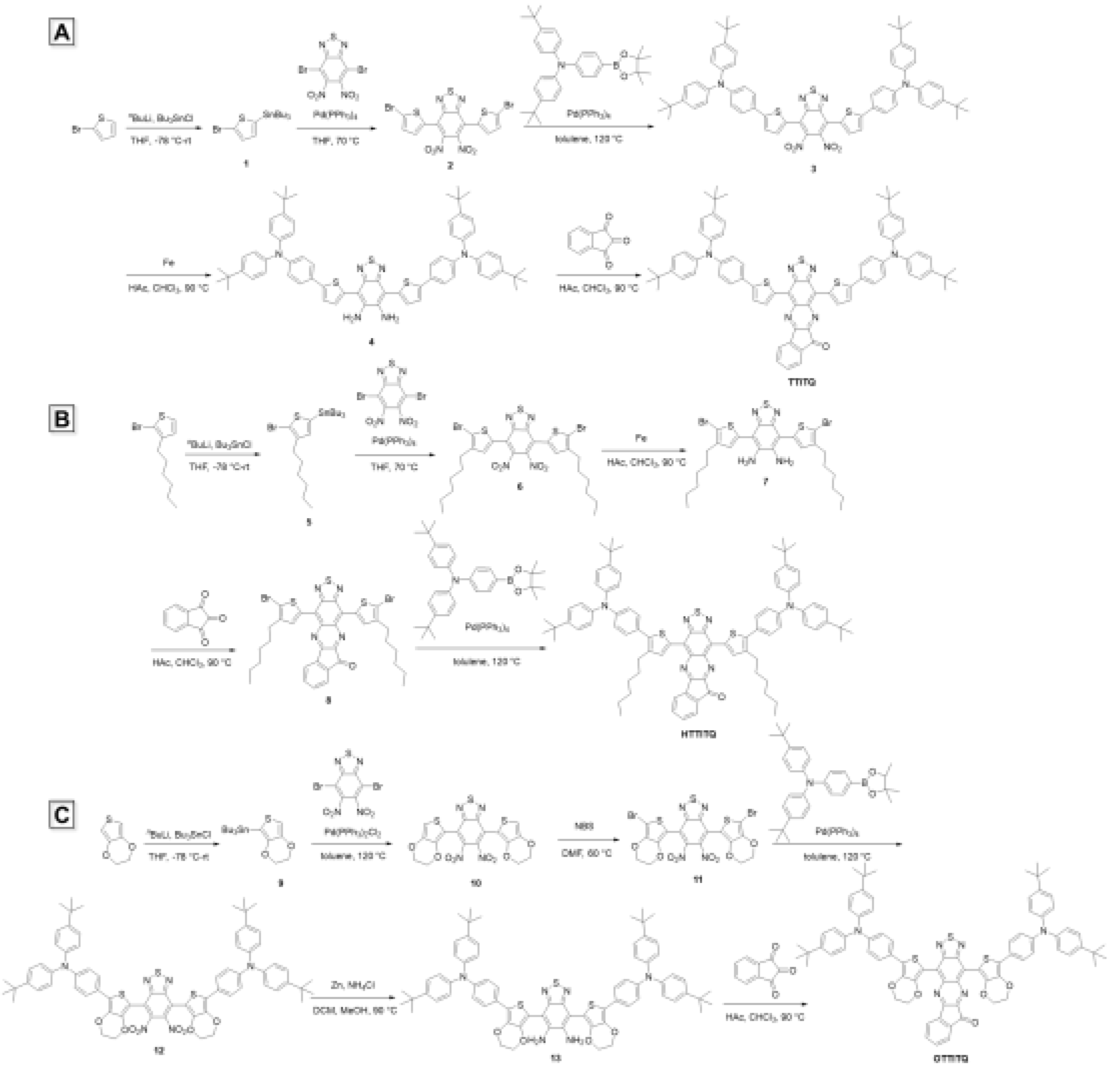

Scheme S1. The structures and synthetic routes of (A) TTITQ, (B) HTTITQ and (C) OTTITQ.

Synthesis of 1:[10]

Add ^{n}BuLi (3.6 mL, 9 mmol, 2.5 M in hexane) dropwise to a solution of 2-bromothiophene (580 μL, 6 mmol) in THF (10 mL) at -78 ℃. Stirring the reaction mixture 2 h at -78 ℃. Then tributyltin chloride (2.1 mL, 7.8 mmol) was added into the reaction at one portion. After stirring the mixture for 24 h at room temperature, KF solution was added to quench the reaction. The mixture was extracted with diethyl ether for three times, the combined organic phase was dried with Na_2SO_4. After

removing the solvent, the product was used directly without further purification.

Synthesis of 2:[11]

To a mixture of 1 (6 mmol), 4,7-dibromo-5,6-dinitrobenzo[c][1,2,5]thiadiazole (2.4 mmol, 921 mg), $Pd(PPh_3)_4$ (0.12 mmol, 138 mg) dissolved in dry THF (25 mL). The mixture was stirred at 70 ^oC for 17 h, then cool to room temperature. The mixture was extracted with DCM (50 mL × 3), the organic layers were combined, dried over Na_2SO_4, then evaporated under reduced pressure. The residue was subjected to column chromatography on silica gel (PE/DCM = 4:1~1:3) to give 2 as orange solid (818 mg, yield: 25%): ^1H NMR ($CDCl_3$, 400 MHz) δ (ppm) 7.26 (d, *J* = 4.0 Hz, 2H), 7.19 (d, *J* = 4.0 Hz, 2H).

Synthesis of 3:

To a mixture of 2 (0.4 mmol, 220 mg), 4-(*tert*-butyl)-*N*-(4-(*tert*-butyl)phenyl)-*N*-(4-(4,4,5,5-tetramethyl-1,3,2-dioxaborolan-2-yl)phenyl)aniline (0.8 mmol, 387 mg), $Pd(PPh_3)_4$ (0.04 mmol, 46 mg) and K_2CO_3 (2 mmol, 274 mg) in toluene (12 mL) and water (4 mL). The mixture was stirred at 120 ^oC for 24 h, then cool to room temperature. The mixture was extracted with DCM (50 mL × 3), the organic layers were combined, dried over Na_2SO_4, then evaporated under reduced pressure. The residue was subjected to column chromatography on silica gel (PE/DCM = 6:1~3:1) to give 3 as dark blue solid (103 mg, yield: 23%): ^1H NMR ($CDCl_3$, 600 MHz) δ (ppm) 7.56-7.53 (m, 6H), 7.35-7.33 (m, 10H), 7.12-7.09 (m, 12H), 1.37 (s, 36H). HRMS (MALDI-TOF) calculated for: $C_{66}H_{64}N_6O_4S_3$ $[M]^+$: 1100.415, found: 1100.439.

Synthesis of 4:

A mixture of compound 3 (44 mg, 0.04 mmol), Fe powder (68 mg, 1.2 mmol) in a mixture reaction solvents HAc/$CHCl_3$ (12.5 mL, v:v= 4:1) were stirred at 90 ˚C for 4 h. After cooling down to room temperature, the reaction mixture was washed by water and extracted with dichloromethane three times, dried over anhydrous Na_2SO_4. After removal of organic solvent under vacuum, the residual was directly used the next step.

Synthesis of TTITQ:

100 mL round bottom flask was charged with compound above prepared intermediate diamine, compound 4 (0.04 mmol) and 1*H*-indene-1,2,3-trione (0.04 mmol, 7 mg) dissolved in HAc/$CHCl_3$ (12.5 mL, v: v= 4:1), then the mixture was heated at 90 ˚C for 11 h. After cooling down to room temperature, the reaction mixture was washed by water and extracted with dichloromethane three times, dried over anhydrous Na_2SO_4. After removal of organic solvent under vacuum, the residue was subjected to column chromatography on silica gel (PE/DCM = 2:1~1:1) to give TTITQ as atrovirens solid (55 mg, yield: 99%): ^{1}H NMR ($CDCl_3$, 500 MHz) δ (ppm) 7.62-7.21 (m, 18H), 7.15-6.95 (m, 12H), 1.30 (s, 36H). ^{13}C NMR (150 MHz, $CDCl_3$) δ (ppm) 187.34, 148.19, 148.07, 146.45, 146.28, 144.80, 144.73, 140.25, 138.51, 138.31, 136.94, 135.91, 135.74, 134.43, 132.44, 132.37, 132.07, 131.87, 131.71, 128.68, 128.60, 127.31, 126.72, 126.64, 126.37, 126.31, 125.54, 125.19, 125.14, 124.59, 124.34, 123.78, 123.51, 122.23, 122.17, 120.10, 34.51, 31.66. HRMS (MALDI-TOF) calculated for: $C_{75}H_{68}N_6OS_3$ $[M]^+$: 1164.462, found: 1164.489.

Synthesis of 5: [12]

Add ^{n}BuLi (1.2 mL, 2.88 mmol, 2.5 M in hexane) dropwise to a solution of 2-bromo-3-hexylthiophene (2.4 g, 10 mmol) in dry THF (30 mL) at -78 ℃. Stirring the reaction mixture 2 h at -78 ℃. Then tributyltin chloride (3 mL, 10 mmol) was added into the reaction at one portion. And then the mixture was stirred at -78 ℃ for 18.5 hours. Then KF solution was added to quench the reaction, the mixture was extracted with diethyl ether for three times, the combined organic phase was dried with Na_2SO_4. After removing the solvent, the product was used directly without further purification.

Synthesis of 6:

To a mixture of 5 (10 mmol), 4,7-dibromo-5,6-dinitrobenzo[c][1,2,5]thiadiazole (4 mmol, 1.53 mg), $Pd(PPh_3)_4$ (0.8 mmol, 924 mg) dissolved in dry THF (40 mL). The mixture was stirred at 70 ^{o}C for 19.5 h, then cool to room temperature. The mixture was extracted with DCM (50 mL × 3), the organic layers were combined,

dried over Na_2SO_4, then evaporated under reduced pressure. The residue was subjected to column chromatography on silica gel (PE/DCM = 10:1~8:1) to give 6 as orange solid (891 mg, yield: 31%): ^{1}H NMR ($CDCl_3$, 500 MHz) δ (ppm) 7.19 (s, 1H), 7.10 (s, 1H), 2.55 (t, *J* = 7.5 Hz, 2H), 1.54-1.50 (m, 6H), 1.26-1.21 (m, 8H), 1.19 (s, 4H), 0.85-0.81 (m, 6H).

Synthesis of 7:

A mixture of compound 6 (465 mg, 0.65 mmol), Fe powder (1.12 g, 20 mmol) in a mixture reaction solvents HAc/$CHCl_3$ (25 mL, v: v= 4:1) were stirred at 90 ˚C for 4 h. After cooling down to room temperature, the reaction mixture was washed by water and extracted with dichloromethane three times, dried over anhydrous Na_2SO_4. After removal of organic solvent under vacuum, the residual was directly used the next step.

Synthesis of 8:

To a mixture of above prepared intermediate diamine, compound 7 (0.65 mmol) and 1*H*-indene-1,2,3-trione (0.65 mmol, 116 mg) dissolved in HAc/$CHCl_3$ (25 mL, v: v= 4:1), then the mixture was heated at 90 ˚C for 12 h. After cooling down to room temperature, the reaction mixture was washed by water and extracted with dichloromethane three times, dried over anhydrous Na_2SO_4. After removal of organic solvent under vacuum, the residue was subjected to column chromatography on silica gel (PE/DCM = 1:1~1:4) to give 8 as atrovirens solid (54 mg, yield: 11%): ^{1}H NMR ($CDCl_3$, 500 MHz) δ (ppm) 8.34 (s, 1H), 8.23 (s, 1H), 7.71 (d, *J* = 7.2 Hz, 1H), 7.624-7.619 (m, 2H), 7.54-7.52 (m, 1H), 2.49 (t, *J* = 7.8 Hz, 2H), 2.29 (t, *J* = 7.9 Hz, 2H), 1.65-1.59 (m, 2H), 1.42-1.28 (m, 14H), 0.92-0.88 (m, 6H). ^{13}C NMR ($CDCl_3$, 125 MHz) δ (ppm) 184.93, 150.79, 149.14, 148.34, 146.75, 140.19, 139.65, 138.31, 137.16, 134.91, 134.32, 133.48, 133.12, 132.73, 131.89, 131.41, 131.38, 122.82, 122.51, 120.61, 120.42, 117.56, 117.49, 30.74, 30.73, 28.66, 28.48, 28.36, 28.32, 28.30, 28.27, 21.83, 13.25. HRMS (MALDI-TOF) calculated for: $C_{35}H_{32}Br_2N_4OS_3$ $[M]^+$: 780.009, found: 780.051.

Synthesis of HTTITQ:

To a mixture of 8 (0.06 mmol, 47 mg),

4-(*tert*-butyl)-*N*-(4-(*tert*-butyl)phenyl)-*N*-(4-(4,4,5,5-tetramethyl-1,3,2-dioxaborolan-2-yl)phenyl)aniline (0.12 mmol, 58 mg), $Pd(PPh_3)_4$ (0.006 mmol, 7 mg) and K_2CO_3 (0.3 mmol, 42 mg) in toluene (9 mL) and water (3 mL). The mixture was stirred at 120 ^oC for 12 h, then cool to room temperature. The mixture was extracted with DCM (50 mL × 3), the organic layers were combined, dried over Na_2SO_4, then evaporated under reduced pressure. The residue was subjected to column chromatography on silica gel (PE/DCM = 2:1~1:4) to give HTTITQ as atrovirens solid (56 mg, yield: 70%): ^1H NMR ($CDCl_3$, 500 MHz) δ (ppm) 8.10 (d, *J* = 7.5 Hz, 1H), 7.84 (d, *J* = 7.5 Hz, 1H), 7.23 (t, *J* = 7.5 Hz, 1H), 7.58-7.54 (m, 2H), 7.44-7.20 (m, 9H), 7.18-6.92 (m, 16H), 2.74-2.64 (m, 3H), 1.70 (t, *J* = 7.5 Hz, 3H), 1.48 (s, 5H), 1.43-1.36 (m, 5H), 1.30-1.19 (m, 40H), 0.85-0.81 (m, 6H). ^{13}C NMR (150 MHz, $CDCl_3$) δ (ppm) 186.82, 149.84, 145.15, 143.57, 141.91, 139.54, 138.13, 137.61, 136.99, 135.24, 134.68, 131.35, 129.86, 128.81, 128.54, 128.45, 127.97, 127.77, 127.66, 126.54, 125.12, 125.03, 123.54, 123.19, 123.13, 123.00, 122.79, 122.71, 122.67, 122.51, 122.48, 122.32, 121.97, 121.84, 121.18, 120.98, 119.89, 115.21, 33.33, 33.28, 31.04, 30.80, 30.79, 30.51, 30.48, 30.40, 28.68, 28.50, 28.47, 23.81, 21.75, 13.21. HRMS (MALDI-TOF) calculated for: $C_{87}H_{92}N_6OS_3$ $[M]^+$: 1332.650, found: 1332.703.

Synthesis of 9:[13]

Add ^{n}BuLi (1.3 mL, 3.25 mmol, 2.5 M in hexane) dropwise to a solution of 2,3-dihydrothieno[3,4-b][1,4]dioxine (400 mg, 2.8 mmol) in dry THF (30 mL) at -78 ℃. Stirring the reaction mixture 0.5 h at -78 ℃, the temperature rose to -40 ℃. Then tributyltin chloride (1 mL, 3.7 mmol) was dropwise added into the reaction system. After stirring the mixture for 0.5 h at -40 ℃, heat up to room temperature for 24 hours. Then KF solution was added to quench the reaction, the mixture was extracted with diethyl ether for three times, the combined organic phase was dried with Na_2SO_4. After removing the solvent, the product was used directly without further purification.

Synthesis of 10:[14]

To a mixture of 9 (2.8 mmol), 4,7-dibromo-5,6-dinitrobenzo[c][1,2,5]thiadiazole

(0.93 mmol, 357 mg), $Pd(PPh_3)_2Cl_2$ (0.28 mmol, 197 mg) dissolved in dry toluene (20 mL). The mixture was stirred at 120 ^oC for 14 h, then cool to room temperature. The mixture was extracted with DCM (50 mL × 3), the organic layers were combined, dried over Na_2SO_4, then evaporated under reduced pressure. The residue was subjected to column chromatography on silica gel (PE/DCM = 1:1~1:5) to give 10 as red solid (376 mg, yield: 80%): ^1H NMR ($CDCl_3$, 400 MHz) δ (ppm) 6.77 (s, 2H), 4.25-4.20 (m, 8H).

Synthesis of 11:[15]

To a mixture of 10 (0.2 mmol, 100 mg) and NBS (0.44 mmol, 87 mg) dissolved in DMF (5 mL). The mixture was stirred at 60 ^oC for 4 h, then cool to room temperature. The mixture was extracted with EA (50 mL × 3), the organic layers were combined, dried over Na_2SO_4, then evaporated under reduced pressure. The residue was subjected to column chromatography on silica gel (PE/DCM = 1:3~1:8) to give 11 as red solid (83 mg, yield: 63%): ^1H NMR ($CDCl_3$, 500 MHz) δ (ppm) 4.26-4.24 (m, 4H), 4.15-4.13 (m, 4H).

Synthesis of 12:

To a mixture of 11 (0.12 mmol, 80 mg), 4-(*tert*-butyl)-*N*-(4-(*tert*-butyl)phenyl)-*N*-(4-(4,4,5,5-tetramethyl-1,3,2-dioxaborolan-2-yl)phenyl)aniline (0.24 mmol, 116 mg), $Pd(PPh_3)_4$ (0.012 mmol, 14 mg) and K_2CO_3 (0.6 mmol, 83 mg) in toluene (12 mL) and water (4 mL). The mixture was stirred at 120 ^oC for 20 h, then cool to room temperature. The mixture was extracted with DCM (50 mL × 3), the organic layers were combined, dried over Na_2SO_4, then evaporated under reduced pressure. The residue was subjected to column chromatography on silica gel (PE/DCM = 1:1) to give 12 as dark blue solid (105 mg, yield: 72%): ^1H NMR ($CDCl_3$, 500 MHz) δ (ppm) 7.56 (d, J = 8.5 Hz, 4H), 7.21-7.19 (m, 8H), 6.99-6.97 (d, J = 8.5 Hz, 12H), 4.25-4.24 (m, 4H), 4.17-4.15 (m, 4H), 1.25 (s, 36H). ^{13}C NMR ($CDCl_3$, 125 MHz) δ (ppm) 152.66, 146.23, 144.57, 142.75, 136.79, 127.26, 126.13, 124.65, 124.41, 122.17, 119.42, 64.47, 34.34, 31.46. HRMS (MALDI-TOF) calculated for: $C_{70}H_{68}N_6O_8S_3$ $[M]^+$: 1216.426, found: 1216.452.

Synthesis of 13:

A mixture of compound 12 (18 mg, 0.015 mmol), Zn powder (118 mg, 1.8 mmol) and NH_4Cl (0.2 mmol, 11 mg) in a mixture reaction solvents DCM/MeOH (10 mL, v: v= 1:1) and 1 mL water were stirred at room temperature for 4 h. The reaction mixture was washed by water and extracted with dichloromethane three times, dried over anhydrous Na_2SO_4. After removal of organic solvent under vacuum, the residual was directly used the next step.

Synthesis of OTTITQ:

100 mL round bottom flask was charged with compound above prepared intermediate diamine, compound 13 (0.015 mmol) and 1*H*-indene-1,2,3-trione (0.015 mmol, 3 mg) dissolved in HAc/$CHCl_3$ (12.5 mL, v: v= 4:1), then the mixture was heated at 90 ˚C for 12 h. After cooling down to room temperature, the reaction mixture was washed by water and extracted with dichloromethane three times, dried over anhydrous Na_2SO_4. After removal of organic solvent under vacuum, the residue was subjected to column chromatography on silica gel (PE/DCM = 2:1~1:3) to give OTTITQ as atrovirens solid (11 mg, yield: 57%): ^{1}H NMR ($CDCl_3$, 600 MHz) δ (ppm) 8.24-8.22 (m, 2H), 8.03 (d, J = 7.8 Hz, 3H), 7.86-7.65 (m, 7H), 7.55-7.29 (m, 8H), 7.13-6.99 (m, 8H), 4.56-4.37 (m, 8H), 1.37 (s, 36H). ^{13}C NMR ($CDCl_3$, 150 MHz) δ (ppm) 188.86, 154.41, 154.15, 152.92, 150.14, 147.15, 146.99, 145.83, 145.81, 144.75, 144.73, 142.82, 141.84, 141.33, 138.85, 138.35, 138.04, 137.73, 137.31, 136.71, 132.90, 127.20, 127.05, 126.21, 126.11, 126.02, 125.73, 124.51, 124.16, 124.13, 123.45, 123.24, 122.53, 122.46, 121.93, 121.59, 107.23, 107.05, 64.64, 65.60, 64.40, 64.30, 34.27, 31.42. HRMS (MALDI-TOF) calculated for: $C_{79}H_{72}N_6O_5S_3$ $[M]^+$: 1280.473, found: 1280.560.

NMR and HRMS spectra of compounds

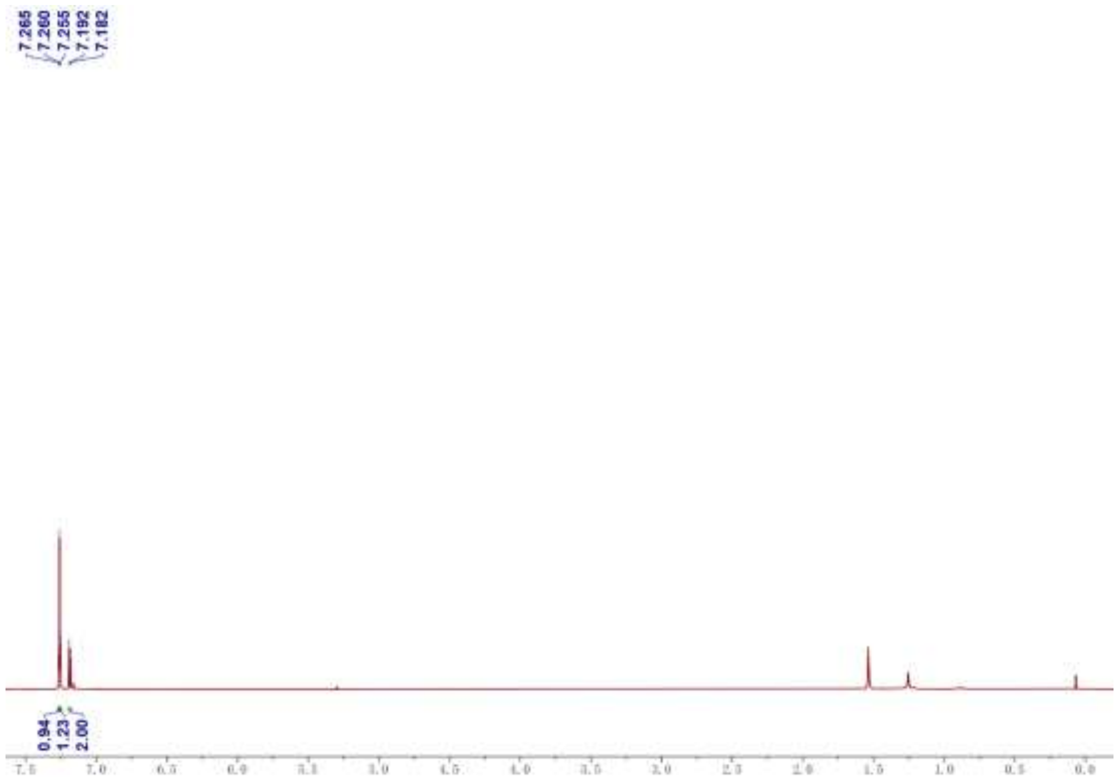

Figure S9. ^{1}H NMR spectrum of 2.

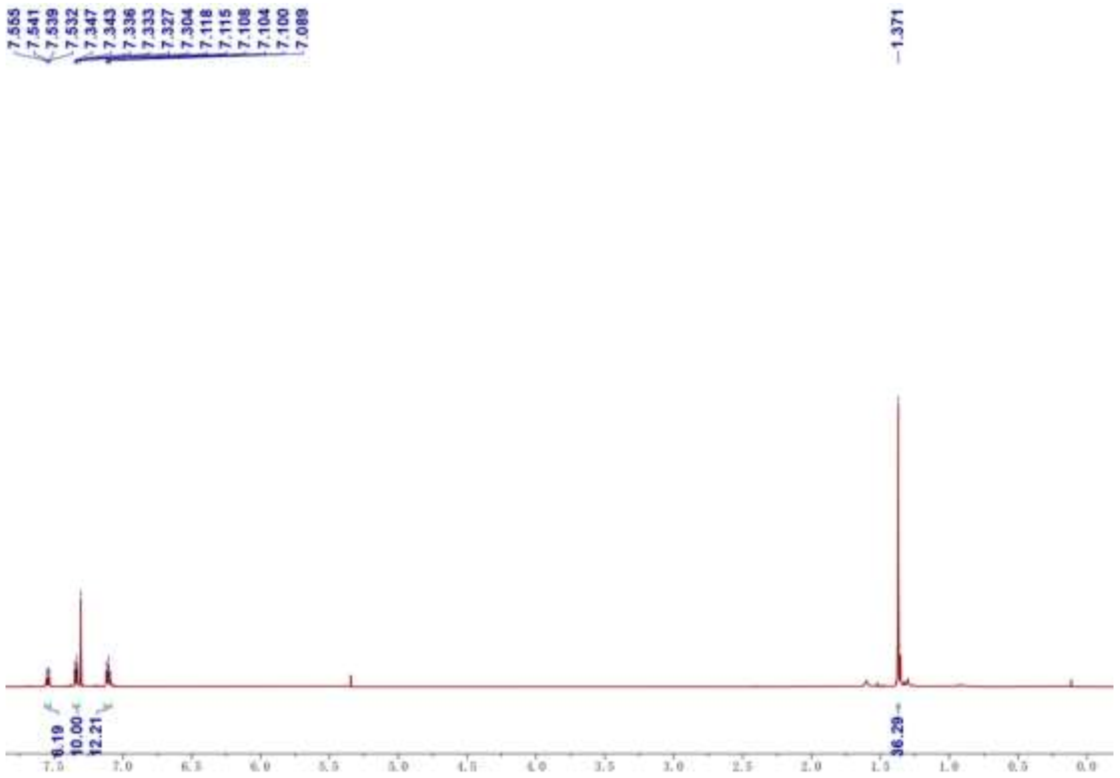

Figure S10. ^{1}H NMR spectrum of 3.

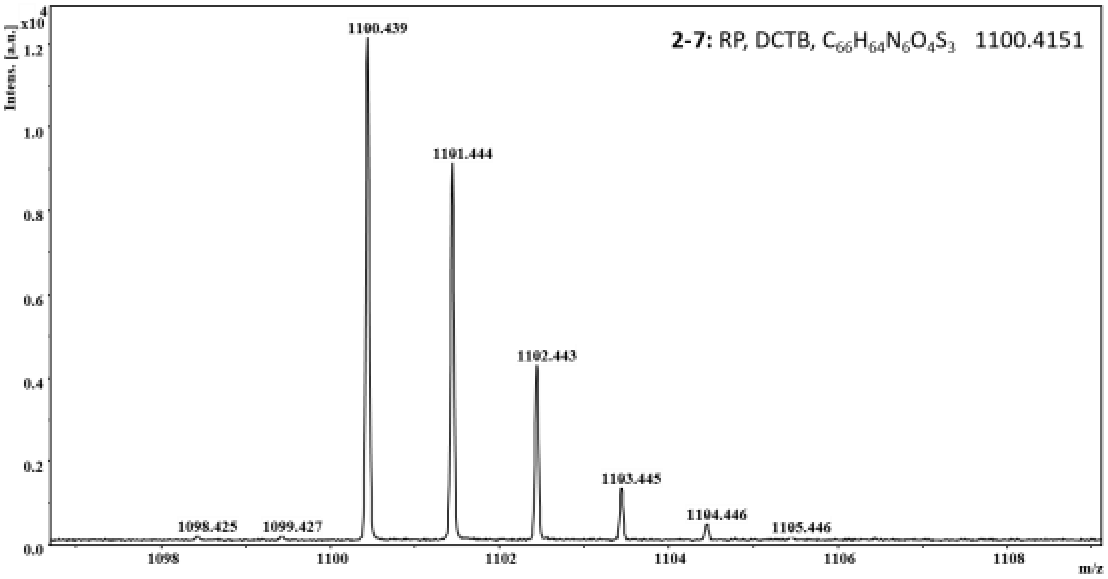

Figure S11. HRMS spectrum of 3.

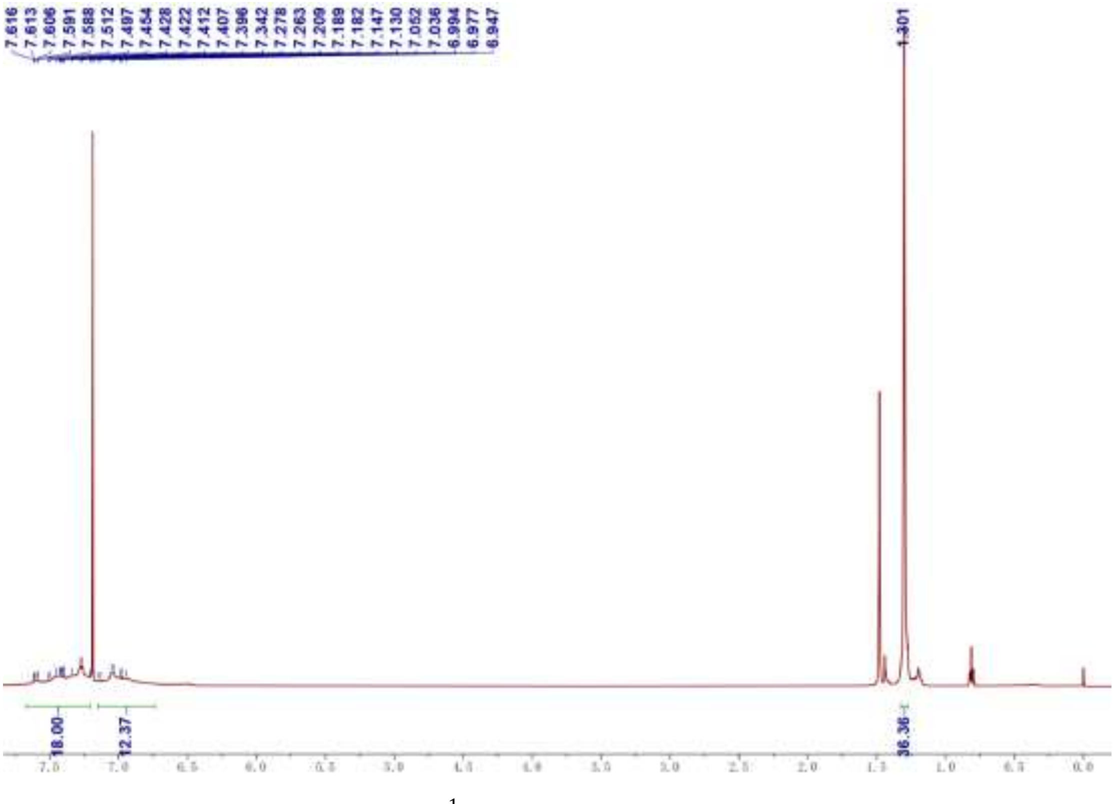

Figure S12. ^{1}H NMR spectrum of TTITQ.

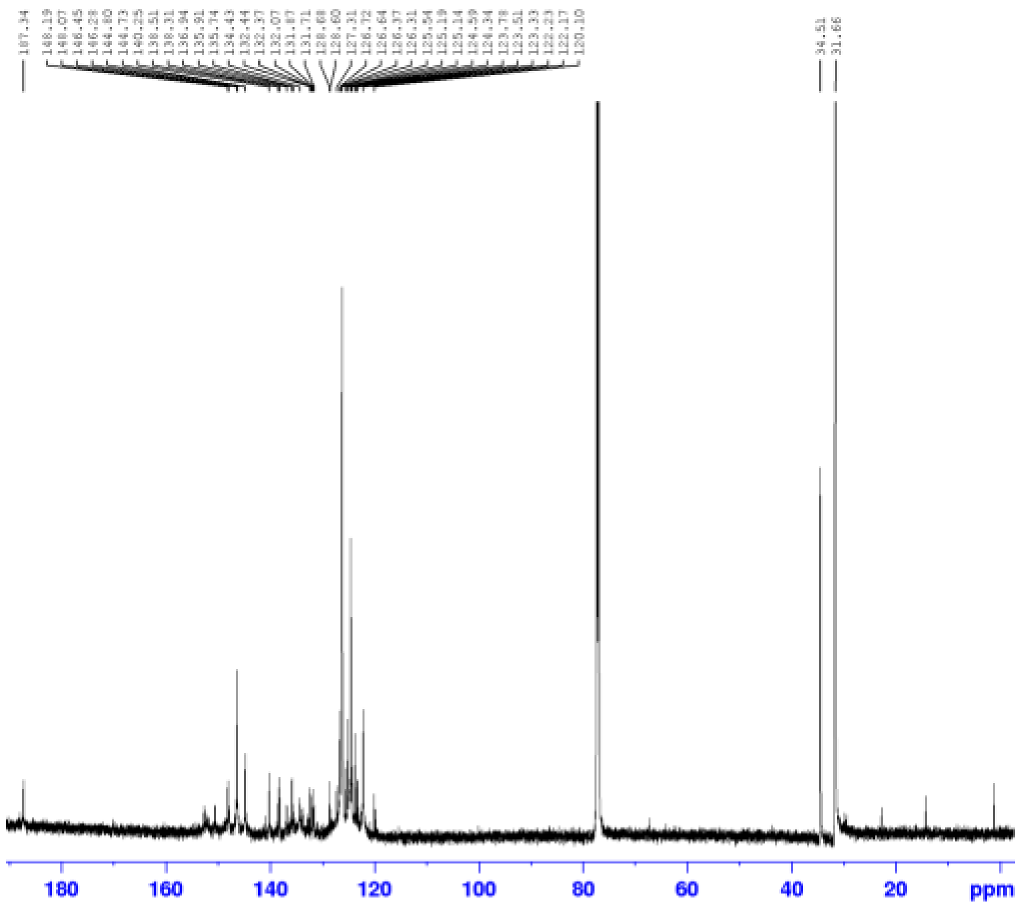

Figure S13. ^{13}C NMR spectrum of TTITQ.

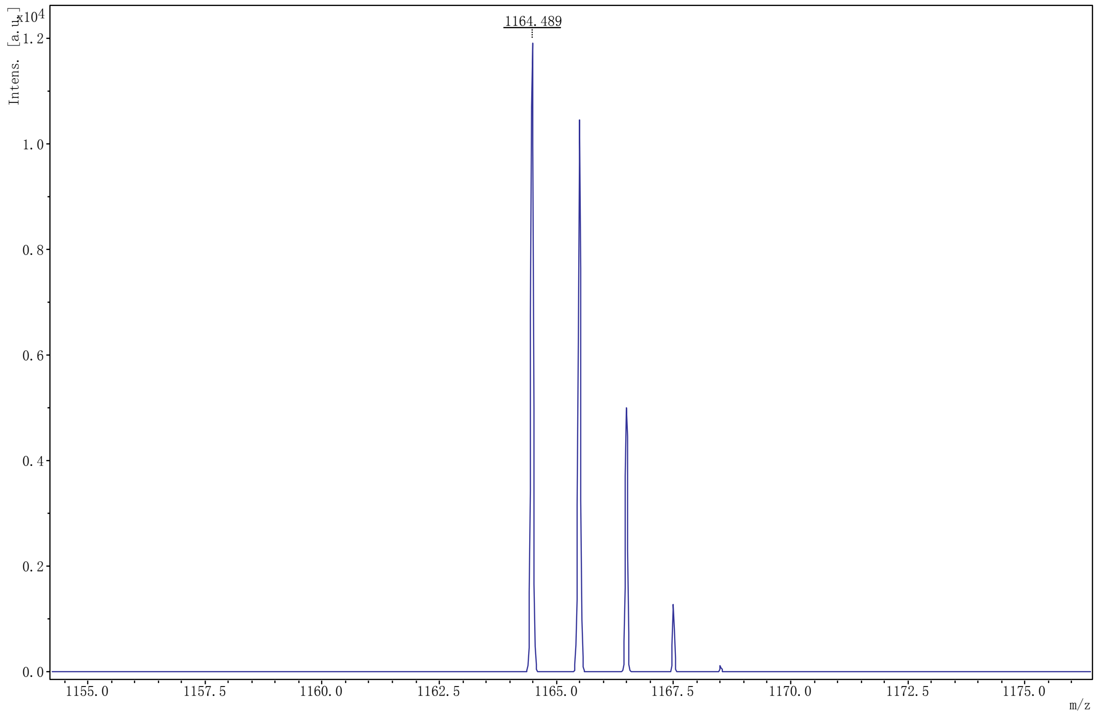

Figure S14. HRMS spectrum of TTITQ.

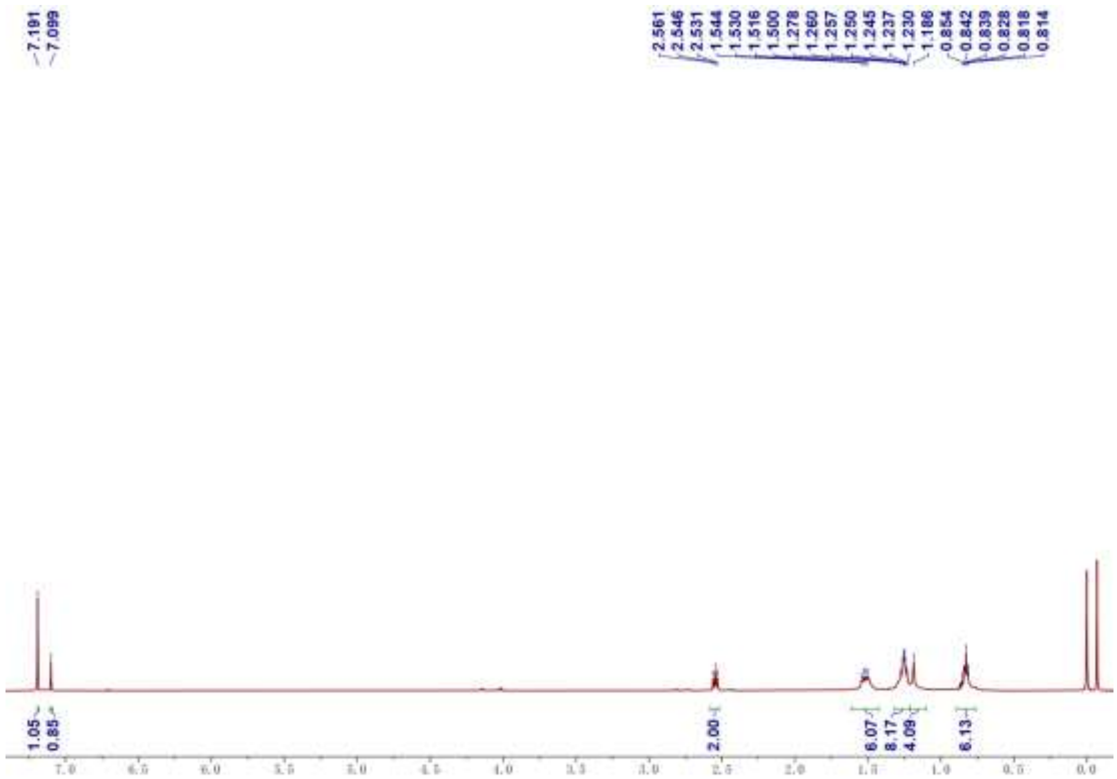

Figure S15. ^{1}H NMR spectrum of 6.

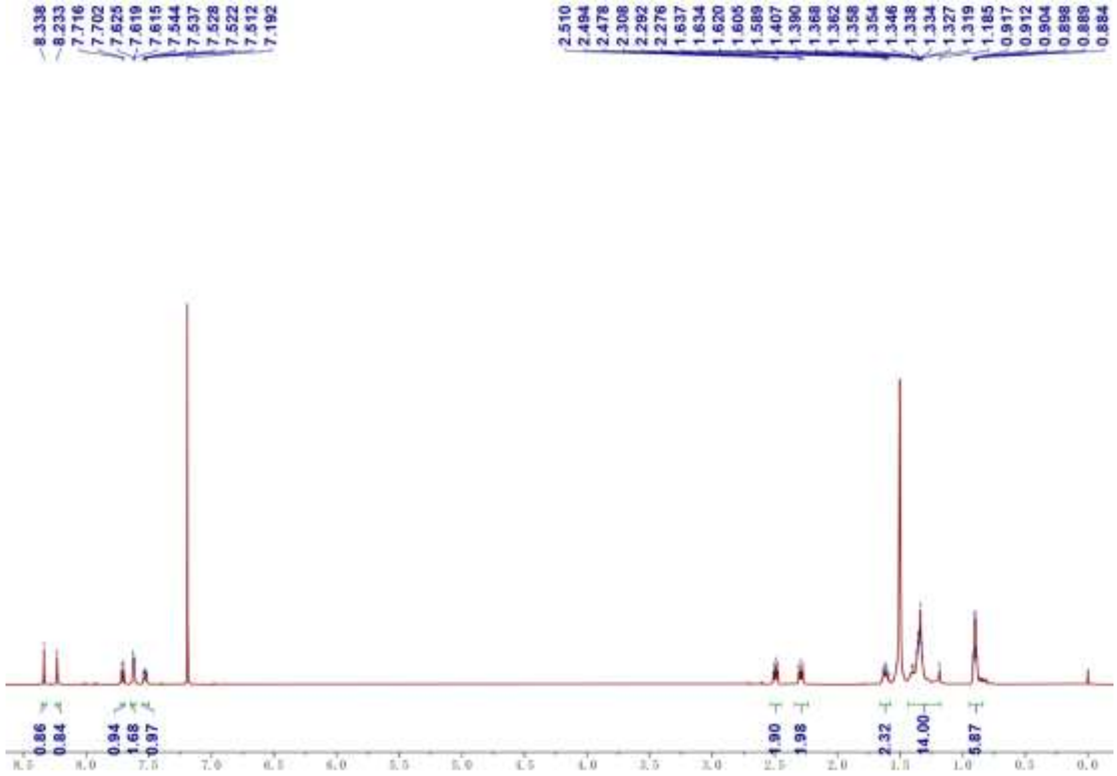

Figure S16. ^{1}H NMR spectrum of 8.

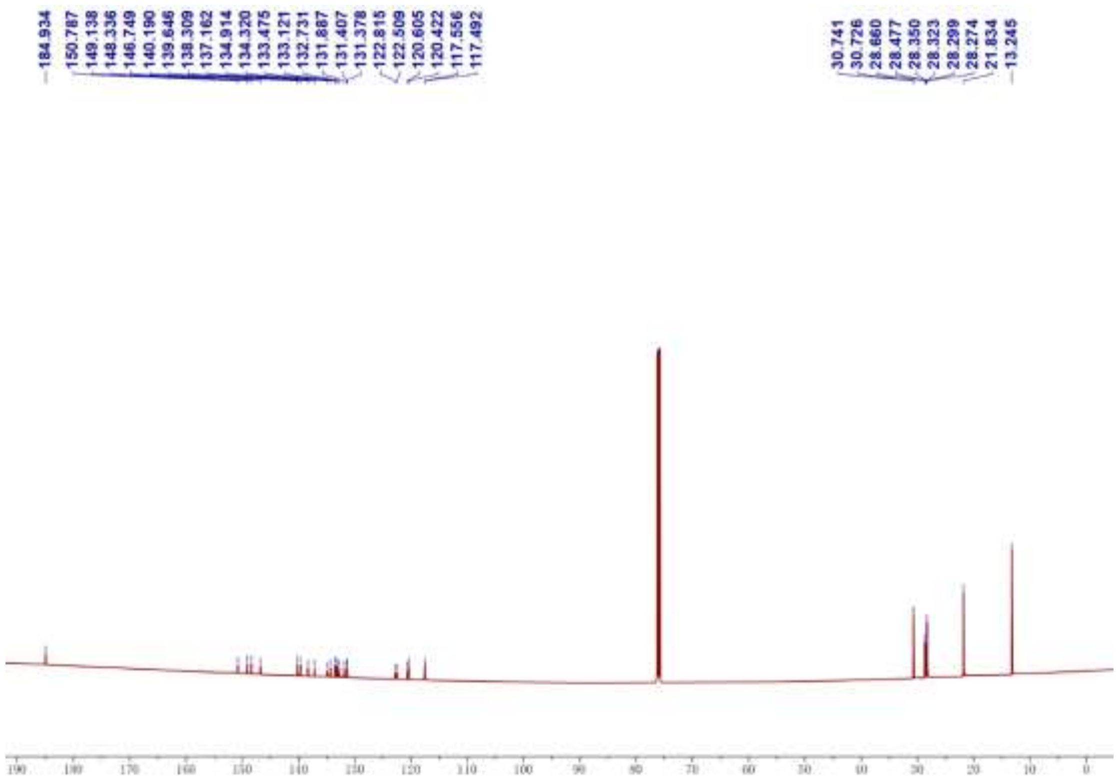

Figure S17. ^{13}C NMR spectrum of 8.

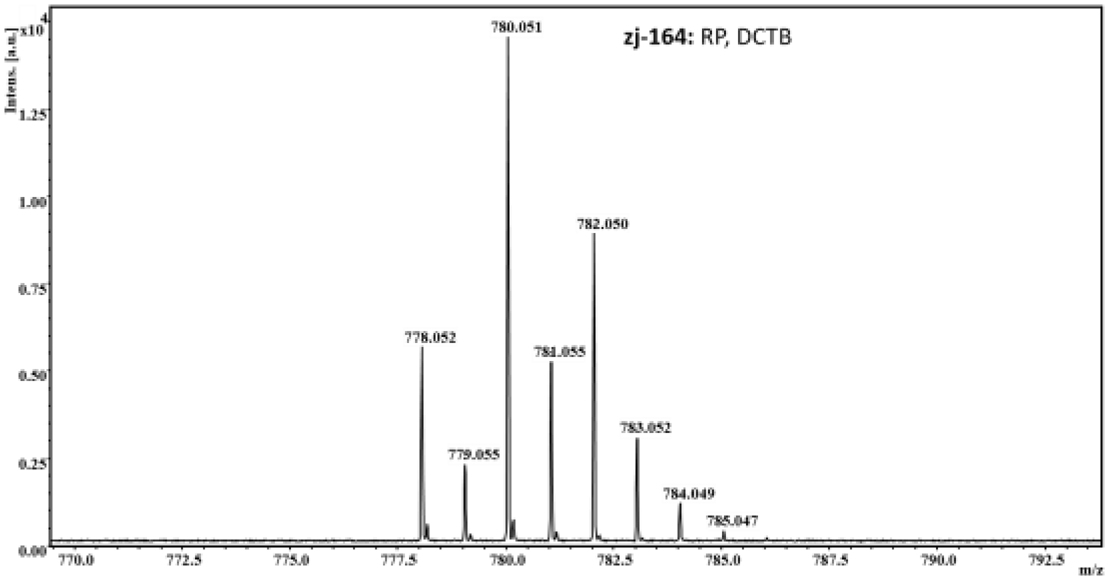

Figure S18. HRMS spectrum of 8.

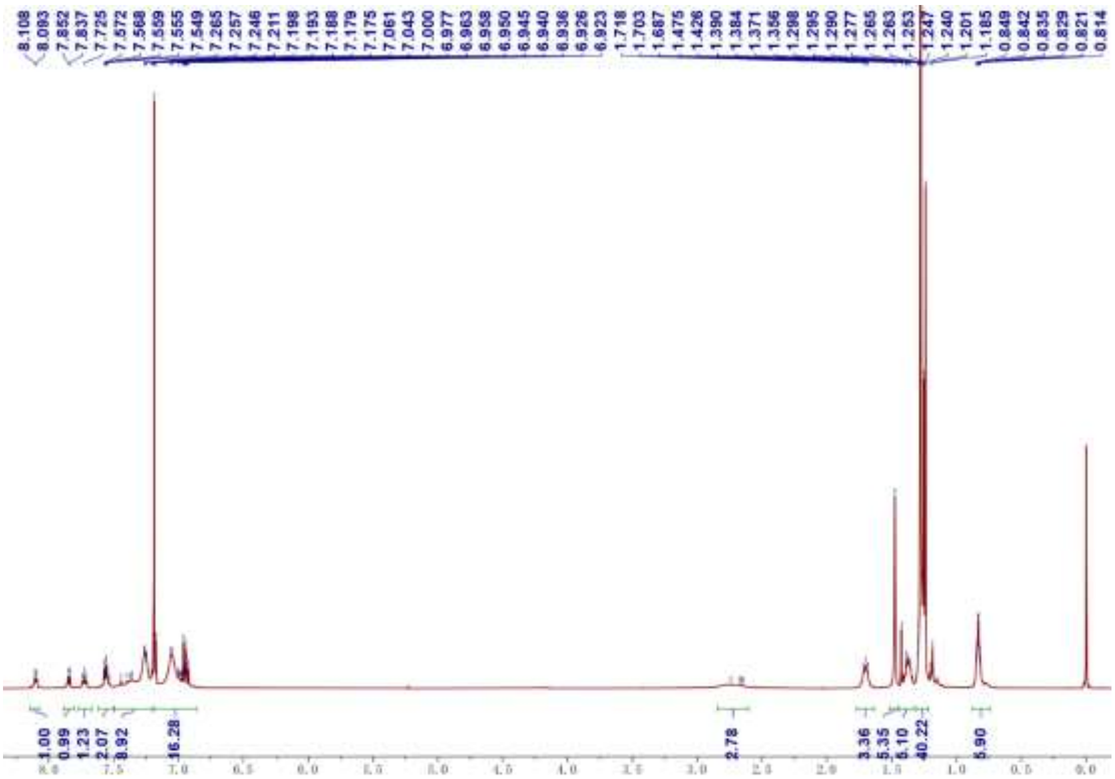

Figure S19. ^{1}H NMR spectrum of HTTITQ.

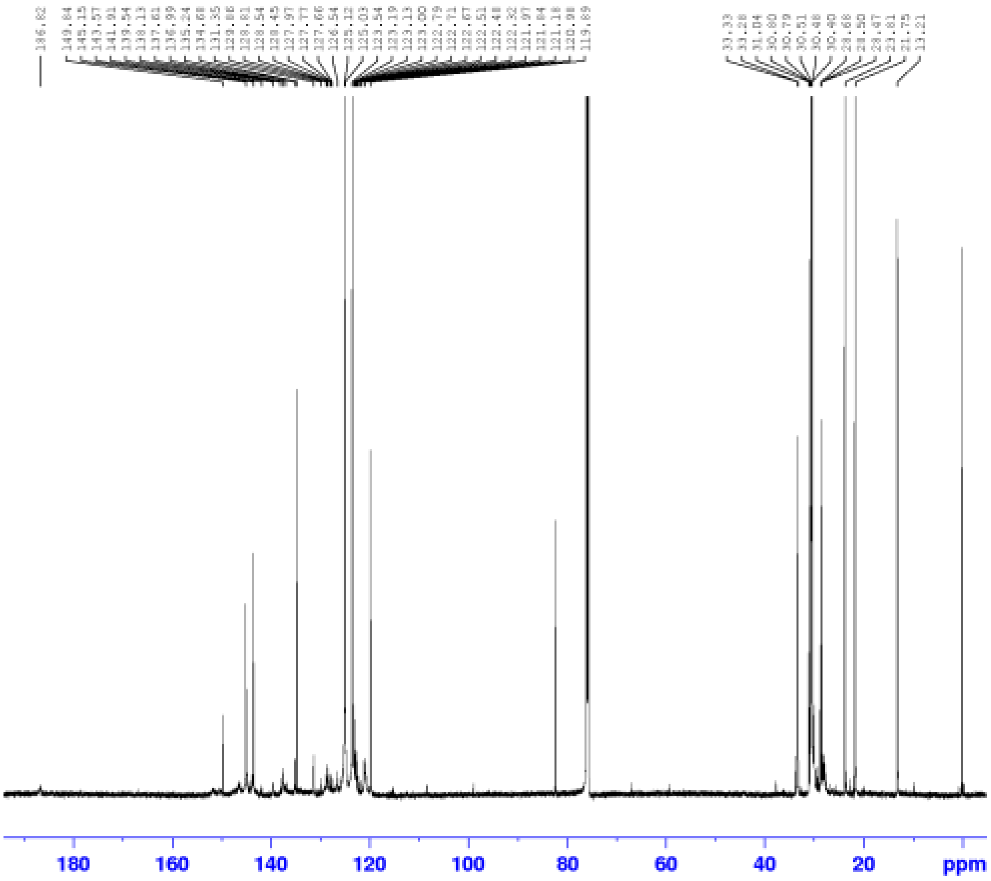

Figure S20. ^{13}C NMR spectrum of HTTITQ.

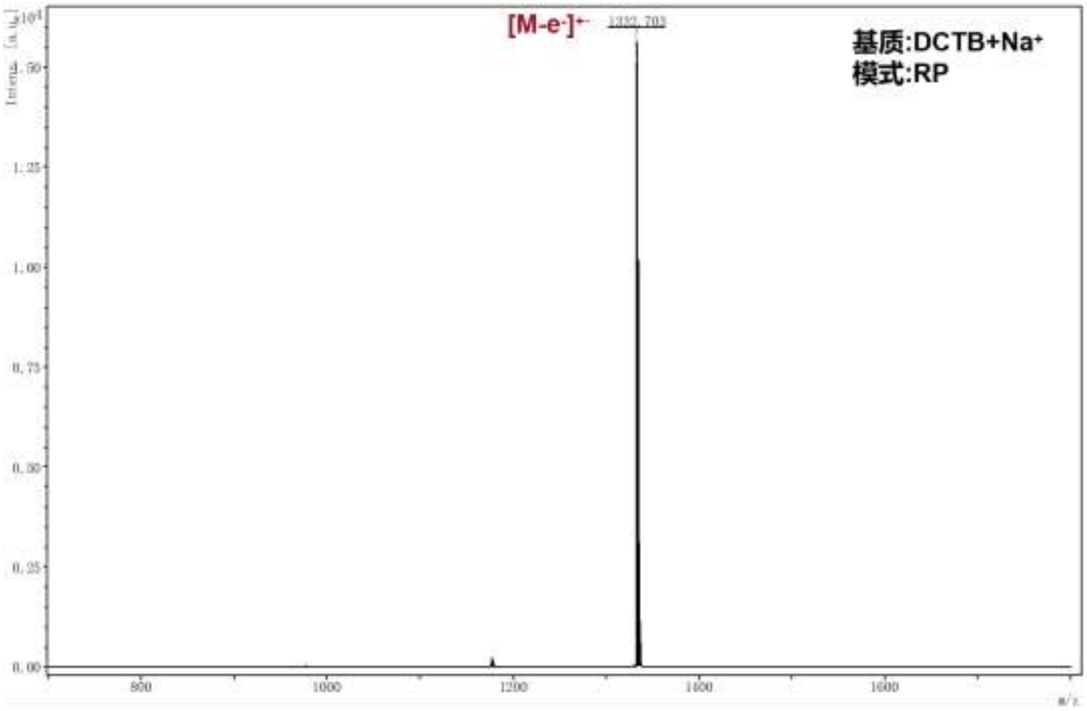

Figure S21. HRMS spectrum of HTTITQ.

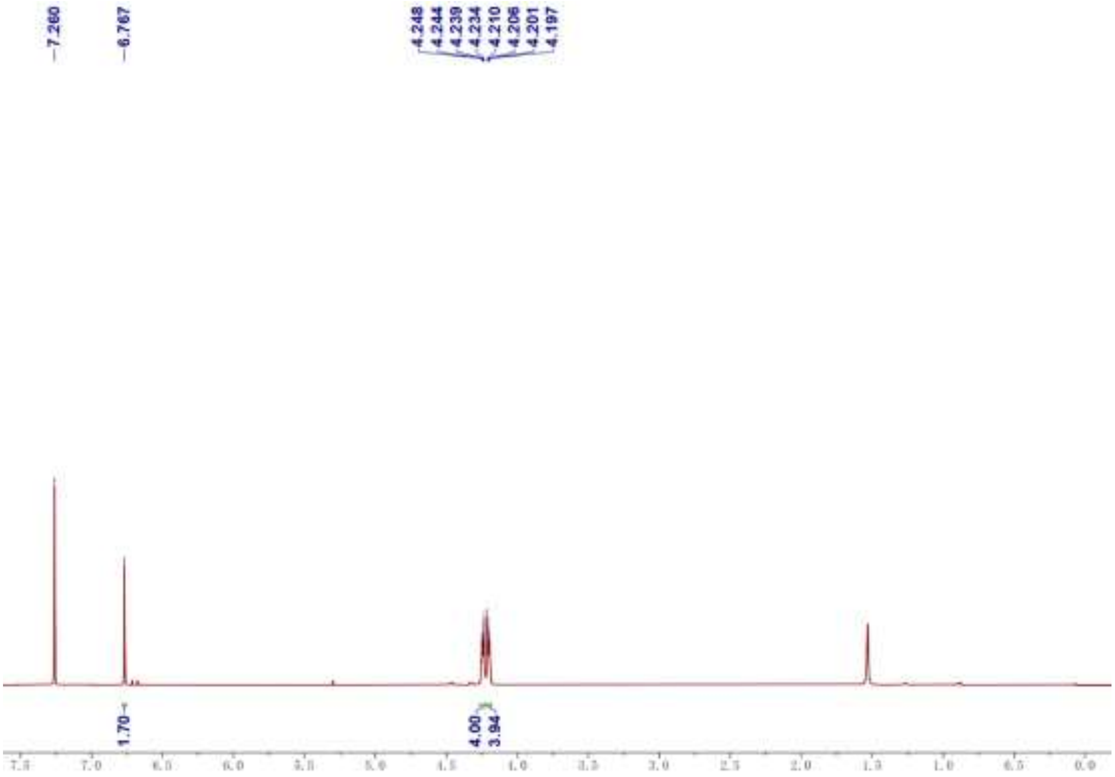

Figure S22. ^{1}H NMR spectrum of 10.

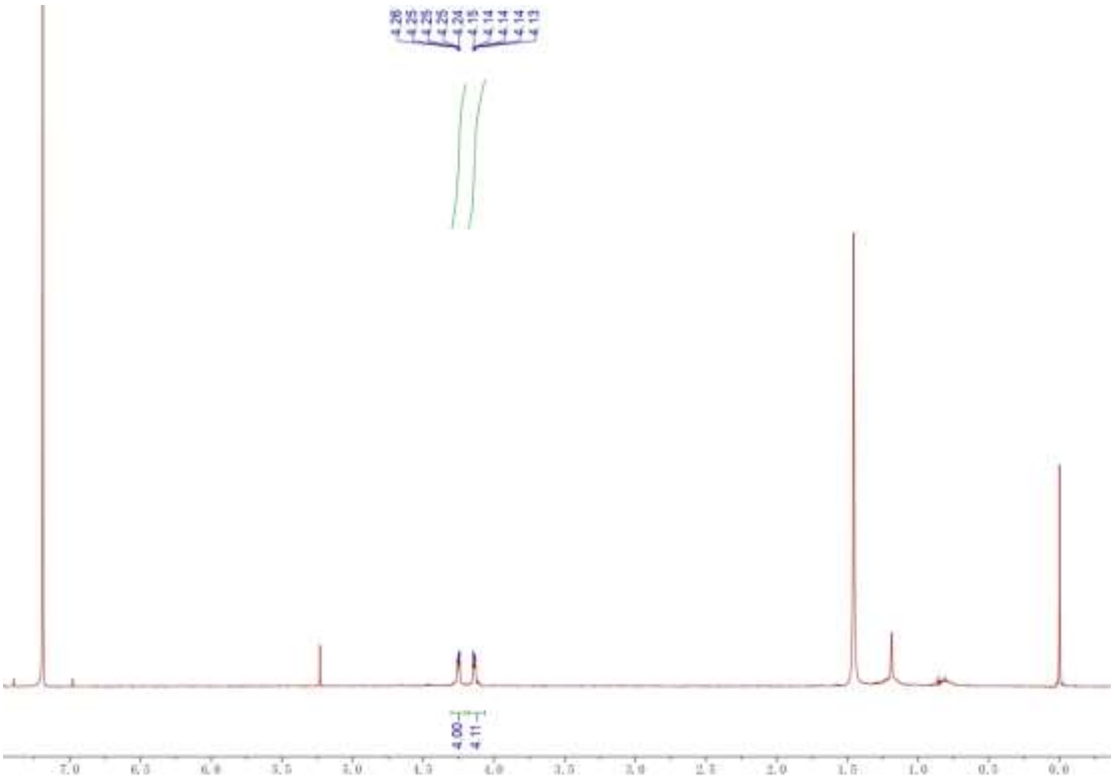

Figure S23. ^{1}H NMR spectrum of 11.

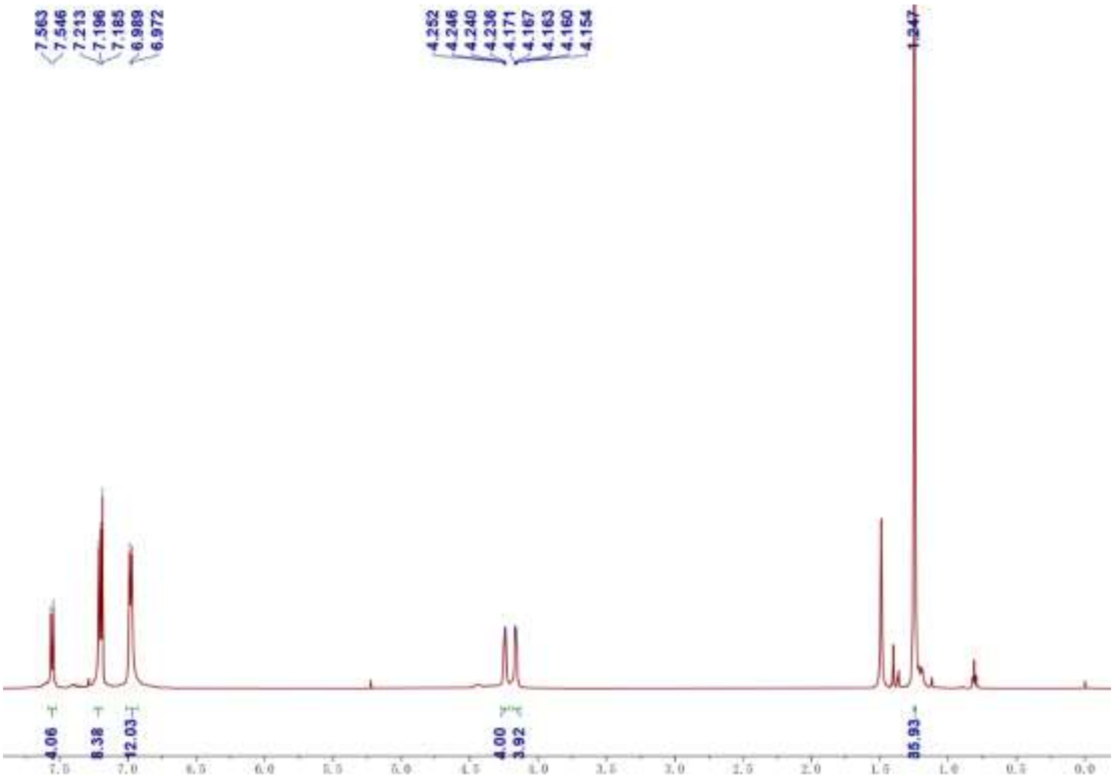

Figure S24. ^{1}H NMR spectrum of 12.

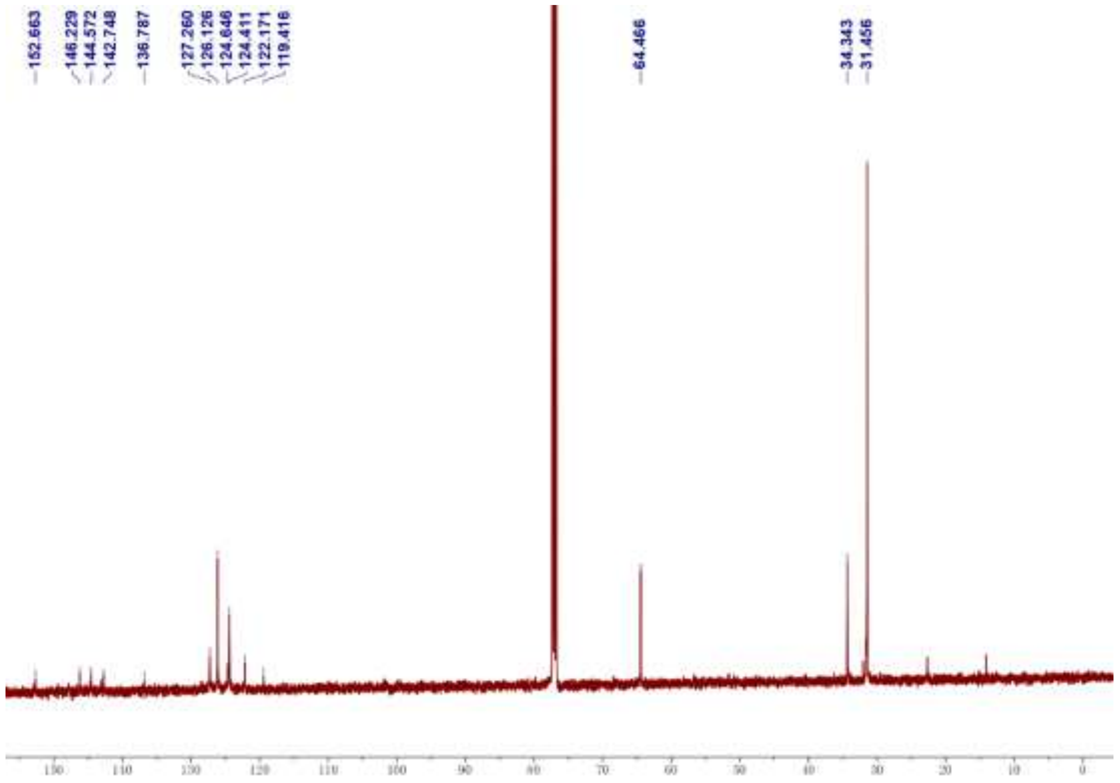

Figure S25. ^{13}C NMR spectrum of 12.

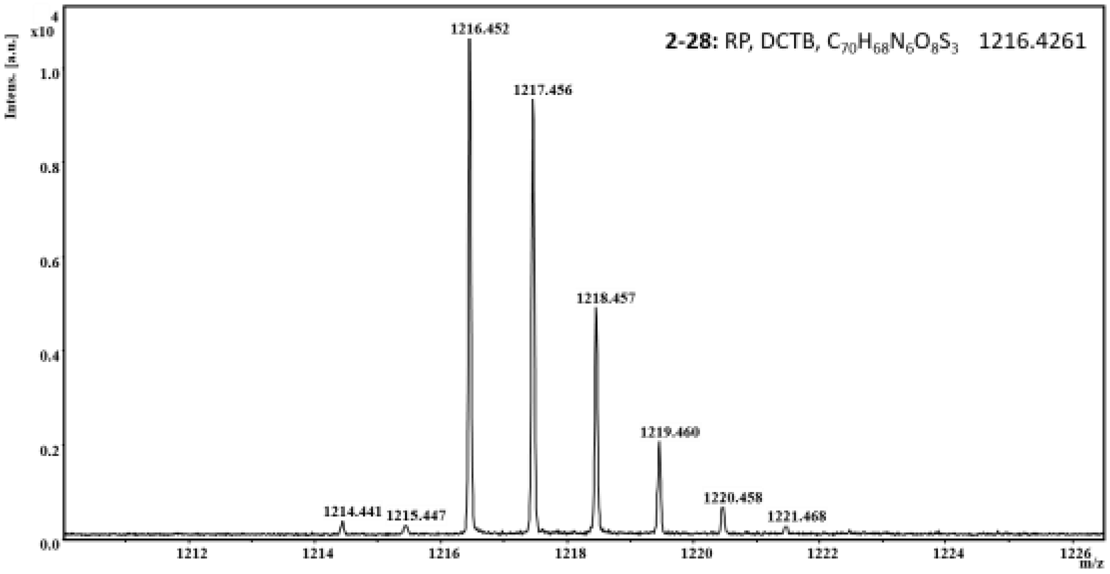

Figure S26. HRMS spectrum of 12.

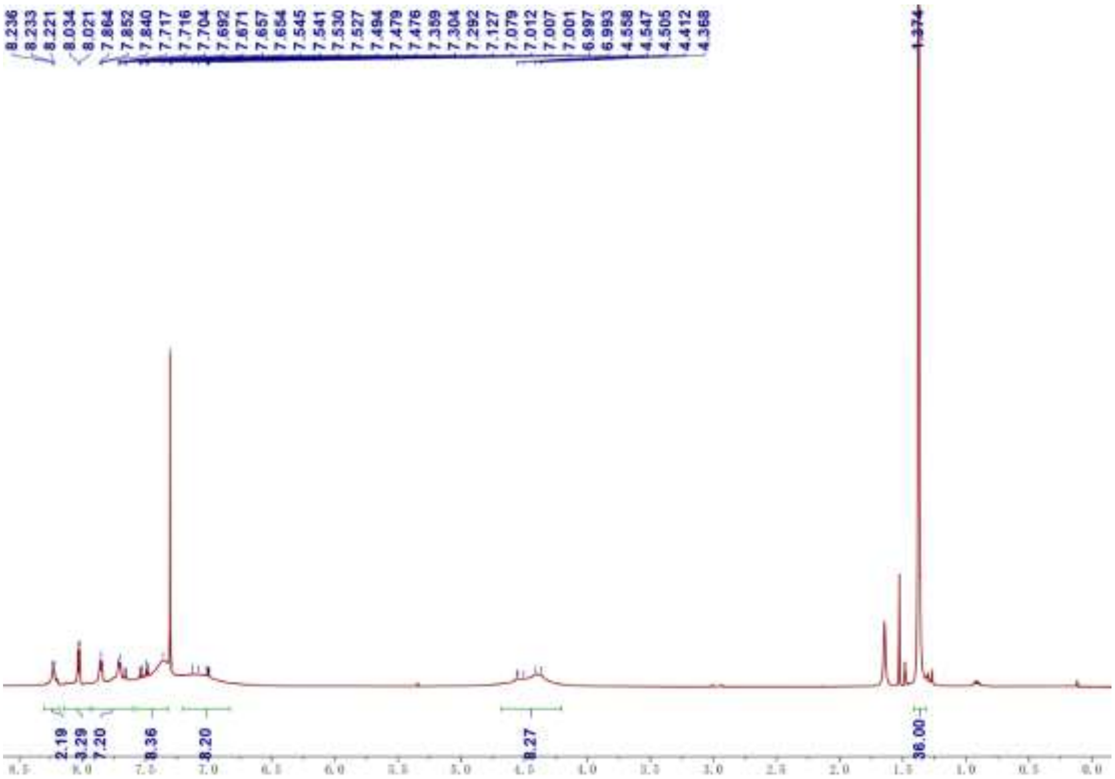

Figure S27. ^{1}H NMR spectrum of OTTITQ.

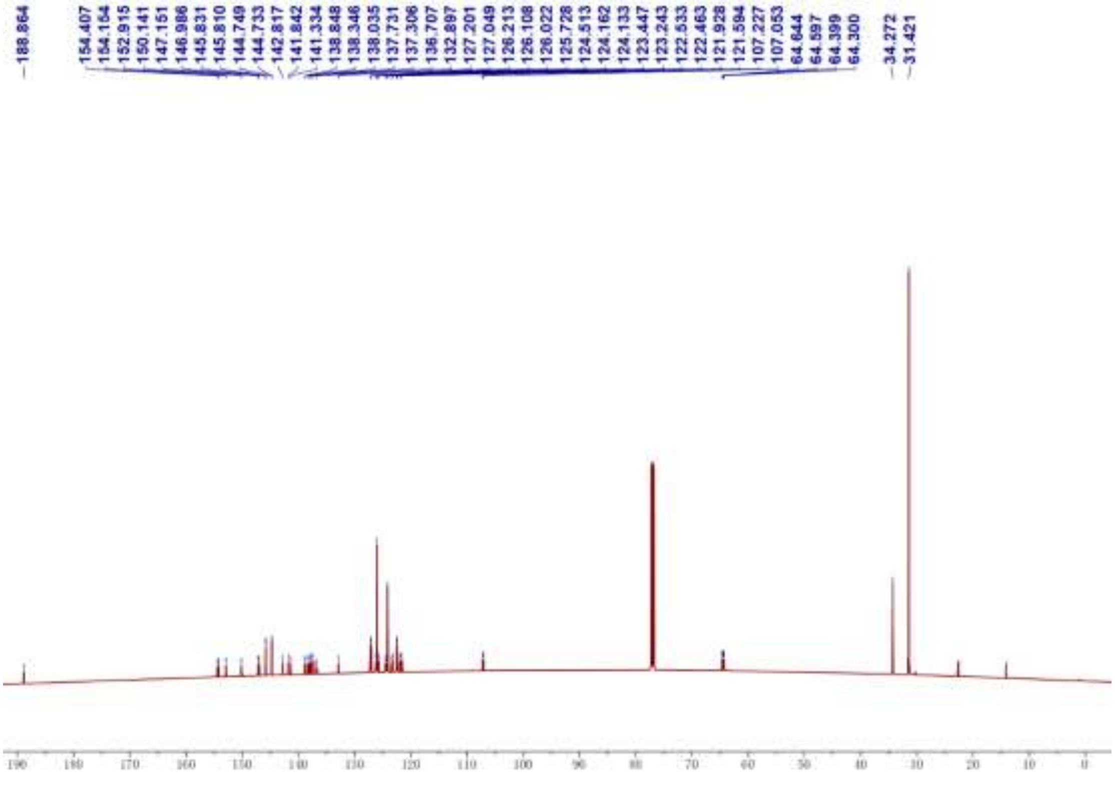

Figure S28. ^{13}C NMR spectrum of OTTITQ.

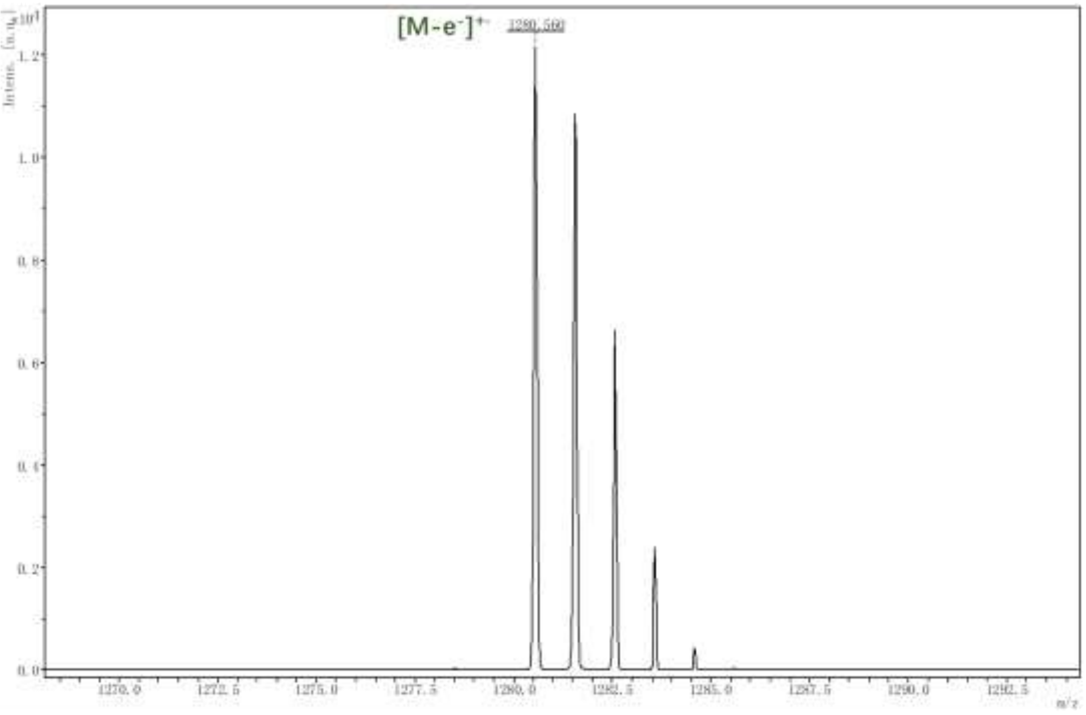

Figure S29. HRMS spectrum of OTTITQ.

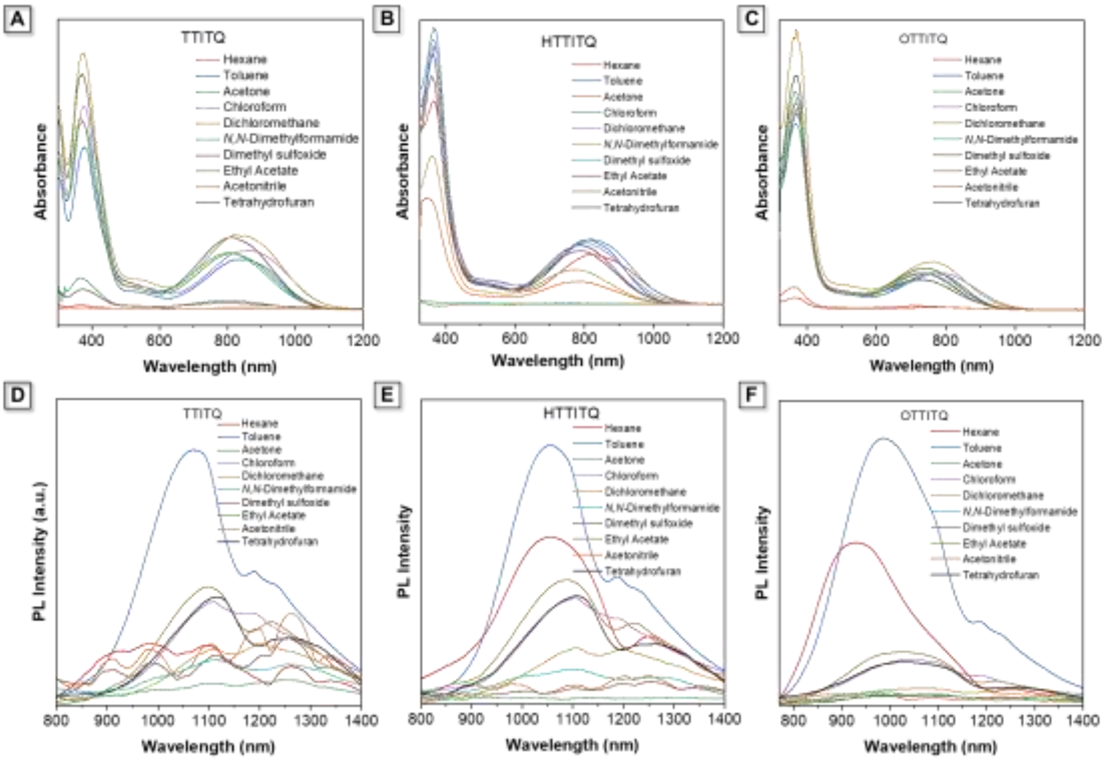

Figure S30. Absorption and emission spectra of (A, D) TTITQ, (B, E) HTTITQ and (C, F) OTTITQ in different solvents; Concentration: 50 μM in absorption spectra and 10 μM in emission spectra.

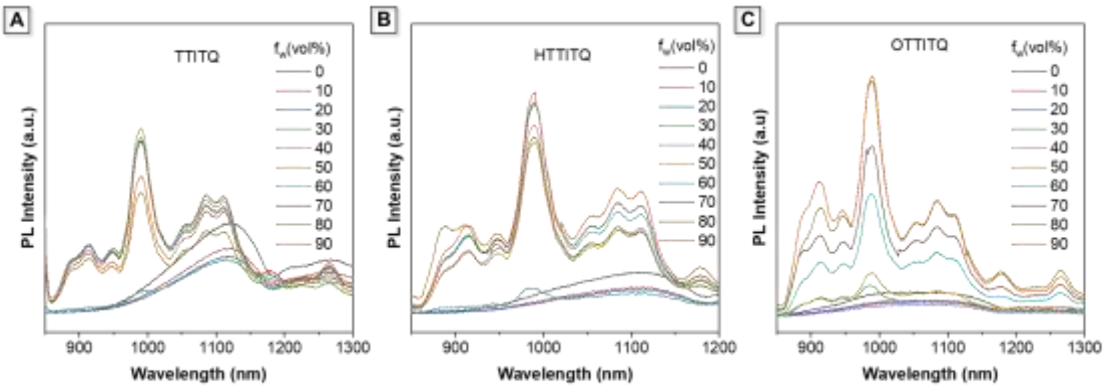

Figure S31. PL spectra of (A) TTITQ (λ_{ex}: 816 nm), (B) HTTITQ (λ_{ex}: 793 nm), (C) OTTITQ (λ_{ex}: 746 nm) in THF/H_2O with different H_2O fractions (f_W); Concentration: 5 µM.

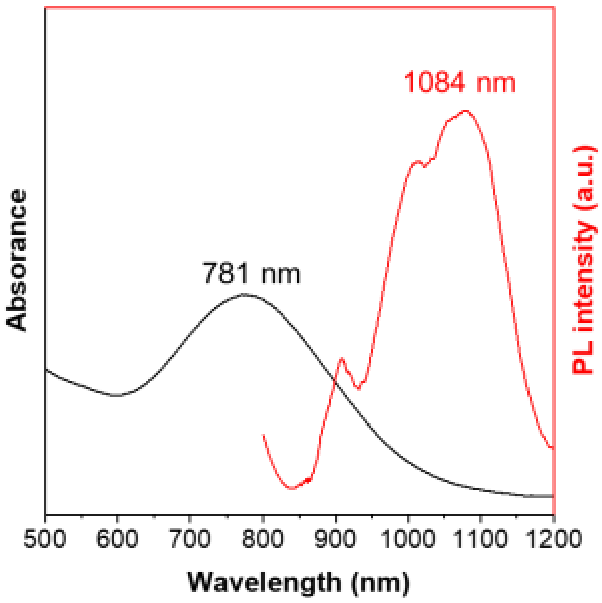

Figure S32. The absorption (black line) and emission (red line) spectra of OTTITQ NPs in aqueous solution.

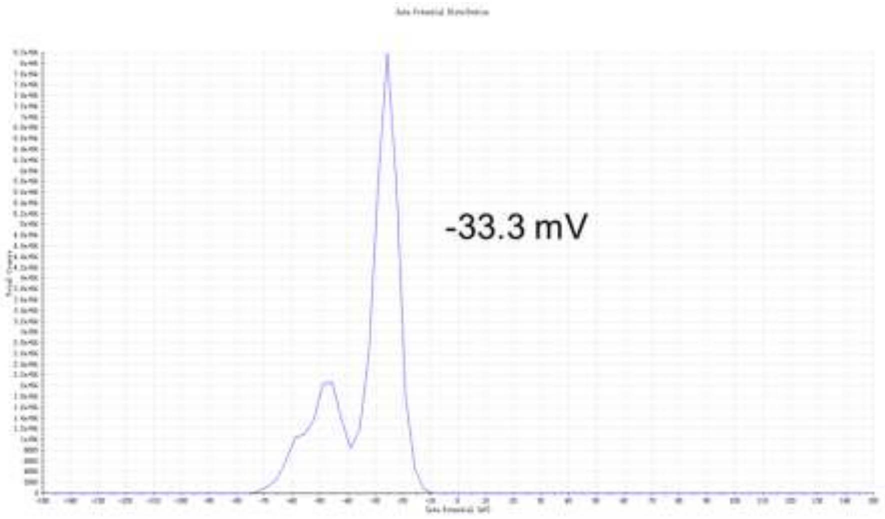

Figure S33. The zeta potential of freshly papered OTTITQ NPs.

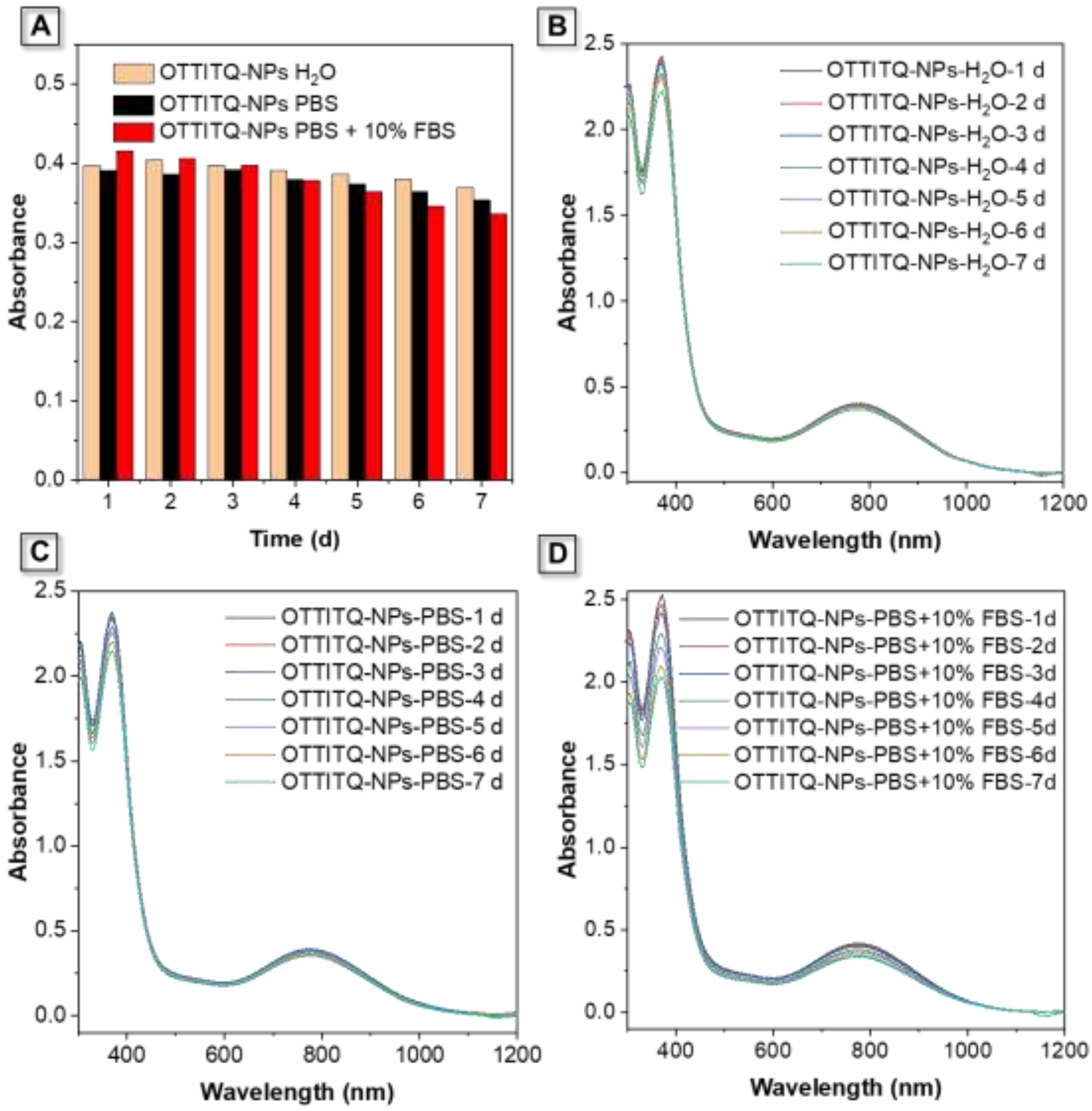

Figure S34. Stability analysis for (A) size variation and (B) absorption changes of OTTITQ NPs at room temperature in H_2O, PBS or PBS + 10% FBS measured by DLS. Absorption spectrum of OTTITQ NPs in (C) H_2O, (D) PBS, (E) PBS+10% FBS for seven days.

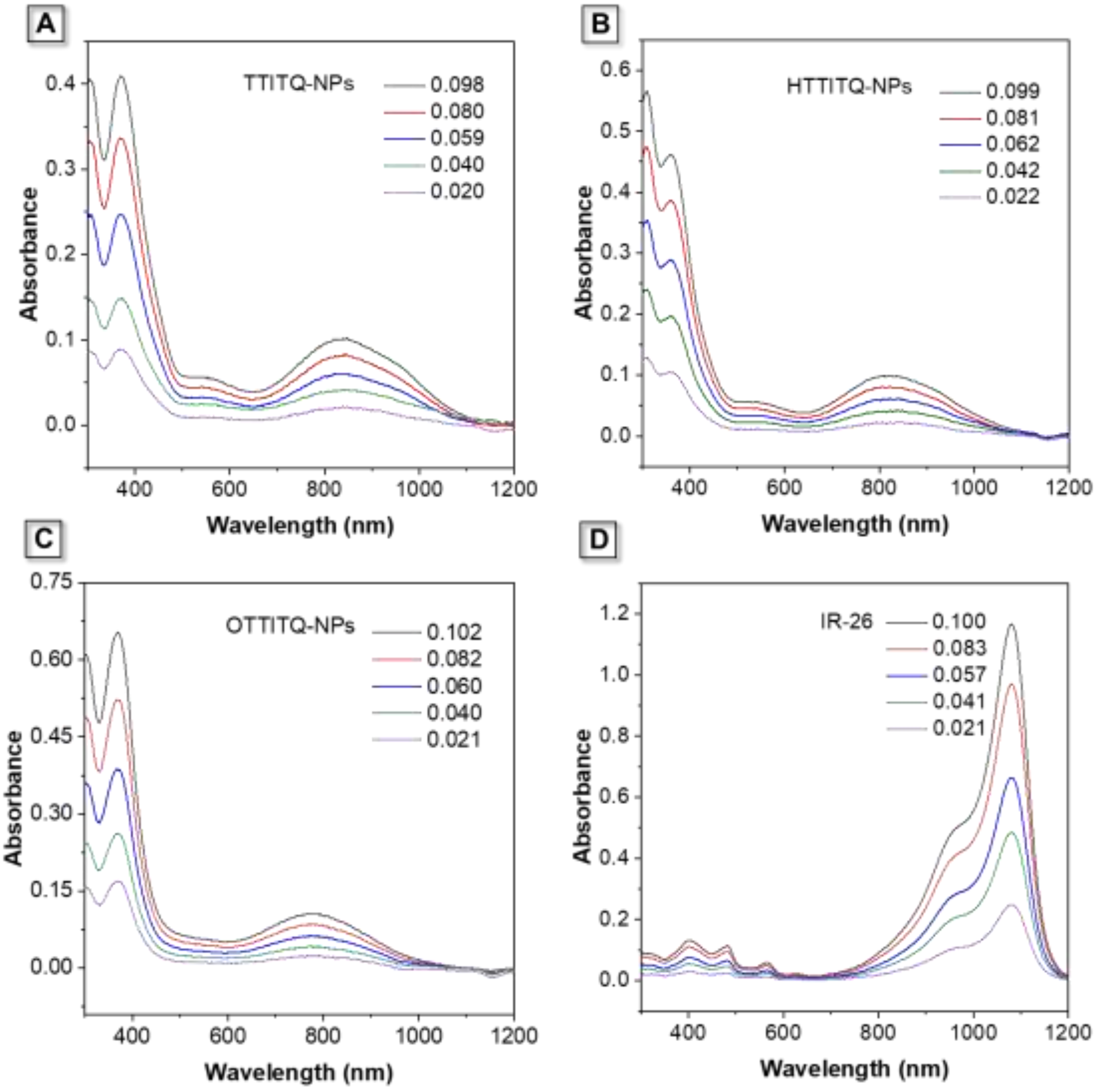

Figure S35. UV-Vis-NIR absorption spectra of (A) TTITQ NPs, (B) HTTITQ NPs, (C) OTTITQ NPs, (D) IR-26.

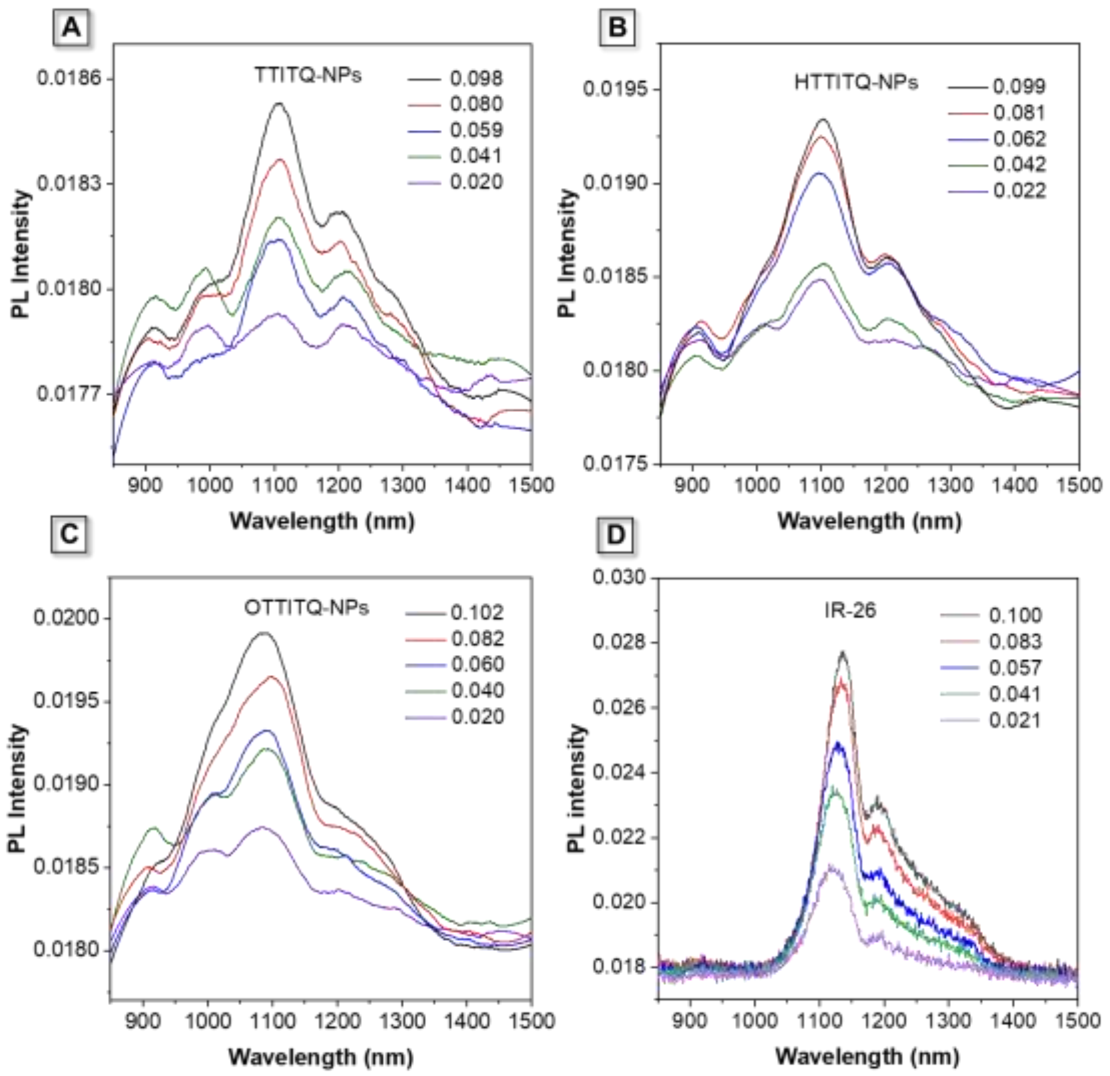

Figure S36. At a wavelength of 808 nm, the ultraviolet absorbance ranges from 0.2 to 1.0, pertaining to the fluorescence spectrum of (A) TTITQ NPs, (B) HTTITQ NPs, (C) OTTITQ NPs, (D) IR-26.

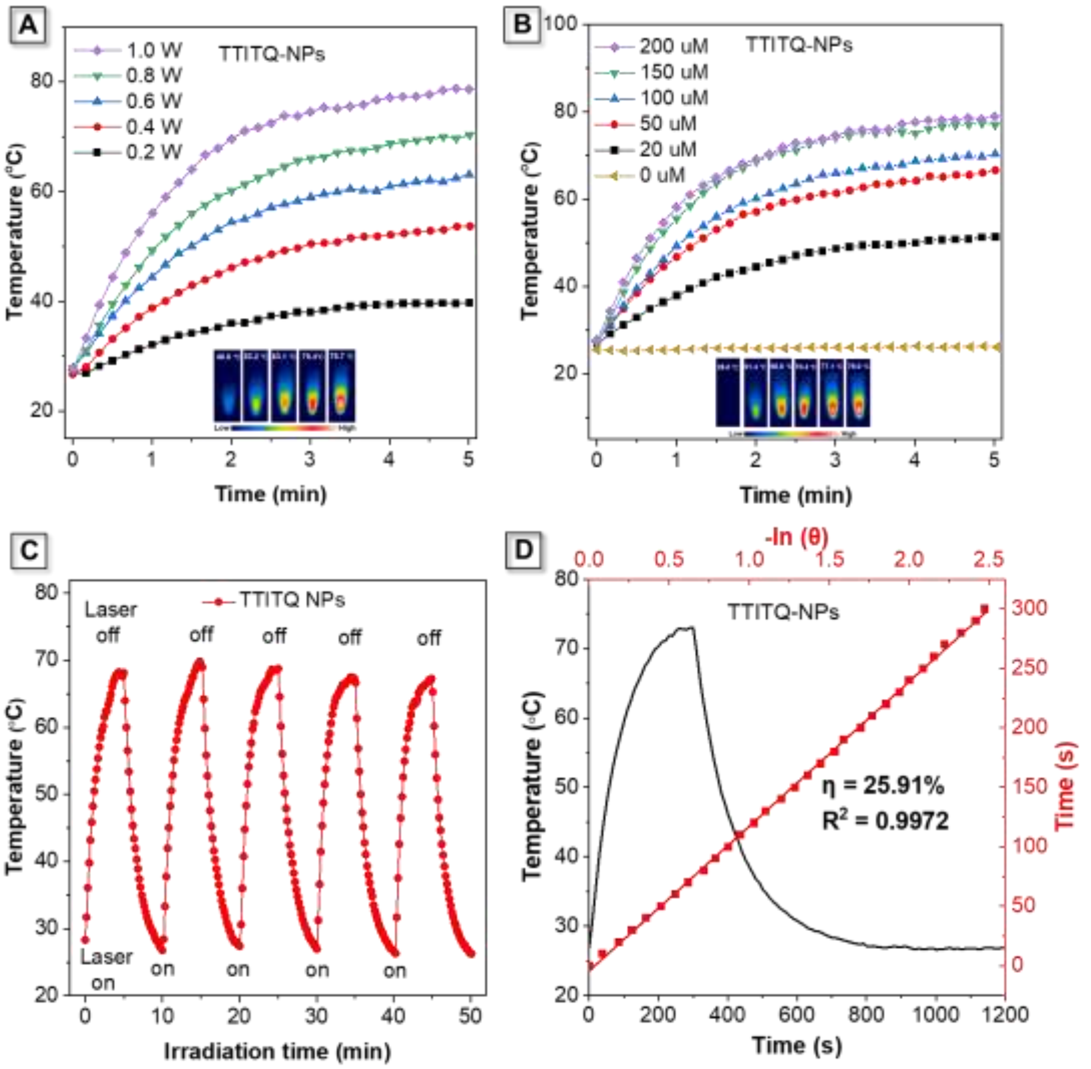

Figure S37. Photothermal heating behaviors of TTITQ NPs (A) at different power (100 μM), (B) at different concentrations upon 808 nm (0.8 W cm^{-2}) laser irradiation, (C) Photothermal stability of TTITQ NPs upon 808 nm (0.8 W cm^{-2}) laser irradiation, (D) Photothermal performance of TTITQ NPs (100 μM) by cooling to room temperature with linear analysis.

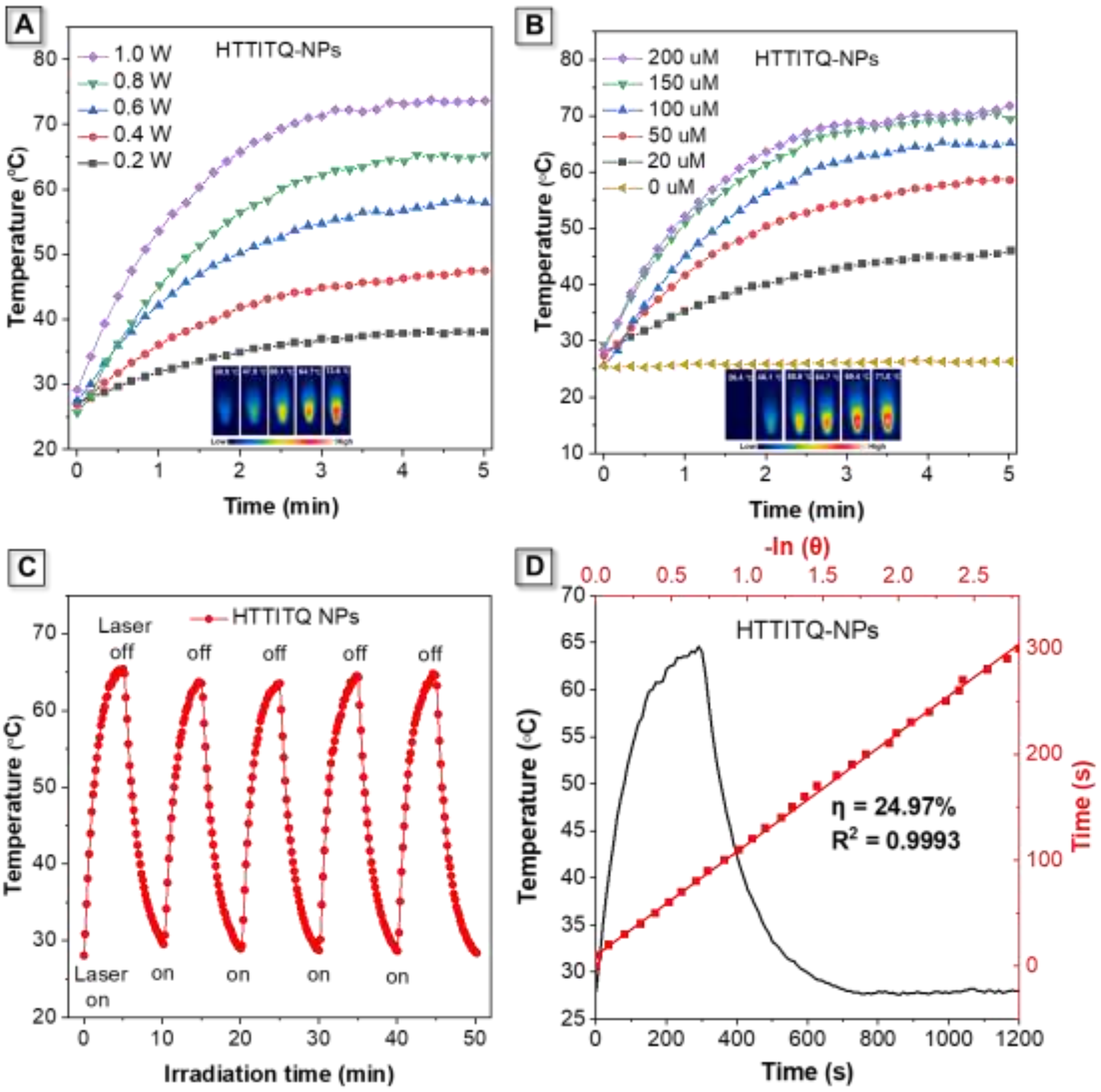

Figure S38. Photothermal heating behaviors of HTTITQ NPs (A) at different power (100 µM), (B) at different concentrations upon 808 nm (0.8 W cm^{-2}) laser irradiation, (C) Photothermal stability of HTTITQ NPs upon 808 nm (0.8 W cm^{-2}) laser irradiation, (D) Photothermal performance of HTTITQ NPs (100 µM) by cooling to room temperature with linear analysis.

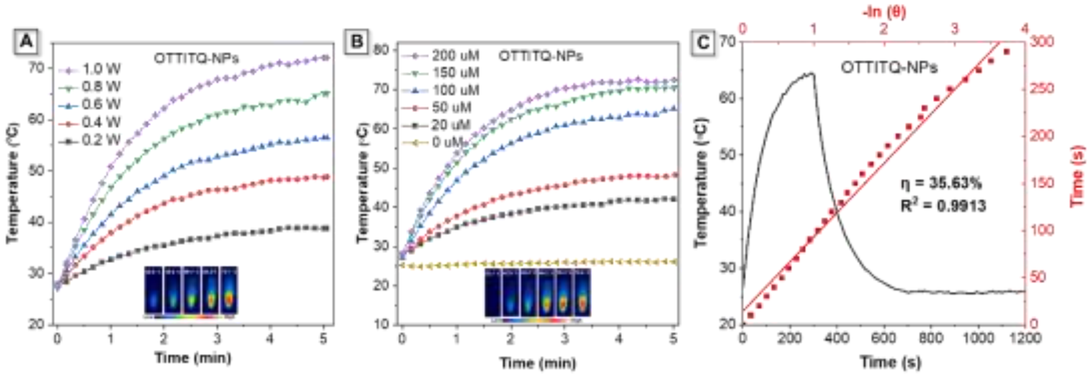

Figure S39. Photothermal heating behaviors of OTTITQ NPs (A) at different power (100 μM), (B) at different concentrations upon 808 nm (0.8 W cm^{-2}) laser irradiation, (C) Photothermal performance of OTTITQ NPs (100 μM) by cooling to room temperature with linear analysis.

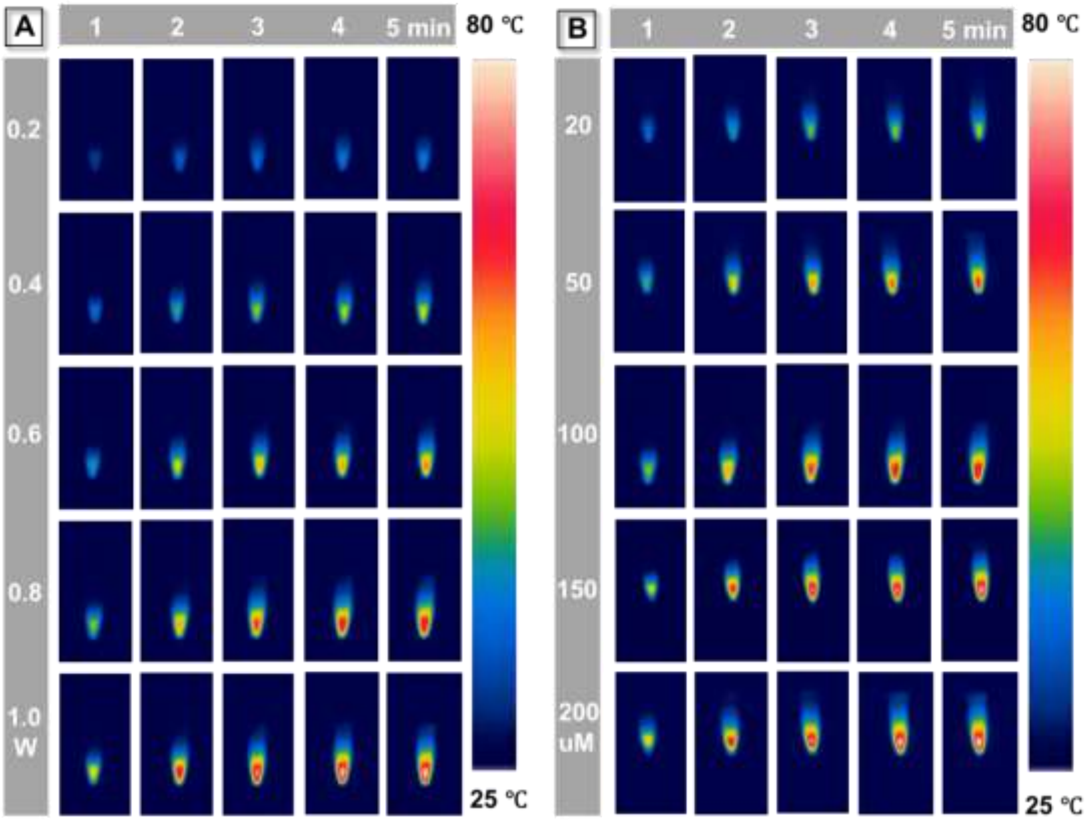

Figure S40. Thermal images of TTITQ NPs (A) at different power (100 μM), (B) at different concentrations upon 808 nm laser irradiation.

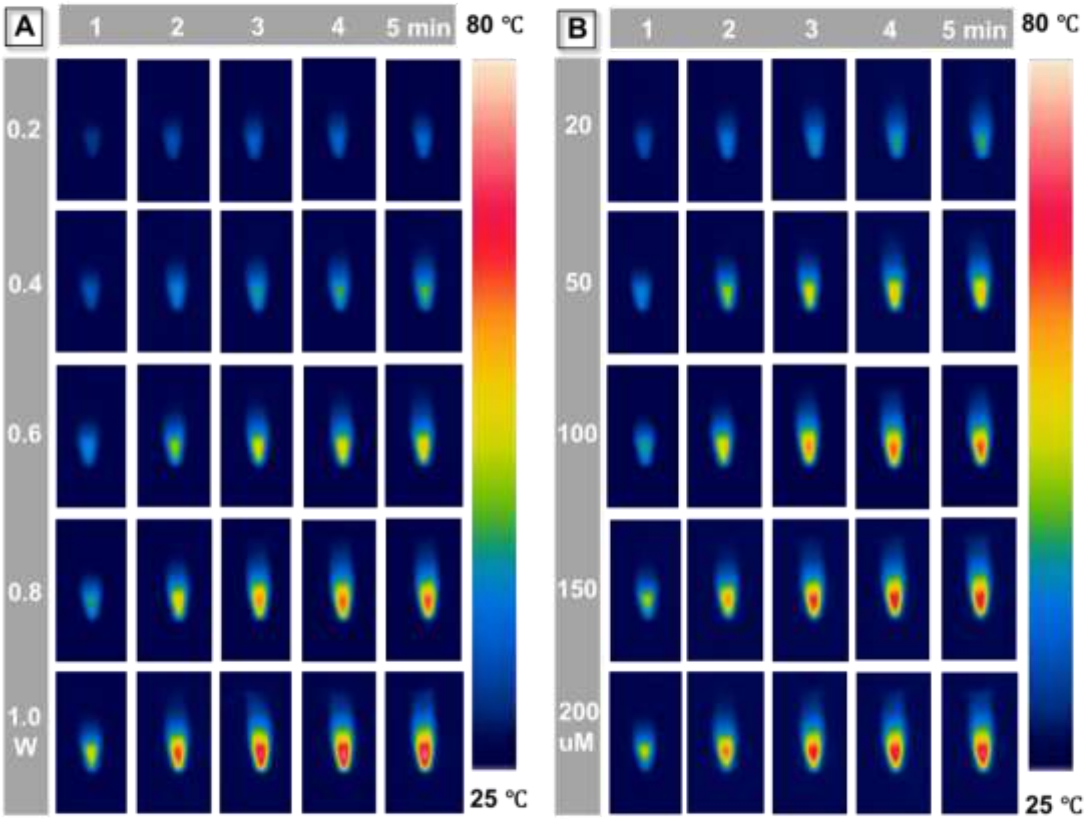

Figure S41. Thermal images of HTTITQ NPs (A) at different power (100 μM), (B) at different concentrations upon 808 nm laser irradiation.

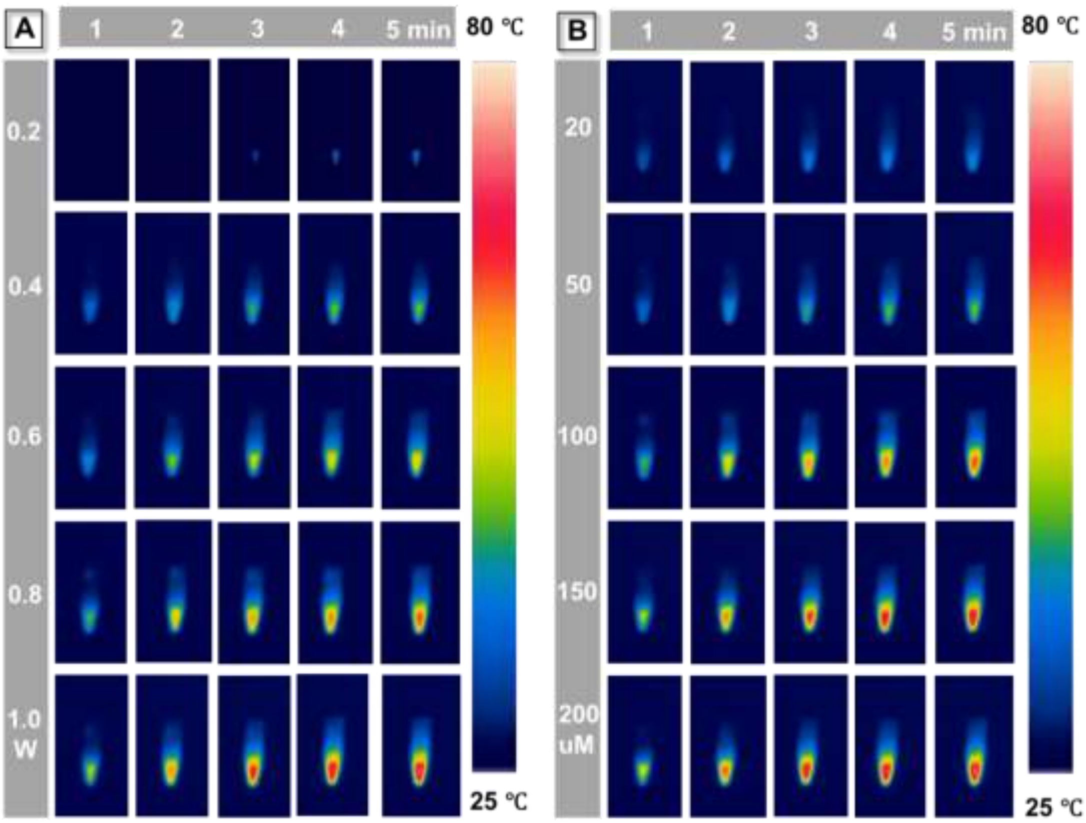

Figure S42. Thermal images of OTTITQ NPs (A) at diff erent power (100 μM), (B) at diff erent concentrations upon 808 nm laser irradiation.

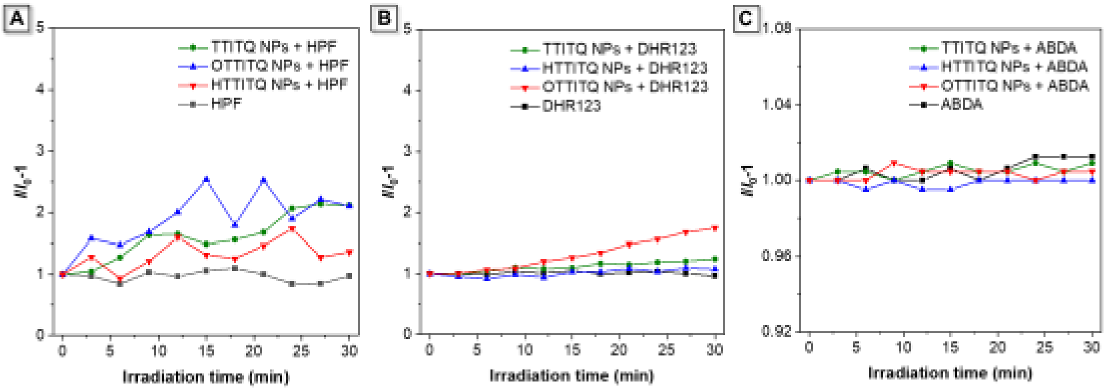

Figure S43. ROS generation of AIEgens NPs (2 μM) upon 808 nm (0.8 W cm^{-2}) laser irradiation for different times by using (A) HPF, (B) DHR123, (C) ABDA as an indicator for overall ROS detection.

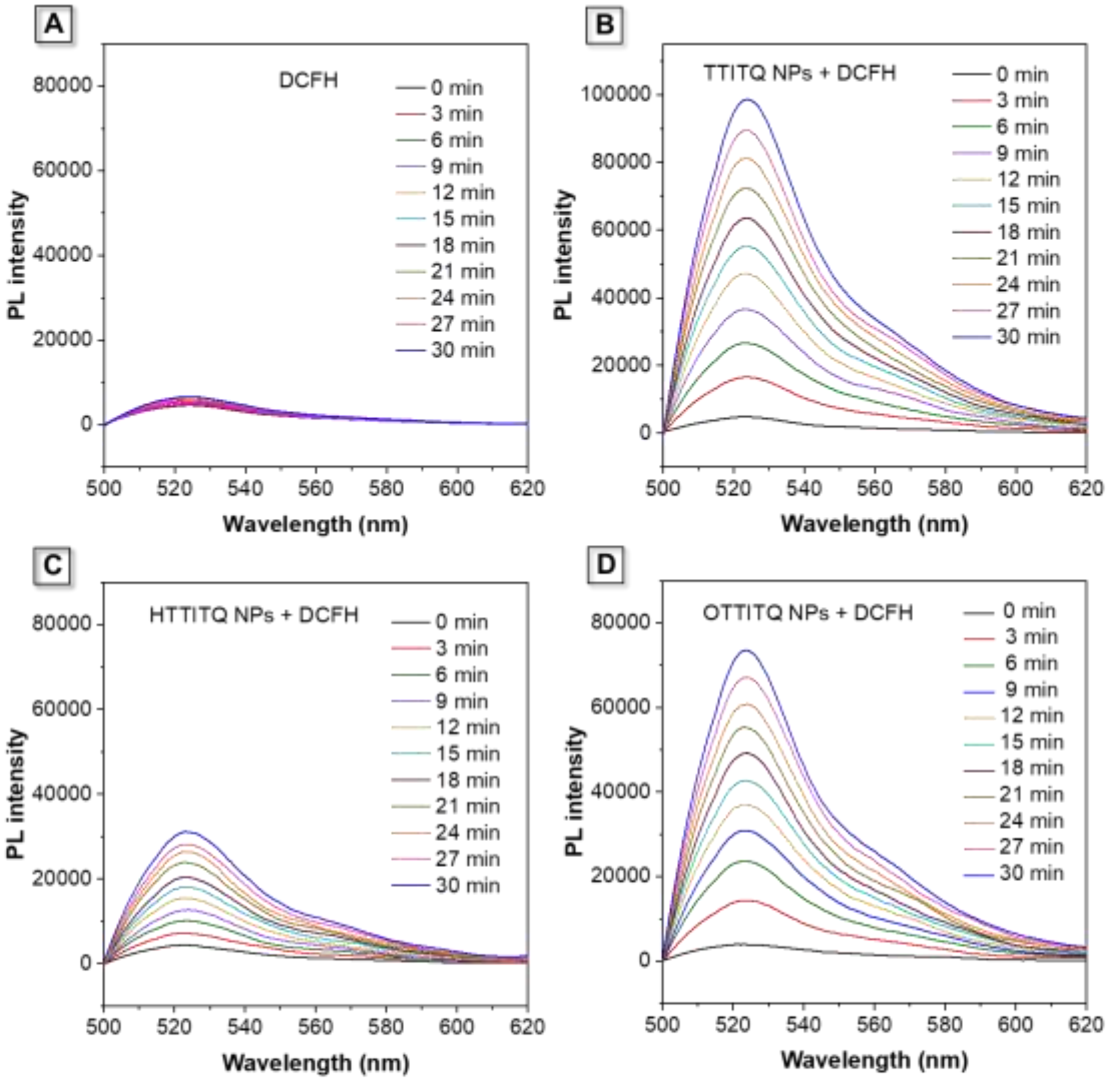

Figure S44. ROS generation of AIEgens NPs (2 μM) upon 808 nm (0.8 W cm^{-2}) laser irradiation for different times by using DCFH as an indicator for overall ROS detection. Relative changes in PL intensity of (A) Blank (DCFH alone), (B) TTITQ NPs + DCFH, (C) HTTITQ NPs + DCFH, and (D) OTTITQ NPs + DCFH.

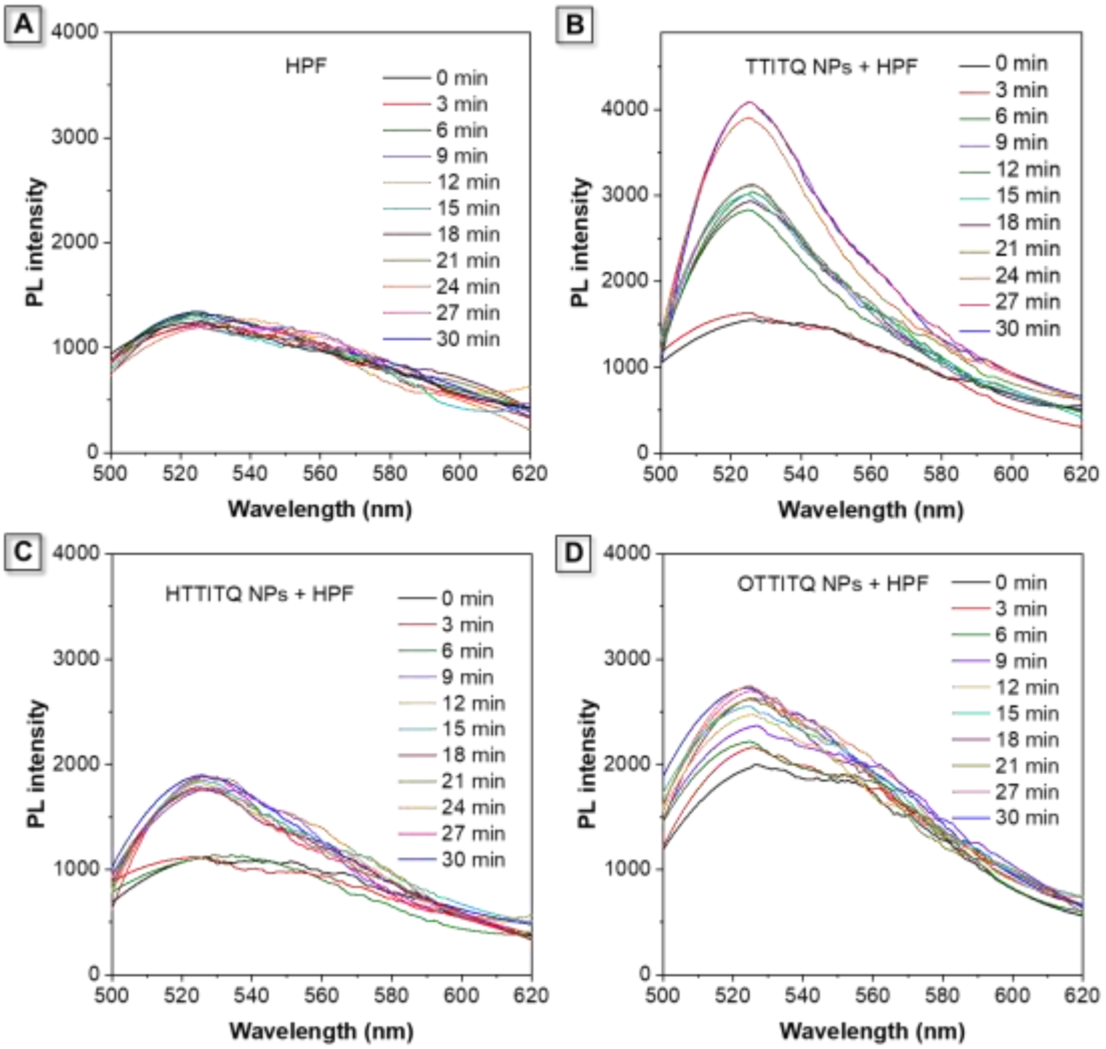

Figure S45. ROS generation of AIEgens NPs (2 μM) upon 808 nm (0.8 W cm^{-2}) laser irradiation for different times by using HPF as an indicator for overall ROS detection. Relative changes in PL intensity of (A) Blank (HPF alone), (B) TTITQ NPs + HPF, (C) HTTITQ NPs + HPF, (D) OTTITQ NPs + HPF.

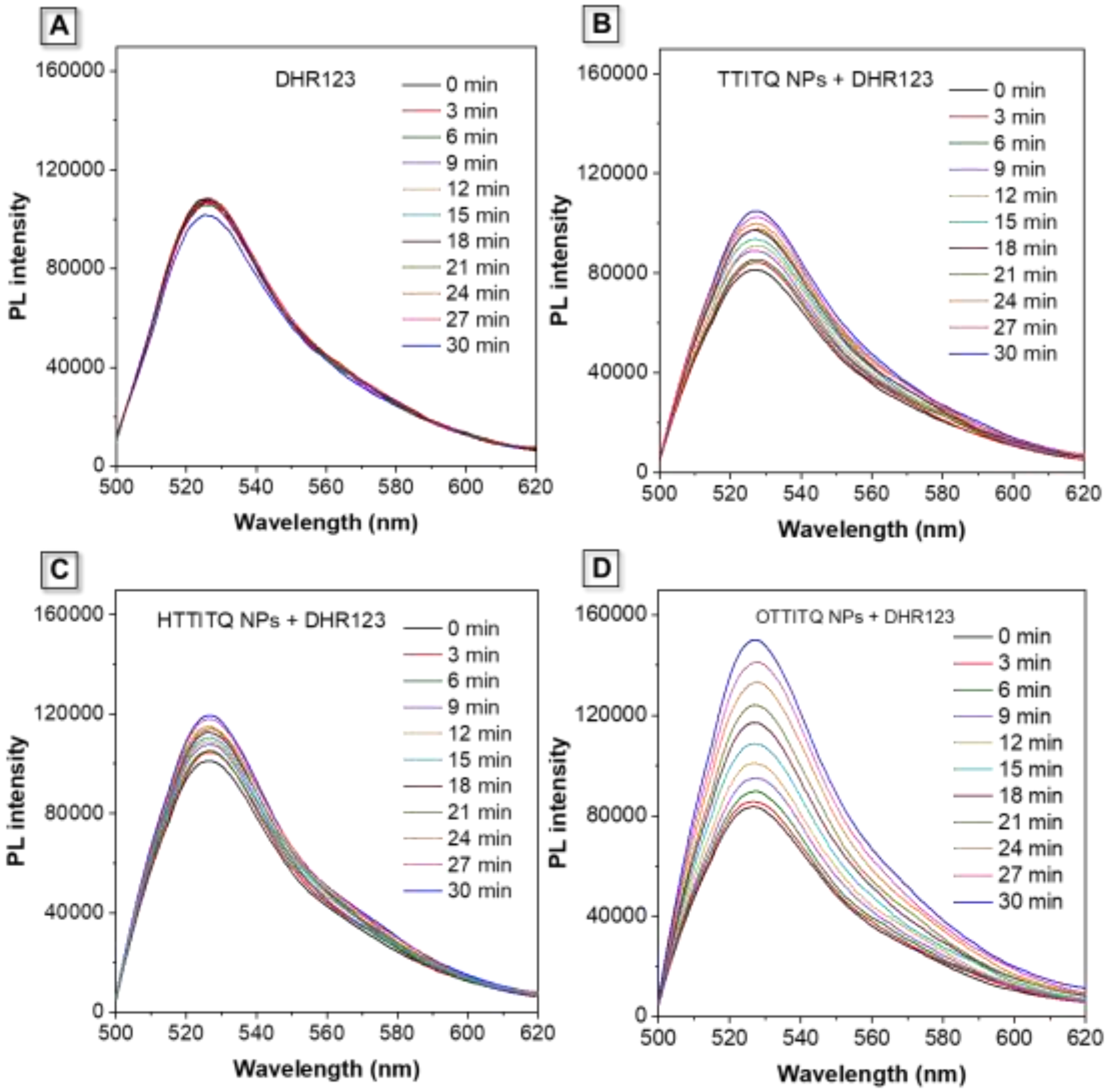

Figure S46. ROS generation of AIEgens NPs (2 μM) upon 808 nm (0.8 W cm^{-2}) laser irradiation for different times by using DHR123 as an indicator for overall ROS detection. Relative changes in PL intensity of (A) Blank (DHR123 alone), (B) TTITQ NPs + DHR123, (C) HTTITQ NPs + DHR123, (D) OTTITQ NPs + DHR123.

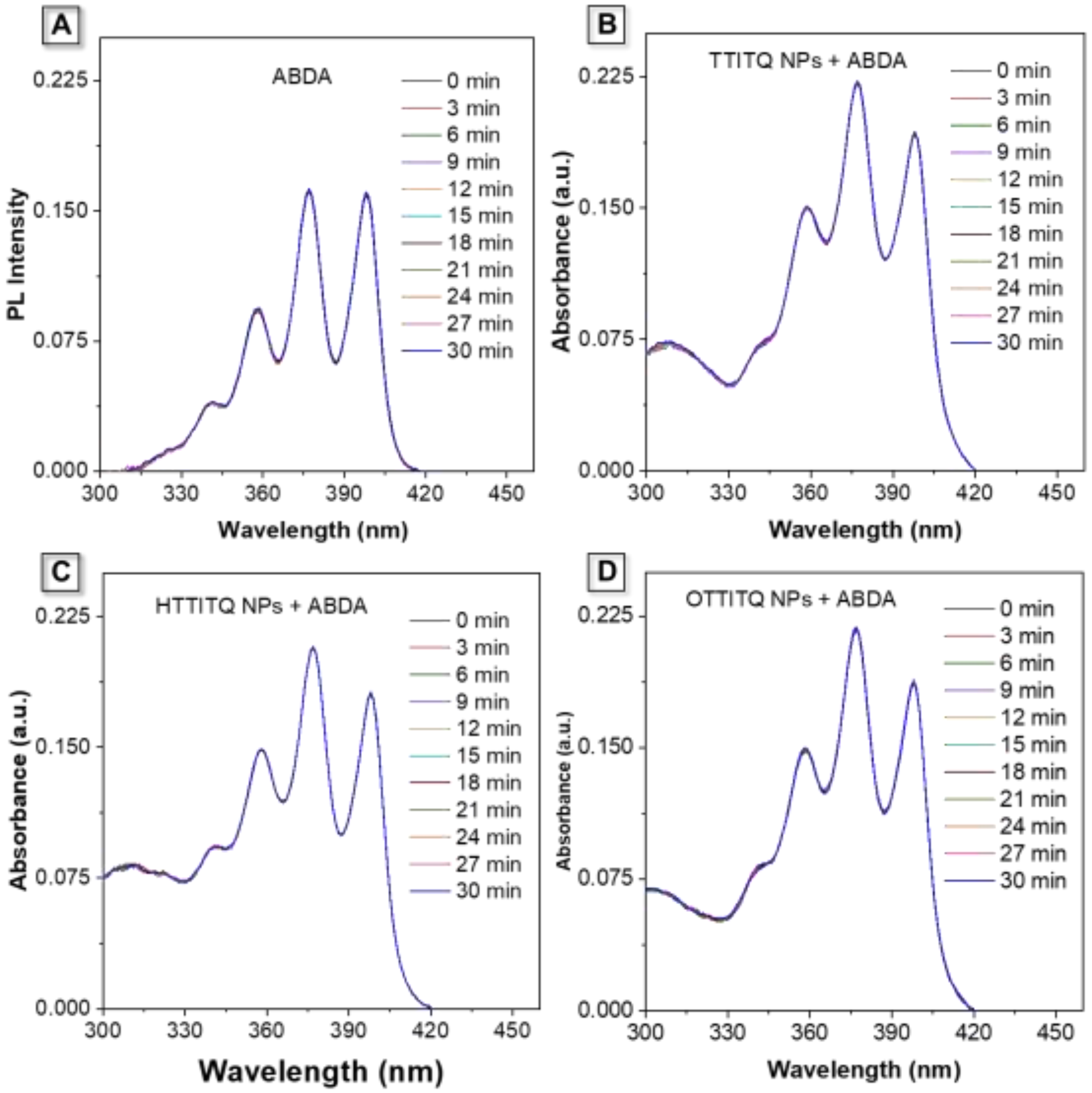

Figure S47. ROS generation of AIEgens NPs (2 μM) upon 808 nm (0.8 W cm^{-2}) laser irradiation for different times by using ABDA as an indicator for overall ROS detection. Relative changes in PL intensity of (A) Blank (ABDA alone), (B) TTITQ NPs + ABDA, (C) HTTITQ NPs + ABDA, (D) OTTITQ NPs + ABDA.

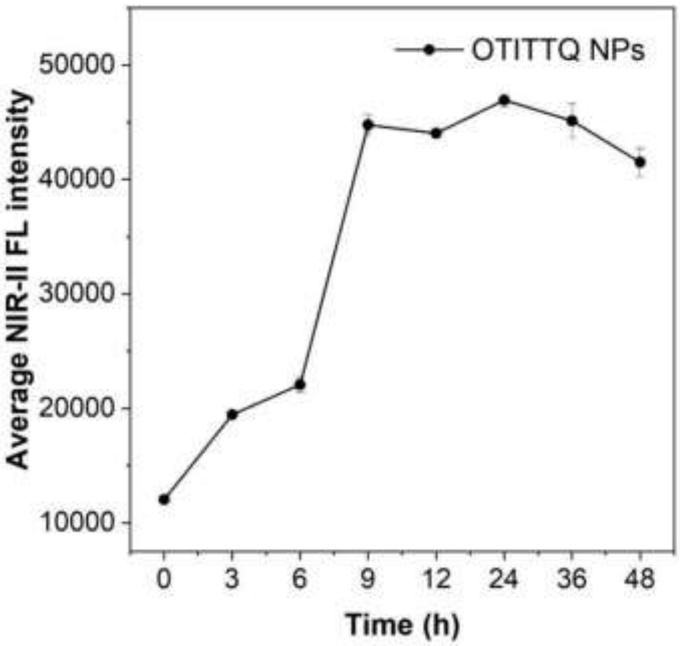

Figure S48. NIR-II fluorescence intensity of tumor as a function of time before (0 h, pre-injection) and after intravenous injection of OTTITQ NPs.

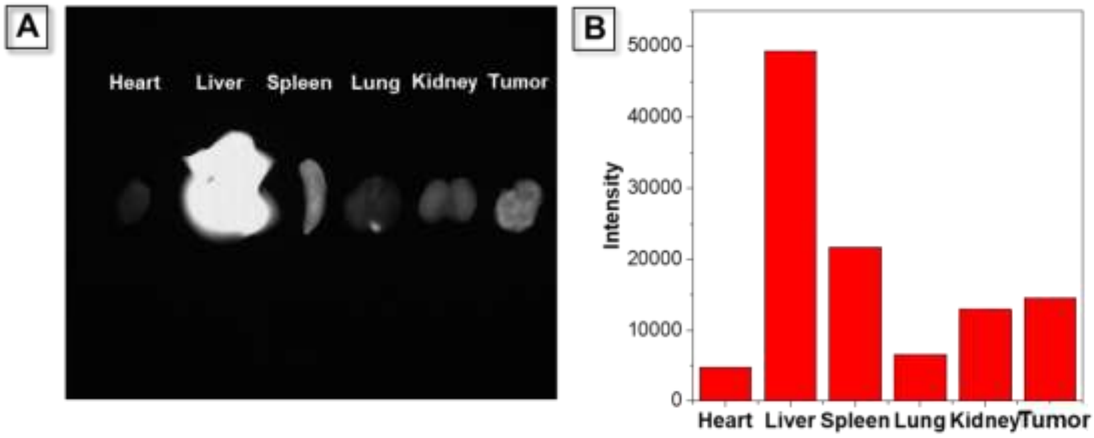

Figure S49. (A) Ex vivo NIR-II fluorescence images of major organs and tumor. (B) The corresponding mean fluorescence intensity after intravenous injection with OTTITQ NPs.

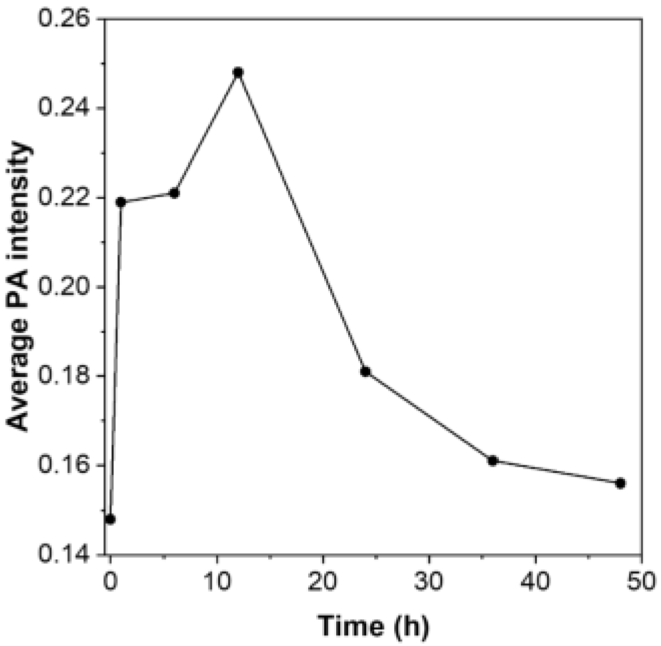

Figure S50. PA intensity of tumor as a function of time before (0 h, pre-injection) and after intravenous injection of OTTITQ NPs.

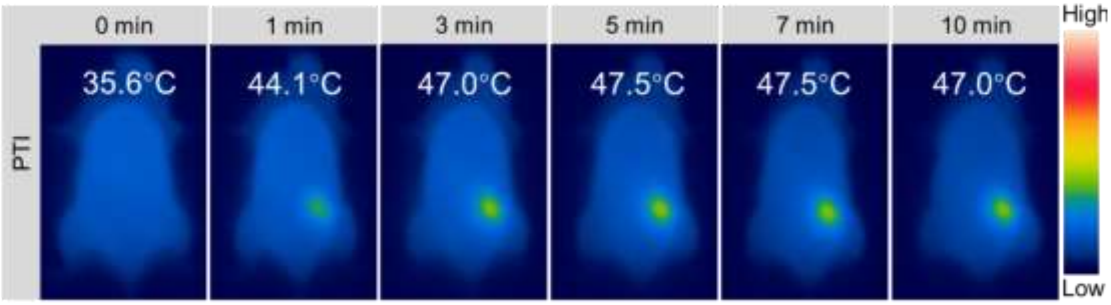

Figure S51. (A) PTI of MB49 bladder cancer mice at different time after intravenous injection of PBS, 24 h after the injection, 808 nm laser (0.8 W cm^{-2}) irradiation for 0, 1, 3, 5, 7 and 10 min, respectively.

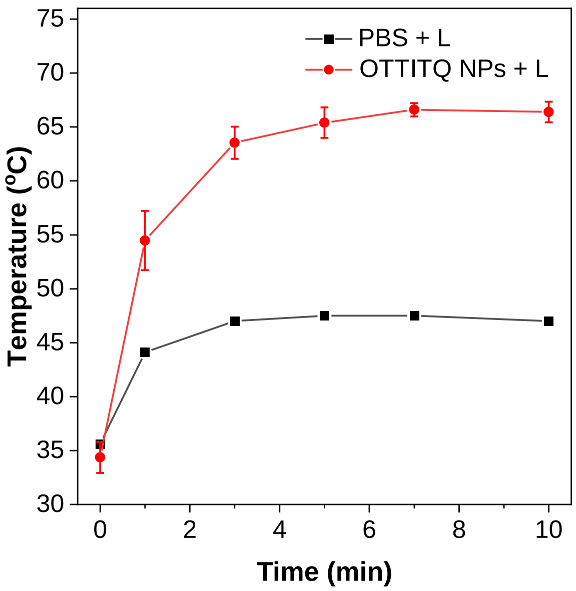

Figure S52. The corresponding statistic temperature elevation of the irradiated area (tumor sites)-time curves.

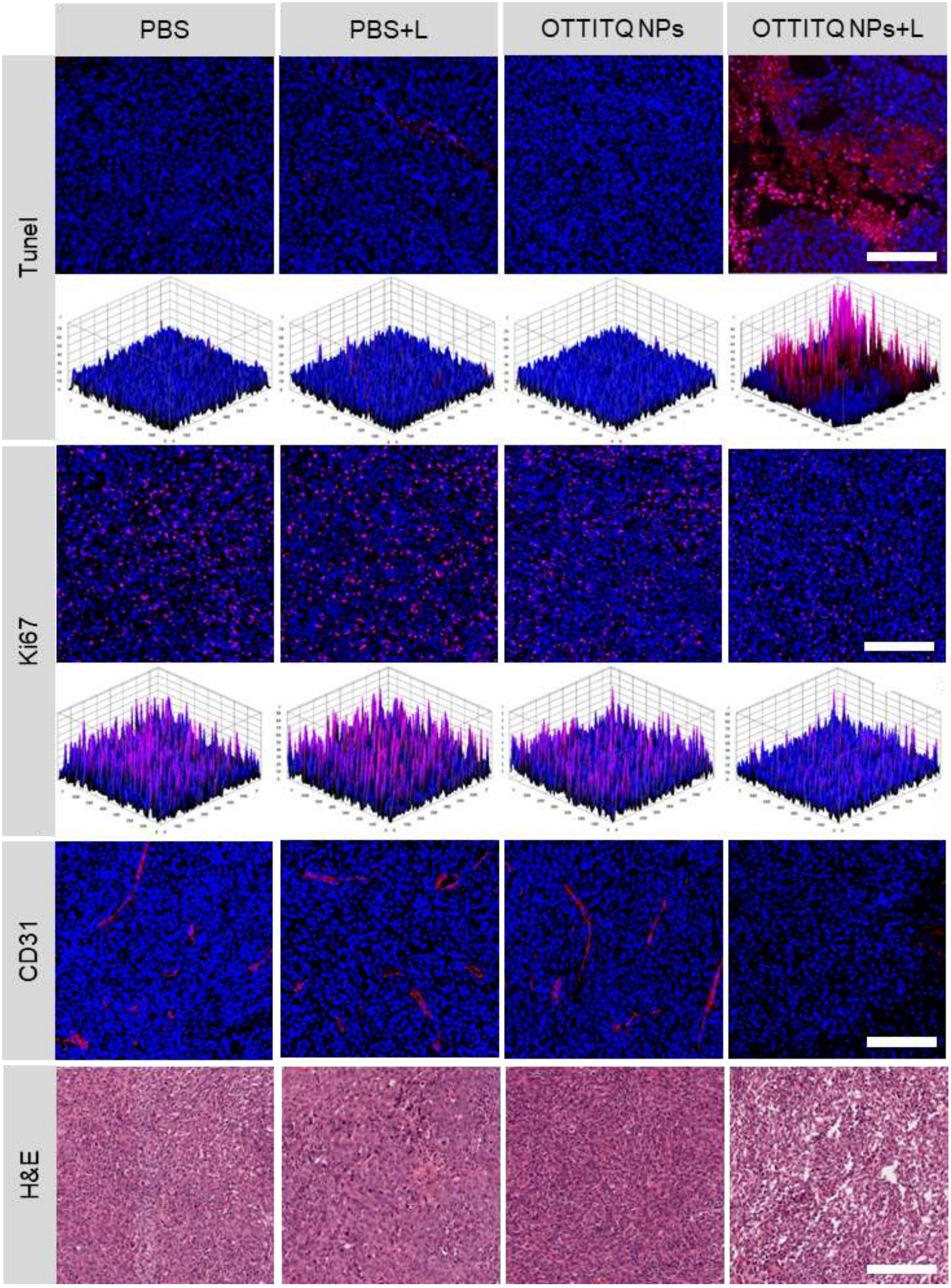

Figure S53. TUNEL, Ki67, CD31 and H&E, staining analyses of tumor tissues under various treatments. Scale bar: 100 μm.

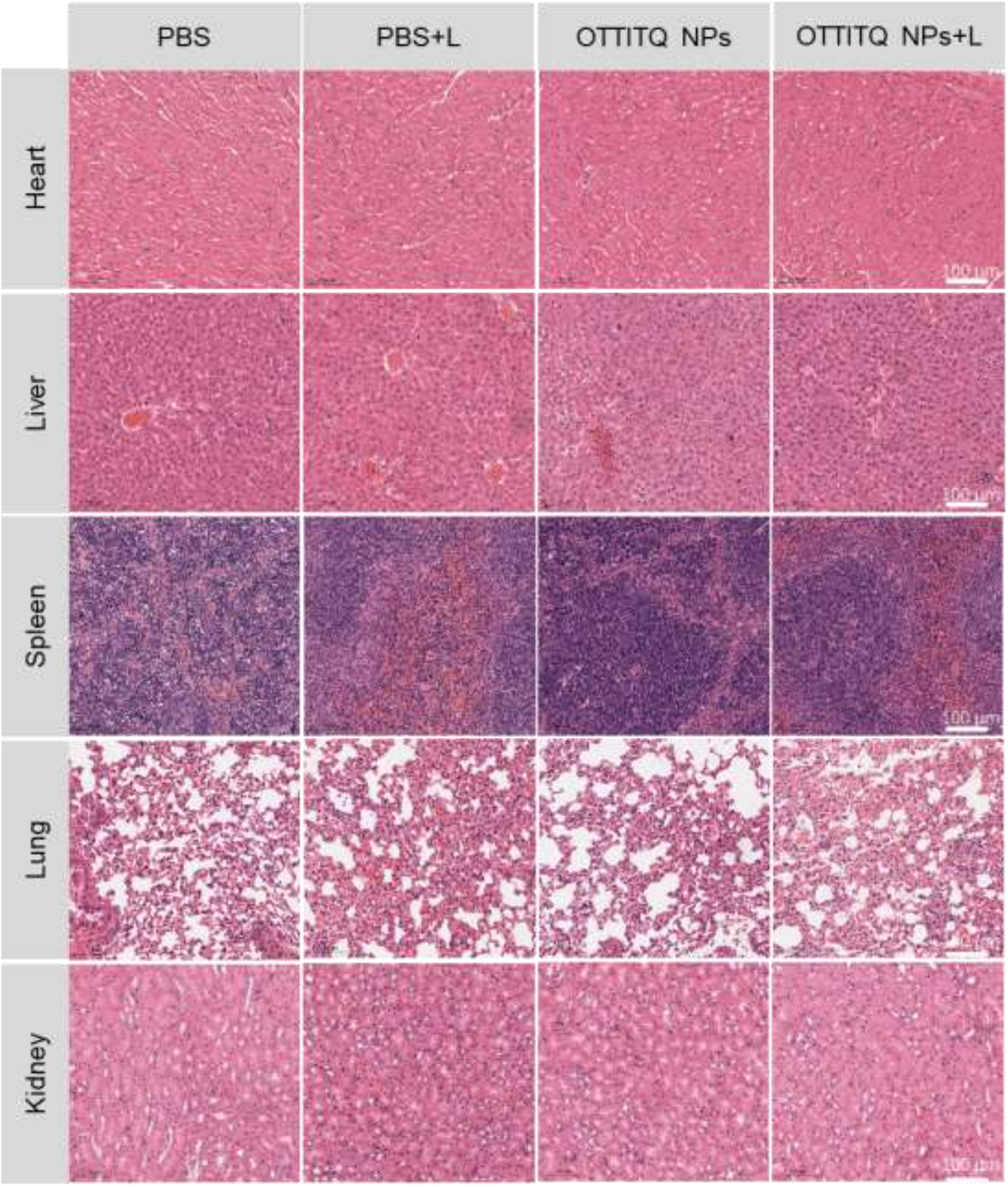

Figure S54. H&E staining analysis of major organs of MB49 bladder cancer mice after various treatment for 15 days. Scale bar: 100 μm.

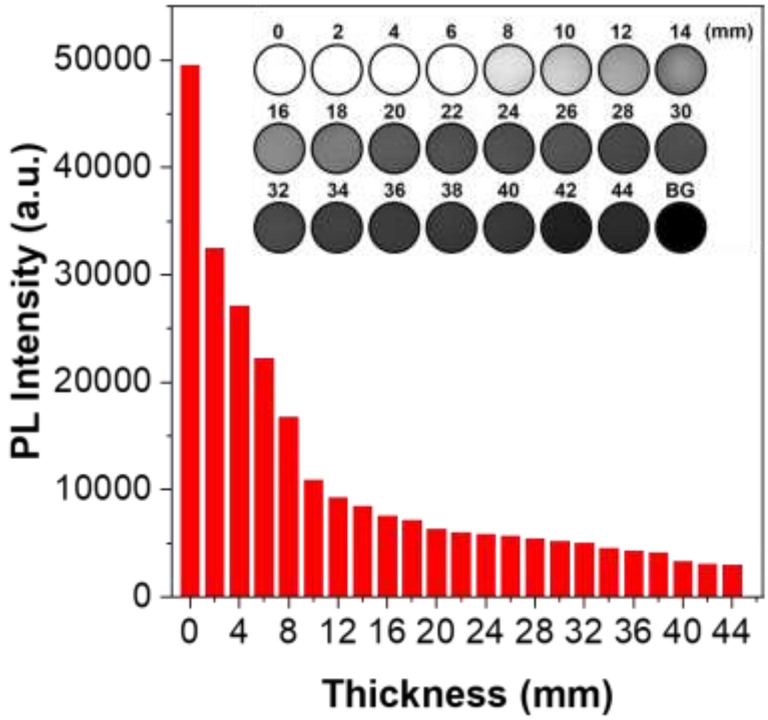

Figure S55. The NIR-II fluorescence intensity of OTTITQ NPs (1 mM) with different thicknesses of chicken tissues on top of the samples.

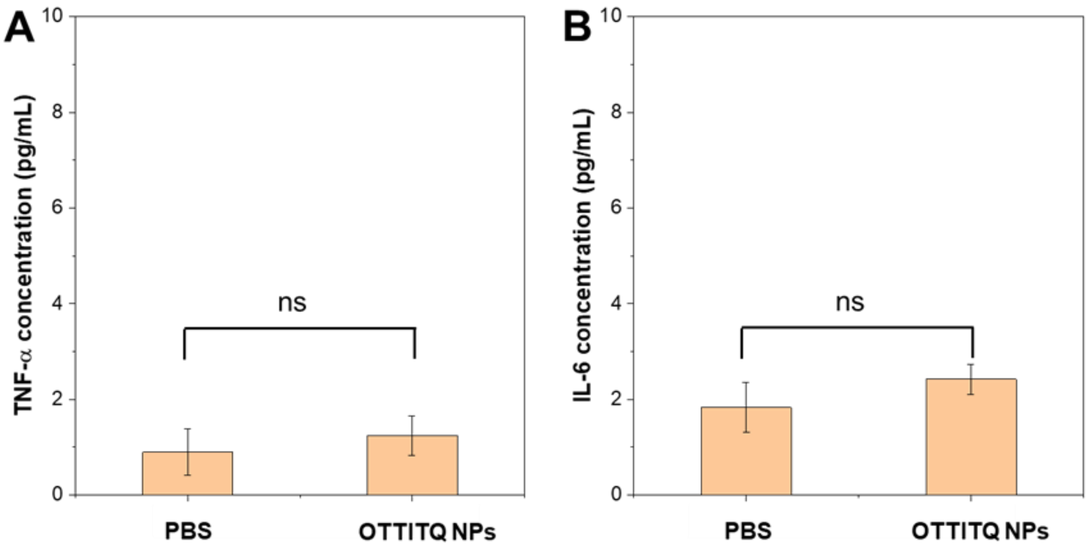

Figure S56. Inflammation-related biomarkers: TNF-α and IL-6.

Table S2. Cartesian coordinates for DFT optimized structure (at B3LYP/6-31g (d)) level) of TTITQ.

Atom	X	Y	Z
C	0.68147200	-3.97199800	-0.03658700
C	1.45812500	-2.75946200	0.02226500
C	0.67950000	-1.58109400	0.06447700
C	-0.78375400	-1.58512000	0.04117100
C	-1.56770500	-2.76669500	-0.01757000
C	-0.78597100	-3.97738800	-0.05650200
N	1.35792500	-0.38603100	0.13173400
C	0.65466700	0.70901100	0.16974400
C	-0.78074600	0.69868300	0.13989200
N	-1.46905500	-0.40115000	0.07823300
C	1.10807900	2.10573000	0.24662700
C	-0.03172300	2.94103400	0.25976500
C	-1.28142600	2.11809600	0.19492100
C	2.38823900	2.65072400	0.30437500
C	2.50880000	4.04238000	0.37307600
C	1.37729100	4.87130600	0.38458800
C	0.09219400	4.32414000	0.32836300
O	-2.43237200	2.51180400	0.19038300
N	-1.29648600	-5.21342700	-0.11652400
S	-0.04728500	-6.26588500	-0.14381500
N	1.19715600	-5.20771900	-0.08179000
C	2.91158700	-2.77003600	0.03357000
C	-3.01372700	-2.76999400	-0.03796000
S	3.91043700	-1.30596300	0.06967800
C	5.12441800	-3.58334700	0.01932300
C	3.74870700	-3.88285800	0.01458300
C	-3.85550600	-3.88470800	-0.06378700
C	-5.22518200	-3.58086700	-0.08330900
C	-5.49322800	-2.22075100	-0.07356100
S	-4.00405900	-1.30040500	-0.04369100
N	-10.60977600	0.32670800	-0.13515100
N	10.53529800	0.28839700	0.06838800
C	11.70423300	-0.46974500	-0.22391700
C	10.65797200	1.67422300	0.36883000
C	-11.78350200	-0.40954000	0.19495200
C	-10.73053300	1.70262400	-0.48509200
C	12.86721500	-0.32000100	0.54053000
C	14.01468500	-1.05449600	0.24029600

C	14.04922200	-1.97632800	-0.81492600
C	12.86980600	-2.11816100	-1.56726800
C	11.72376100	-1.38047600	-1.29176600
C	9.94330900	2.24669900	1.43276600
C	10.06943700	3.60184700	1.71760600
C	10.91770900	4.44557400	0.97878300
C	11.63117600	3.85460200	-0.07295900
C	11.50216500	2.50036300	-0.38235400
C	-10.04191000	2.22837700	-1.58900100
C	-10.16800000	3.57261100	-1.92142200
C	-10.99132900	4.44881800	-1.19222700
C	-11.67983000	3.90317700	-0.10012400
C	-11.54995000	2.56076000	0.25676200
C	-12.94545600	-0.28891900	-0.57565700
C	-14.09839300	-0.99830800	-0.23832100
C	-14.13921800	-1.86474600	0.86266800
C	-12.96062100	-1.97736400	1.62136100
C	-11.80896100	-1.26385400	1.30815200
C	5.39854200	-2.22949700	0.03739600
C	6.70398600	-1.57454700	0.04405600
C	-6.79296400	-1.56104500	-0.08626500
C	6.88140400	-0.25171900	-0.40719000
C	8.12855900	0.35792400	-0.40495900
C	9.26418900	-0.32954900	0.05983400
C	9.09883200	-1.65010600	0.51756600
C	7.84972100	-2.25378900	0.50594300
C	-6.94860800	-0.20542700	0.27078400
C	-8.19037700	0.41186200	0.26062300
C	-9.34561800	-0.29821700	-0.11809500
C	-9.20174200	-1.65108100	-0.48311300
C	-7.95769000	-2.26218100	-0.46335500
C	11.03049600	5.93709800	1.34014600
C	15.29458000	-2.80932200	-1.16567500
C	11.53882400	6.07926000	2.79474900
C	12.00591700	6.69048900	0.41605900
C	9.63977700	6.60455300	1.21798000
C	16.47917400	-2.51385400	-0.22630300
C	14.95667700	-4.31528100	-1.05463300
C	15.73775800	-2.49108100	-2.61375000
C	-11.10465100	5.92646700	-1.60634200
C	-11.65327900	6.01692600	-3.05045800
C	-12.04887500	6.72125400	-0.68453200

C	-9.70672600	6.58745600	-1.54866100
C	-15.39109100	-2.66842700	1.25602600
C	-16.57399900	-2.41162600	0.30324700
C	-15.06534400	-4.18076900	1.22193500
C	-15.83031900	-2.27289300	2.68613600
H	3.26323300	2.00833300	0.29808100
H	3.49743700	4.49092000	0.41912700
H	1.50451200	5.94884100	0.43875100
H	-0.79500800	4.95057700	0.33766900
H	5.89319800	-4.34745900	-0.02430400
H	3.36214900	-4.89000600	-0.01514300
H	-3.47074600	-4.89325500	-0.05926300
H	-5.99594900	-4.34371500	-0.07586400
H	12.87365700	0.37649200	1.37323900
H	14.89056900	-0.90310500	0.86164300
H	12.84254800	-2.81193400	-2.40322300
H	10.83620400	-1.50648100	-1.90410000
H	9.28979600	1.62332200	2.03519000
H	9.49967600	4.00409600	2.55098700
H	12.29812900	4.45283600	-0.68404100
H	12.06232300	2.07961400	-1.21163000
H	-9.40722100	1.57874100	-2.18371800
H	-9.61724100	3.93984100	-2.78306400
H	-12.32645200	4.52874900	0.50532200
H	-12.08926800	2.17478400	1.11626400
H	-12.94661600	0.36516000	-1.44209900
H	-14.97364900	-0.87157000	-0.86597500
H	-12.93857500	-2.62733200	2.49191700
H	-10.92201900	-1.36446600	1.92609400
H	6.03133200	0.29666400	-0.80389300
H	8.23416300	1.37092400	-0.77906300
H	9.95700300	-2.19446400	0.89771800
H	7.75045500	-3.26177600	0.89777400
H	-6.08455400	0.36991400	0.59136500
H	-8.27521800	1.45113400	0.55943500
H	-10.07330400	-2.21506200	-0.79799400
H	-7.88072300	-3.29740000	-0.78177400
H	11.61721300	7.13804600	3.07090900
H	10.86497100	5.59770000	3.51117600
H	12.52899200	5.62371900	2.90962000
H	12.05340200	7.74504900	0.71024600
H	11.68595800	6.65433400	-0.63157700

H	13.02214000	6.28475400	0.47785500
H	8.90519000	6.13998900	1.88426200
H	9.70138000	7.66812000	1.47966800
H	9.25748900	6.52801200	0.19360400
H	17.34007900	-3.12822700	-0.51312700
H	16.79048800	-1.46434700	-0.27961600
H	16.24030400	-2.74756100	0.81750900
H	15.83326400	-4.92373500	-1.30919500
H	14.14491700	-4.60182900	-1.73150400
H	14.64809700	-4.57331900	-0.03511000
H	15.99479100	-1.43101200	-2.72059700
H	16.62062200	-3.08413700	-2.88256100
H	14.95062800	-2.71843100	-3.34038200
H	-11.73266800	7.06510000	-3.36421300
H	-11.00285400	5.50387000	-3.76664000
H	-12.64926200	5.56477400	-3.12023600
H	-12.09772800	7.76426100	-1.01712700
H	-11.69945500	6.72239600	0.35426600
H	-13.06916700	6.32128500	-0.70234400
H	-8.99373200	6.09268600	-2.21641000
H	-9.76945900	7.64061500	-1.84923500
H	-9.29588500	6.54726300	-0.53343700
H	-17.43964500	-3.00339000	0.62156900
H	-16.87667500	-1.35825500	0.30296500
H	-16.33804300	-2.70042900	-0.72735500
H	-15.94662700	-4.76826000	1.50755500
H	-14.25544200	-4.43890800	1.91232200
H	-14.75963900	-4.49305700	0.21682400
H	-16.07811500	-1.20658800	2.73892900
H	-16.71809100	-2.84381900	2.98495800
H	-15.04471800	-2.46980700	3.42322400

Table S3. Cartesian coordinates for DFT optimized structure (at B3LYP/6-31g (d) level) of HTTITQ.

Atom	X	Y	Z
C	-1.12010300	-1.37523800	0.02942200
C	-1.54016500	-0.03378400	-0.24883900
C	-0.48743500	0.90839500	-0.30238200
C	0.92144600	0.50995200	-0.18993900
C	1.35625100	-0.82446300	0.02502600
C	0.28051400	-1.76962700	0.17162100

N	-0.81202100	2.24332100	-0.44388000
C	0.17251500	3.09163100	-0.50997800
C	1.55092500	2.69069300	-0.45522100
N	1.90915800	1.45381600	-0.29370700
C	0.12225900	4.55691300	-0.63870800
C	1.44697100	5.04483800	-0.68876900
C	2.42272100	3.91377200	-0.57930800
C	-0.95876700	5.43168800	-0.70879300
C	-0.69364400	6.79891500	-0.83551900
C	0.62202900	7.28254400	-0.89009500
C	1.70742500	6.40497500	-0.81543700
O	3.63714600	3.97659200	-0.58614300
N	0.42194700	-3.07444900	0.44420700
S	-1.07479200	-3.72945300	0.50792900
N	-1.96900700	-2.39858900	0.19161800
C	-2.94424400	0.30190500	-0.44871500
C	2.74998000	-1.21570500	0.09729500
S	-4.25995200	-0.69243900	0.17349900
C	-4.89927200	1.46438700	-1.12397400
C	-3.48154400	1.38889700	-1.11936700
C	3.24893300	-2.51139100	0.20043000
C	4.65799500	-2.62301900	0.26211300
C	5.27312500	-1.37701200	0.21648000
S	4.10384700	-0.08153800	0.07526300
N	10.81401800	0.04614700	0.46260800
N	-10.98548900	-0.89851400	0.44968200
C	-11.74206000	-1.54209700	-0.56953800
C	-11.60953600	-0.58056800	1.68851200
C	11.80071300	-0.93901100	0.74788300
C	11.21796200	1.39608900	0.25538200
C	-13.05058100	-1.13698900	-0.85803400
C	-13.79363800	-1.78297400	-1.84660400
C	-13.26469600	-2.84048200	-2.59887800
C	-11.94647400	-3.22857300	-2.30055100
C	-11.19966300	-2.60712000	-1.30587700
C	-11.40288000	0.65929300	2.30506500
C	-12.01413500	0.95887500	3.52291700
C	-12.86769100	0.05240500	4.16631700
C	-13.07143300	-1.18344000	3.52736600
C	-12.45507700	-1.50391300	2.32290600
C	10.52282500	2.45740200	0.84676300
C	10.92325000	3.77638400	0.63150900

C	12.03648700	4.09281500	-0.15901900
C	12.72858500	3.01311700	-0.73553400
C	12.33104100	1.69431300	-0.54537800
C	12.84099800	-0.67470800	1.64639200
C	13.81616500	-1.63744100	1.90855000
C	13.78933000	-2.90236100	1.30573000
C	12.73353100	-3.15223200	0.41114000
C	11.76418600	-2.19706200	0.12607300
C	-5.47582900	0.40524800	-0.43741900
C	-6.89368800	0.08664700	-0.21138400
C	6.69748300	-1.02925500	0.27072700
C	-7.34905600	-0.35071400	1.04718700
C	-8.68090700	-0.68033300	1.26521200
C	-9.62602100	-0.57143600	0.22985300
C	-9.18290500	-0.13552600	-1.03094400
C	-7.84435800	0.17231400	-1.24603200
C	7.23574100	-0.03112100	-0.56602500
C	8.57841800	0.31753200	-0.51178900
C	9.44740000	-0.30819000	0.40059800
C	8.91712800	-1.29238700	1.25436500
C	7.57492100	-1.64340600	1.18628200
C	-13.56482800	0.35531800	5.50425900
C	-14.05362300	-3.56716200	-3.70224700
C	12.50821800	5.53585200	-0.41002200
C	14.84342900	-3.98921600	1.58042700
C	-13.21874400	1.75776000	6.03949100
C	-15.09885300	0.27708800	5.31709000
C	-13.12608300	-0.68510700	6.56235200
C	11.62566900	6.57305200	0.30993400
C	13.95947400	5.70173100	0.10055600
C	-15.47325900	-2.99580800	-3.87883600
C	12.46341500	5.83626900	-1.92737000
C	15.91149200	-3.52597300	2.58918800
C	14.15068500	-5.24692500	2.15721600
C	15.55845400	-4.36096900	0.25939800
C	-13.30699400	-3.42534100	-5.05015400
C	-14.17994500	-5.06732400	-3.34434300
C	-5.65558300	2.60865200	-1.76435600
C	-5.12232500	4.00374300	-1.38775500
C	-5.93923000	5.14255100	-2.01127100
C	-5.40147300	6.53763300	-1.66747000
C	-6.21970700	7.68132900	-2.28075100

C	-5.67054700	9.06972500	-1.93722200
C	5.35763400	-3.96463300	0.28176300
C	4.99711500	-4.86227300	-0.91860400
C	5.71678500	-6.21642800	-0.88690000
C	5.36814800	-7.12036100	-2.07630400
C	6.08680600	-8.47555600	-2.04878700
C	5.73282600	-9.37187900	-3.23987900
H	-1.97716100	5.05879200	-0.65976400
H	-1.52206900	7.49999900	-0.89135100
H	0.79548700	8.35044300	-0.98855700
H	2.73257200	6.76191700	-0.85188000
H	-2.85124400	2.12881200	-1.58898000
H	2.59436500	-3.37034800	0.22215400
H	-13.48968600	-0.31326700	-0.30374600
H	-14.80319300	-1.43277800	-2.03200700
H	-11.48825300	-4.04908900	-2.84618600
H	-10.18979400	-2.94466600	-1.09415000
H	-10.75871600	1.39262400	1.82975100
H	-11.82154000	1.93224400	3.96070400
H	-13.71779100	-1.92776200	3.98471800
H	-12.62656100	-2.47339400	1.86558200
H	9.66230400	2.25051900	1.47542600
H	10.35067400	4.56378600	1.10907200
H	13.59513000	3.20066800	-1.36392600
H	12.88221200	0.88653200	-1.01684200
H	12.88753300	0.29049900	2.14107200
H	14.60179200	-1.38464200	2.61216300
H	12.66862800	-4.11285400	-0.09280200
H	10.97229600	-2.42094900	-0.58202100
H	-6.64331300	-0.43030600	1.86930300
H	-8.99878600	-1.02064700	2.24520500
H	-9.89265500	-0.05173100	-1.84737000
H	-7.52612000	0.47061400	-2.24012100
H	6.59321700	0.45649000	-1.29349400
H	8.96505500	1.07462900	-1.18572300
H	9.56128600	-1.76770700	1.98670200
H	7.18677500	-2.37244000	1.89067700
H	-13.73590800	1.92807800	6.99051600
H	-12.14422600	1.87063700	6.22344100
H	-13.53244200	2.54753500	5.34730800
H	-15.61103300	0.48321600	6.26508200
H	-15.41763100	-0.71321200	4.97515400

H	-15.44127300	1.01130300	4.57888000
H	-12.04298400	-0.64525900	6.72504800
H	-13.62180400	-0.48828500	7.52101000
H	-13.38053800	-1.70636400	6.25979300
H	11.99857900	7.58248900	0.10263100
H	10.58473200	6.52948600	-0.02997900
H	11.63687300	6.43396300	1.39702300
H	14.31345300	6.72446900	-0.07891200
H	14.64989500	5.01746200	-0.40382100
H	14.02088500	5.50463600	1.17697000
H	-15.99539100	-3.54258500	-4.67215000
H	-16.06902200	-3.09296000	-2.96404000
H	-15.45467400	-1.93784900	-4.16455000
H	11.44356100	5.73700300	-2.31609700
H	12.80501900	6.85992600	-2.12480000
H	13.10525800	5.15566400	-2.49671200
H	16.63777900	-4.33000800	2.75340900
H	16.46374000	-2.65161400	2.22627500
H	15.47248800	-3.27429800	3.56137300
H	14.88869400	-6.03497700	2.35163000
H	13.40384700	-5.65350900	1.46714000
H	13.64289400	-5.01636000	3.10072600
H	16.06860200	-3.49025500	-0.16818000
H	16.30844100	-5.14168300	0.43689200
H	14.85671500	-4.73858100	-0.49176500
H	-13.85311700	-3.94568000	-5.84671300
H	-12.29924400	-3.85143100	-5.00448600
H	-13.21130400	-2.37154400	-5.33583900
H	-14.71499100	-5.20020300	-2.39708400
H	-14.73379700	-5.60179800	-4.12605600
H	-13.20033000	-5.54645300	-3.24420200
H	-5.62835700	2.50689100	-2.86031800
H	-6.71192100	2.54926700	-1.48167500
H	-5.12188000	4.10409500	-0.29370500
H	-4.07485900	4.09756100	-1.70572800
H	-5.95870100	5.02090500	-3.10462200
H	-6.98477600	5.06432100	-1.67858500
H	-5.37435300	6.65789700	-0.57404600
H	-4.35775900	6.61790700	-2.00889600
H	-6.25016100	7.55933800	-3.37278600
H	-7.26110600	7.60447000	-1.93750800
H	-6.27686900	9.86364600	-2.38796000

H	-5.65927700	9.23471200	-0.85266300
H	-4.64228500	9.19050200	-2.30083300
H	6.44189100	-3.81767400	0.29385600
H	5.10861700	-4.49879900	1.21092500
H	3.91162100	-5.02806100	-0.94491300
H	5.24724300	-4.33199600	-1.84767100
H	6.80413400	-6.05001800	-0.86371300
H	5.47001000	-6.73783400	0.05023100
H	4.28089400	-7.28768700	-2.09962000
H	5.61402600	-6.59905900	-3.01344500
H	7.17322100	-8.30849600	-2.02640600
H	5.84086800	-8.99670300	-1.11249800
H	6.26160500	-10.33076500	-3.19141700
H	4.65722800	-9.58582200	-3.26908400
H	5.99924200	-8.89218200	-4.18984200

Table S4. Cartesian coordinates for DFT optimized structure (at B3LYP/6-31g (d) level) of OTTITQ.

Atom	X	Y	Z
C	0.34647400	0.00101600	-2.01009200
C	1.32202100	0.70944900	-1.23850200
C	0.81541400	1.48180800	-0.17630400
C	-0.62264900	1.60003200	0.08287400
C	-1.59712300	1.03213800	-0.77209400
C	-1.08534100	0.17646400	-1.79797200
N	1.72476300	2.13580400	0.63243200
C	1.23255100	2.79084100	1.64314100
C	-0.17677100	2.82774800	1.94238500
N	-1.07671100	2.25314200	1.20471100
C	1.94426000	3.56342400	2.67282100
C	0.99555400	4.06028700	3.59381400
C	-0.38488100	3.63576800	3.19717700
C	3.30093400	3.83131800	2.83597700
C	3.69191800	4.60477000	3.93347700
C	2.74966400	5.09912200	4.84782100
C	1.38794200	4.82982700	4.68372600
O	-1.43272300	3.91101700	3.75252000
N	-1.83950700	-0.55857600	-2.62651700
S	-0.80372900	-1.42189900	-3.55851200
N	0.62859200	-0.89281300	-2.96896100
C	2.73856800	0.58361900	-1.54961200
C	-3.01118400	1.27253900	-0.59310100

S	3.96914500	0.29674600	-0.32820000
C	4.73414600	0.32972000	-2.77664500
C	3.33170200	0.58674400	-2.79871900
C	-3.64656500	2.44612100	-0.20796000
C	-5.04891900	2.30369700	-0.01235400
C	-5.53629900	1.03891400	-0.29177400
S	-4.21603500	0.00284500	-0.79778100
N	-10.86707200	-1.01540200	0.00942800
N	10.62334200	-0.94887100	0.15838300
C	10.86642400	-1.52003100	1.43822200
C	11.72067400	-0.64750700	-0.69511100
C	-11.08669500	-2.42089700	0.05641900
C	-11.98271000	-0.13238800	0.04389800
C	11.91249700	-1.05801300	2.24611200
C	12.15531400	-1.63287700	3.49392500
C	11.36243100	-2.67280000	3.99783700
C	10.31167100	-3.11842100	3.17640100
C	10.06803600	-2.56833400	1.92294200
C	11.77193000	0.55548600	-1.40984400
C	12.84870300	0.83821200	-2.25068200
C	13.92375900	-0.04819800	-2.40188700
C	13.85978000	-1.24664100	-1.66929400
C	12.78525100	-1.55089500	-0.84107300
C	-12.05191600	0.97530200	-0.81553200
C	-13.14588100	1.83253800	-0.77304100
C	-14.22520600	1.62487600	0.10395400
C	-14.14372000	0.50846400	0.94738400
C	-13.04487400	-0.35084600	0.92915300
C	-12.10515300	-3.01475200	-0.69840800
C	-12.32591500	-4.39087800	-0.63670200
C	-11.53698100	-5.23285600	0.15892400
C	-10.51446500	-4.61972800	0.90438100
C	-10.29355600	-3.24742900	0.86775400
C	5.25620600	0.14389700	-1.51048700
C	6.63246800	-0.11904200	-1.09528900
C	-6.90313500	0.53152700	-0.22660500
C	7.60524700	-0.58987300	-2.00235500
C	8.90308400	-0.86524800	-1.59256600
C	9.29850900	-0.67176900	-0.25773600
C	8.34004800	-0.19324200	0.65235600
C	7.04131300	0.07087100	0.24041800
C	-7.90691800	1.19270400	0.51291700

C	-9.19647600	0.68570600	0.59420200
C	-9.55235200	-0.50168500	-0.07007600
C	-8.56199600	-1.16233200	-0.81945300
C	-7.27222500	-0.65709600	-0.89000500
C	15.12977100	0.23698200	-3.31407000
C	11.59709900	-3.32130700	5.37336600
C	-15.41812600	2.59684700	0.10539100
C	-11.74682100	-6.75504200	0.24073800
C	15.01659000	1.59932900	-4.02412900
C	16.42702700	0.24086600	-2.47063000
C	15.22908800	-0.86330100	-4.39763400
C	-16.07502800	2.61035700	-1.29561500
C	-16.49366900	2.20097800	1.13471900
C	12.79056000	-2.69469700	6.11909400
C	-14.92322300	4.02170800	0.45073200
C	-12.91233500	-7.23297500	-0.64591700
C	-10.46060700	-7.47917500	-0.22374500
C	-12.05867300	-7.15600600	1.70242600
C	10.33485000	-3.14355500	6.25058900
C	11.88304900	-4.83109600	5.19087600
O	5.48604200	0.31627200	-3.92577500
C	4.68904100	0.20922300	-5.11014900
C	3.52235700	1.18089400	-5.04900200
O	2.65952200	0.85921900	-3.95546800
O	-3.01589700	3.64530500	-0.09292500
C	-3.90156900	4.76308600	0.02876200
C	-5.05803300	4.42470600	0.95258900
O	-5.83118900	3.35572200	0.39086500
H	4.02775100	3.44775200	2.12663000
H	4.74502300	4.82798300	4.08189100
H	3.08533800	5.69651100	5.69090100
H	0.64503400	5.20365500	5.38241100
H	12.54065500	-0.24538900	1.89460600
H	12.97811700	-1.23996900	4.08121700
H	9.66949300	-3.92824500	3.51225500
H	9.25566500	-2.94880800	1.31162200
H	10.96329500	1.27266200	-1.30860900
H	12.84111200	1.78304400	-2.78317800
H	14.66203300	-1.97485600	-1.75394500
H	12.76503200	-2.49231700	-0.30095600
H	-11.24354700	1.16019900	-1.51614800
H	-13.15972400	2.67768500	-1.45606200

H	-14.94127900	0.29646600	1.65106200
H	-13.01099300	-1.19932300	1.60548000
H	-12.72883800	-2.39552400	-1.33565000
H	-13.12745100	-4.80322600	-1.23981500
H	-9.87737400	-5.22282600	1.54566600
H	-9.50327600	-2.80831900	1.46867300
H	7.33089200	-0.75739000	-3.03635600
H	9.62270300	-1.24225600	-2.31214000
H	8.62293400	-0.02009900	1.68554100
H	6.33541000	0.45999900	0.96938300
H	-7.66253900	2.10388200	1.04434600
H	-9.94003800	1.20846300	1.18690100
H	-8.81432900	-2.07016900	-1.35728700
H	-6.54214000	-1.18256500	-1.49983600
H	15.89446100	1.75835900	-4.66057900
H	14.12973500	1.65248300	-4.66588200
H	14.97220500	2.42928200	-3.30966700
H	17.29800500	0.43552100	-3.10866500
H	16.58982100	-0.71894400	-1.96916100
H	16.39002000	1.01845600	-1.69910700
H	14.32684500	-0.88273400	-5.01956800
H	16.09003400	-0.67944100	-5.05230000
H	15.35223400	-1.85854200	-3.95762900
H	-16.92324200	3.30587500	-1.31386100
H	-15.37061100	2.92429900	-2.07311000
H	-16.44596100	1.61447400	-1.56366000
H	-17.32079500	2.91902300	1.09941600
H	-16.09954900	2.20170400	2.15739700
H	-16.90967100	1.20811000	0.92890200
H	12.91915200	-3.18667400	7.08987100
H	13.72725800	-2.81309000	5.56247300
H	12.63771100	-1.62603900	6.30848400
H	-14.18203000	4.38149700	-0.27067600
H	-15.76167600	4.72920300	0.44827700
H	-14.46093700	4.04554700	1.44418500
H	-13.02429600	-8.31931100	-0.55485600
H	-13.86400600	-6.77715800	-0.34960000
H	-12.73926300	-7.00677800	-1.70426800
H	-10.59096000	-8.56682100	-0.16404700
H	-9.59719700	-7.21297100	0.39497600
H	-10.21943800	-7.22198400	-1.26148100
H	-12.97261900	-6.66554300	2.05655600

H	-12.20288400	-8.24082900	1.77899500
H	-11.24659500	-6.87970200	2.38315400
H	10.48301400	-3.60808800	7.23334600
H	9.45305300	-3.60508200	5.79388400
H	10.11269800	-2.08165000	6.40638800
H	12.78044000	-4.98874900	4.58170300
H	12.04385900	-5.31064800	6.16439000
H	11.05141800	-5.34689700	4.69939400
H	5.35449200	0.44094100	-5.94565100
H	4.31634000	-0.81860700	-5.21658500
H	2.90715000	1.11636300	-5.95012300
H	3.89566200	2.20880100	-4.94035500
H	-3.29964900	5.58169000	0.43088000
H	-4.28121700	5.04501100	-0.96298700
H	-5.74187900	5.27003300	1.05978900
H	-4.67568100	4.13233600	1.93938700

Table S5. The values of orbital states S_1-S_5 and T_1-T_5 of TTITQ, HTTITQ and OTTITQ, unit: eV.

TTITQ			
S_1	1.0853	T_1	0.4542
S_2	1.4700	T_2	1.2257
S_3	1.7807	T_3	1.4948
S_4	1.8193	T_4	1.5606
S_5	2.1595	T_5	2.0452
HTTITQ			
S_1	1.1708	T_1	0.5680
S_2	1.4314	T_2	1.2790
S_3	1.7218	T_3	1.4731
S_4	1.8882	T_4	1.6392
S_5	2.1660	T_5	2.0195
OTTITQ			
S_1	1.1962	T_1	0.7110
S_2	1.4921	T_2	1.2857
S_3	1.8558	T_3	1.5899
S_4	1.9202	T_4	1.6823
S_5	2.1361	T_5	1.9929

Table S6. The values the spin-orbit coupling (SOC) constant of TTITQ, HTTITQ and OTTITQ.

TTITQ		HTTITQ		OTTITQ	
S_n-T_n	$\zeta(S_n$-$T_n)$ (cm^{-1})	S_n-T_n	$\zeta(S_n$-$T_n)$ (cm^{-1})	S_n-T_n	$\zeta(S_n$-$T_n)$ (cm^{-1})
S_0-T_1	0.217408985	S_0-T_1	0.302489669	S_0-T_1	1.05279
S_1-T_1	0.135277493	S_1-T_1	0.236290781	S_1-T_1	0.558808
S_1-T_2	0.074833148	S_1-T_2	0.422926314	S_1-T_2	0.482135
S_1-T_3	0.242418371	S_1-T_3	0.716426316	S_1-T_3	0.639687
S_1-T_4	0.258069758	S_1-T_4	0.439393521	S_1-T_4	0.647894
S_1-T_5	1.082266141	S_1-T_5	1.128819442	S_1-T_5	1.82949

3. Supplementary References

[1] Frisch M J, Trucks, G W, Schlegel, H B *et al*. *Gaussian 09*, Gaussian, Inc., Wallingford CT, 2009.

[2] Shuai Z. Thermal vibration correlation function formalism for molecular excited state decay rates. *Chin J Chem* 2020; **38**: 1223-1232.

[3] Shuai Z, Peng Q Excited states structure and processes: Understanding organic light-emitting diodes at the molecular level. *Phys Rep* 2014; **537**: 123-156.

[4] Shuai Z, Peng Q. Organic light-emitting diodes: theoretical understanding of highly efficient materials and development of computational methodology. *Natl Sci Rev* 2017; **4**: 224-239.

[5] Hess B, Kutzner C, Van Der Spoel D *et al*. GROMACS 4: algorithms for highly efficient, load-balanced, and scalable molecular simulation. *J Chem Theory Comput* 2008; **4**: 435-447.

[6] Yang Z, Zhang Z, Lei Z *et al*. Precise molecular engineering of small organic phototheranostic agents toward multimodal imaging-guided synergistic therapy. *ACS nano* 2021; **15**: 7328-7339.

[7] Yan D, Xie W, Zhang J *et al*. Donor/π‐Bridge manipulation for constructing a stable NIR‐II aggregation‐induced emission luminogen with balanced phototheranostic performance. *Angew Chem Int Ed* 2021; **133**: 26973-26980.

[8] Li J, Wang J, Zhang J *et al*. A facile strategy of boosting photothermal conversion efficiency through state transformation for cancer therapy. *Adv Mater* 2021; **33**: 2105999.

[9] Sun C, Li B, Zhao M *et al*. J-aggregates of cyanine dye for NIR-II in vivo dynamic vascular imaging beyond 1500 nm. *J Am Chem Soc* 2019; **141**: 19221-19225.

[10] Jacques B, Tranchier J P, Rose-Munch F *et al*. Cationic (η^6-Arene) tricarbonylmanganese Linked to Ferrocene Complexes. *Organometallics* 2004; **23**: 184-193.

[11] Khlyabich P P, Rudenko A E, Thompson B C. Random poly (3-hexylthiophene-co-3-cyanothiophene) copolymers with high open-circuit voltage in organic solar cells. *J Polym Sci Part A: Polym Chem* 2014; **52**: 1055-1058.

[12] Patel D G, Feng F, Ohnishi Y *et al*. It takes more than an imine: the role of the central atom on the electron-accepting ability of benzotriazole and benzothiadiazole oligomers. *J Am Chem Soc* 2012; **134**: 2599-2612.

[13] Ozelcaglayan A C, Sendur M, Akbasoglu N *et al.* Synthesis and electrochemical properties of a new benzimidazole derivative as the acceptor unit in donor–acceptor–donor type polymers. *Electrochim Acta* 2012; **67**: 224-229.

[14] Steckler T T, Abboud K A, Craps M, et al. Low band gap EDOT-benzobis (thiadiazole) hybrid polymer characterized on near-IR transmissive single walled carbon nanotube electrodes. *Chem Commun* 2007; **46**: 4904-4906.

[15] Ding C, Xu Z, Zhang W *et al*. CN111196821A 2020-05-26.
