# Supplementary material for: Unveiling photophysical mechanisms of NIR-II AIE luminogens for multimodal imaging-navigated synergistic therapies
Source: Natl Sci Rev. 2025 Jun 24;12(8):nwaf254. doi: 10.1093/nsr/nwaf254 (PMC12409622; doi:10.1093/nsr/nwaf254)
Supplement: nwaf254_Supplemental_Files [file nwaf254_supplemental_files.zip › Teaser text.docx]

Teaser text

Guided by theoretical studies, a multifunctional molecule with emission in the second near-infrared window is designed and synthesized for multimodal imaging-navigated synergistic therapies towards bladder cancer.
